# Identifying candidate genetic variants for egg number by analyzing over 1,000 fully sequenced layers

**DOI:** 10.1093/gigascience/giaf064

**Published:** 2025-06-17

**Authors:** Aixin Ni, Henk Bovenhuis, Mario P L Calus, Yunlei Li, Jingwei Yuan, Yanyan Sun, Jilan Chen

**Affiliations:** State Key Laboratory of Animal Biotech Breeding, Key Laboratory of Animal (Poultry) Genetics Breeding and Reproduction of Ministry of Agriculture and Rural Affairs, Institute of Animal Science, Chinese Academy of Agricultural Sciences, Beijing 100193, China; Animal Breeding and Genomics, Wageningen University and Research, Wageningen AH 6700, The Netherlands; Animal Breeding and Genomics, Wageningen University and Research, Wageningen AH 6700, The Netherlands; Animal Breeding and Genomics, Wageningen University and Research, Wageningen AH 6700, The Netherlands; State Key Laboratory of Animal Biotech Breeding, Key Laboratory of Animal (Poultry) Genetics Breeding and Reproduction of Ministry of Agriculture and Rural Affairs, Institute of Animal Science, Chinese Academy of Agricultural Sciences, Beijing 100193, China; State Key Laboratory of Animal Biotech Breeding, Key Laboratory of Animal (Poultry) Genetics Breeding and Reproduction of Ministry of Agriculture and Rural Affairs, Institute of Animal Science, Chinese Academy of Agricultural Sciences, Beijing 100193, China; State Key Laboratory of Animal Biotech Breeding, Key Laboratory of Animal (Poultry) Genetics Breeding and Reproduction of Ministry of Agriculture and Rural Affairs, Institute of Animal Science, Chinese Academy of Agricultural Sciences, Beijing 100193, China; State Key Laboratory of Animal Biotech Breeding, Key Laboratory of Animal (Poultry) Genetics Breeding and Reproduction of Ministry of Agriculture and Rural Affairs, Institute of Animal Science, Chinese Academy of Agricultural Sciences, Beijing 100193, China

**Keywords:** whole-genome sequencing, genetic variants, egg number, additive-dominance model, multiomics, TWAS, heterosis

## Abstract

**Background:**

Egg production over a long laying cycle until 700 days of age is preferred for modern layer chicken breeding. It is influenced by the onset of laying, stability during the peak period, and persistence at late laying stages. Conventional single-single nucleotide polymorphism (SNP) association analyses have identified additive loci, but few studies have explored dominance effects or integrated multiomics data to investigate the genetic basis of egg production traits from the onset to 700 days of age. A full diallel cross of 1,004 chickens was·subjected to whole-genome sequencing. Transcriptome data from the ovary were available for a subset of 120 chickens. A genome-wide association study (GWAS) was conducted using an additive-dominance model for cumulative egg number and egg number at different stages. Expression quantitative trait loci (eQTL) mapping was applied to investigate associations between SNPs and gene expression. A transcriptome-wide association study (TWAS) was conducted to explore the associations between gene expression and egg production traits to identify candidate genes.

**Results:**

The additive-dominance model identified 5,892 significant SNPs, comprising 805 additive SNPs and 360 dominance SNPs shared between 2 or more traits. By integrating loci identified through GWAS with eQTL mapping, the expression level of 27 genes was associated with significant SNPs. Further integration with TWAS results revealed 4 novel candidate genes. For the loci with significant SNP effects, we found a positive but insignificant correlation between the ratios of dominance to additive effects and observed heterosis. Observed heterosis was positively correlated with heterosis predicted based on dominance effects and allele frequencies of all SNPs.

**Conclusions:**

We identified candidate genetic variants for egg production traits by analyzing 1,004 fully sequenced layers. Detection benefited from incorporating dominance into the GWAS model. Traits with higher heterosis tended to be more affected by genes with a dominant mode of action. Moreover, multiomics data allowed for the contribution to deciphering genetic mechanisms underlying egg production by establishing connections between genetic variants, gene expression, and egg number.

## Graphical Abstract

**Graphical Abstract:**
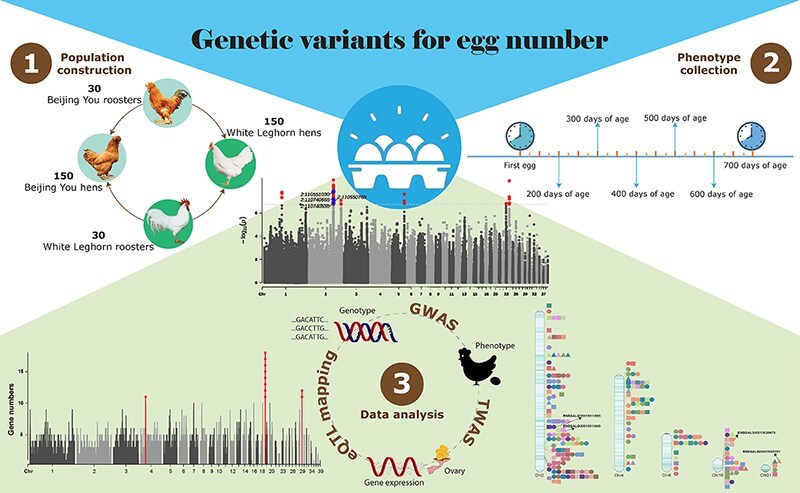

## Background

The global egg production (in tons) has doubled since 1990, and eggs play crucial roles in providing high-quality and low-cost animal protein for the growing population [1]. Egg production is not only a reflection of laying efficiency and economic efficiency but also one of the most important breeding goal traits for laying hens. Combining genomic information and knowledge of the genetic architecture of traits is of increasing importance in selective breeding. Nowadays, to reduce brooding costs and to lower environmental impact, there is a trend to extend the laying cycle from the traditional 500 to 700 days of age [2]. This emphasizes the need for studies to look for loci underlying egg production traits throughout the laying period, especially during the extended period, to provide insights for selective breeding.

Quantitative trait loci (QTL) mapping and genome-wide association study (GWAS) have revealed genomic variants statistically associated with egg production traits. About 115 QTL on 27 chromosomes were reported to be associated with egg number in chickens [3]. While GWAS establishes the connection between genotype and phenotype, the underlying biological mechanisms remain unclear. Furthermore, most of the identified variants are located in noncoding regions, and several variants are in high linkage disequilibrium (LD), making it difficult to determine the causal variants. These issues can be alleviated by using multiomics strategies such as expression quantitative trait loci (eQTL) mapping, transcriptome-wide association study (TWAS), and Mendelian randomization (MR) analysis to unravel the underlying genetic architecture of complex traits. eQTL mapping identifies genomic regions associated with the expression levels of genes based on single-nucleotide polymorphism (SNP) genotypes and gene expression data. TWAS establishes a connection between gene expression and phenotypes by predicting gene expression levels in genotyped animals. It then leverages the summary-level GWAS results and expression data to identify the expression of gene and phenotype associations. MR provides evidence for putative causal relations between gene expression and phenotypes [4]. The joint analysis of genomic and transcriptomic data has contributed to deciphering the biological functions of candidate genes for various traits in cattle [5], pigs [6, 7], and chickens [8].

Another limitation of many GWAS studies is that nonadditive effects are often ignored. Dominance is believed to be common in mammals [9] and has been studied in the context of genetic parameter estimation, genomic selection, and genomic prediction in several farm animals for traits such as carcass weight of cattle [10], body weight of quails [11], reproductive performance of dairy cattle [12], and growth of tilapia [13]. In chickens, Amuzu‐Aweh [14] found that dominance variance accounted for up to 37% of the genetic variance and up to 6% of the phenotypic variance in egg number depending upon the line, highlighting the substantial role of nonadditive genetic effects. Furthermore, loci with dominance effects have been identified in several species for different traits, contributing to phenotypic variance. In cattle, 1 key genetic variant of sperm motility demonstrated a much higher significance of the nonadditive effects compared to additive effects (*P* = 1.0E-31 for dominance and *P* = 1.1E-08 for additive) [15]. Dominance loci could explain 12% to 13% of phenotypic variance in sheep’s resistance to *Haemonchus contortus* and 0.69% to 0.84% of phenotypic variance in broilers’ egg number [16, 17]. These examples underscore the importance and potential of considering dominance effects to gain a more comprehensive understanding of the genetic architecture underlying complex traits.

**Figure 1:**
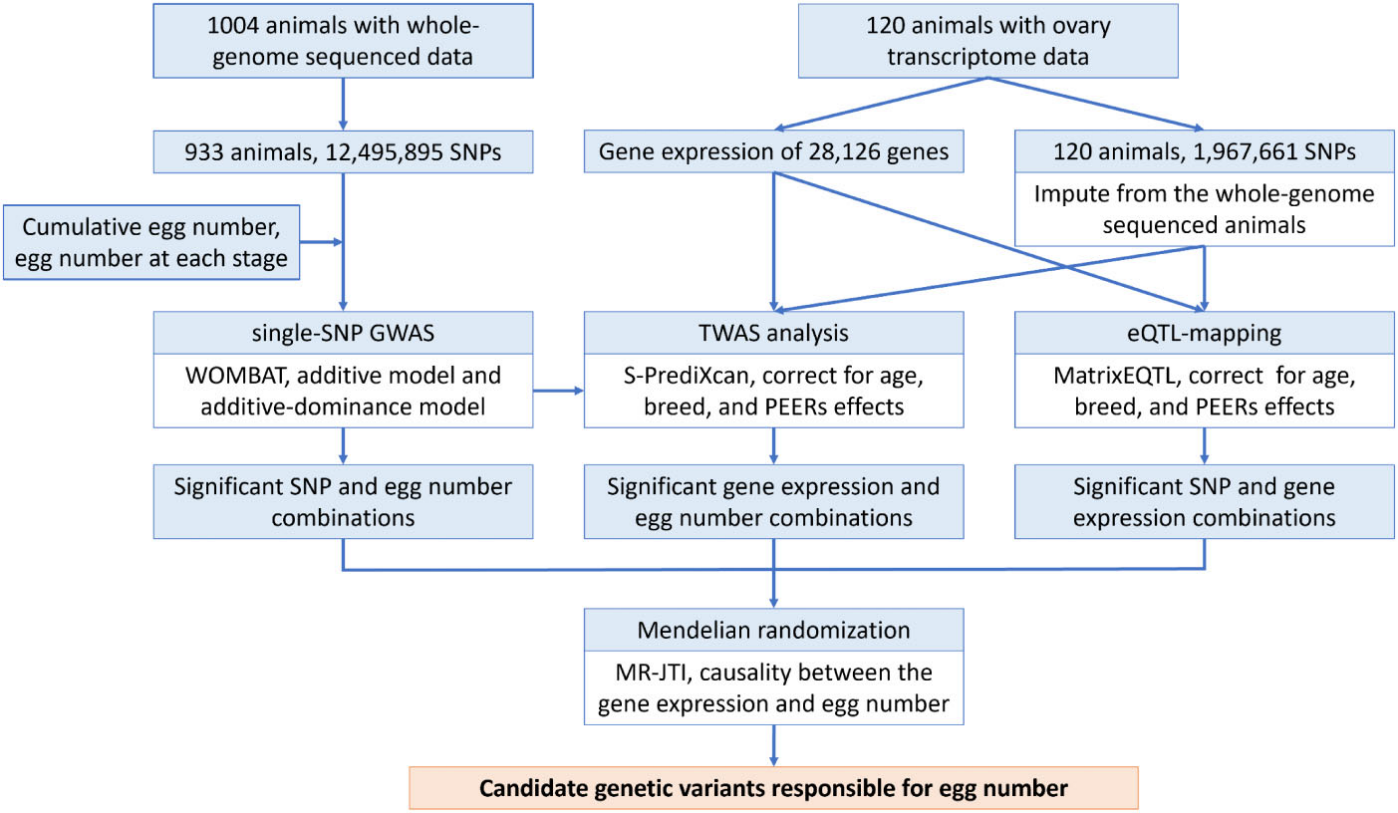
Framework for identification of SNPs and genes for egg production traits using multiomics data.

Examining the role of SNPs, particularly nonadditive effects, might provide valuable insights into heterosis, which is thought to be driven by nonadditive genetic effects [18]. Quantitative genetic theory suggests that heterosis, expressed as the difference between the crossbreds and the mid-parent value, is proportional to the sum of the dominance effects multiplied by the squared difference in allele frequency between the parental lines [18]. Moreover, the integration of genomic and transcriptomic data has led to the identification of heterosis-related genetic variants in several plants. For instance, *RH8* was identified as a heterosis-related candidate gene for yield in rice [19], structural variants in *ZAR1* and *ZmACO2* were found to increase heterosis for yield in maize [20], and a CACTA-like transposable element upstream of *BnaA9.CYP78A9* was shown to contribute to the heterosis of cell number in oilseed rape [21]. In the current study, we aimed to identify additive and nonadditive candidate genetic variants for egg number, explore the genetics underlying these complex traits using multiomics data, and discuss implications for heterosis. To achieve our objective, we sequenced the genome of 1,004 animals, which enabled including all genome-wide segregating variants in the analysis and would increase the power to detect variants associated with egg production. We employed a model incorporating both additive and dominance SNP effects and combined this with transcriptome data of ovary tissue to map eQTLs. These analyses were followed by a TWAS to prioritize candidate genes for egg production traits. The flowchart of analyses to identify candidate genetic variants for egg production traits is shown in Fig. 1. Estimated dominance SNP effects were used to investigate its relationship with observed heterosis.

## Methods

### Resource population and phenotypic data

Animal resources used in our study were previously described in detail [22]. Briefly, 4 genetic groups were used: pure-line Beijing-You chickens (YY) and White Leghorns (WW), as well as their reciprocal crosses with either Beijing-You (YW) or White Leghorns (WY) as the sire line. The YW and YY animals were created using the same 30 Beijing-You sires, and the WY and WW were created using the same 30 White Leghorn sires. The chickens were kept in individual cages in the same hen house during the experiment. Cumulative egg number and egg number at different stages from 200 to 700 days of age were recorded: CEN200 (cumulative egg number until 200 days of age), CEN300, CEN400, CEN500, CEN600, CEN700, EN300 (egg number from 200 to 300 days of age), EN400, EN500, EN600, EN700, EN300_500, and EN500_700 were computed from individual egg-laying recordings. In the current study, we defined the time period before 300 days of age as the early stage, 300 to 500 days of age as the middle stage, and 500 to 700 days of age as the late stage. Observed heterosis was estimated for different traits based on the predicted mean phenotypes for each genetic group, using the “predict” statement in ASReml 4.2 following the model described previously [22].

### Whole-genome sequencing

Genomic DNA was extracted using the phenol-chloroform method. The genomes of 1,004 chickens were sequenced at ∼15.98× coverage (Supplementary Fig. S1), containing 210 WW, 240 WY, 268 YY, and 286 YW. After sequencing, FastQC (RRID:SCR_014583) was used to evaluate the quality of sequencing [23]. Trimmomatic (RRID:SCR_011848) was used to remove adapters and the low-quality reads with the following parameters: LEADING:3 TRAILING:3 SLIDINGWINDOW:4:15 and MINLEN:15 [24].

Clean reads were aligned using BWA-MEM (v0.7.17, RRID:SCR_022192) [25]. Samblaster (v0.1.26, RRID:SCR_000468) was used to mark duplicates [26] and Samtools (v1.14, RRID:SCR_002105) to sort and index the BAM files [27]. Freebayes (v1.3.1, RRID:SCR_010761) was used with the chicken reference genome (GRCg7W) [28] for variant calling with the following: –use-best-n-alleles 4 –min-base-quality 10 –min-alternate-fraction 0.2 –haplotypelength 0 –ploidy 2 –min-alternate-count 2 [29]. The vcffilter module from vcflib (v0.00.2019.07.10, RRID:SCR_001231) was used to discard variants with low phred quality score (≤20) [30]. Tabix, a module from htslib (v1.9), was used to index the VCF files [30]. Alignment quality control statistics were computed with QualiMap (v.2.2.2-dev, RRID:SCR_001209) [31].

A total of 16,828,475 variants were called. After removing indels with Plink (v1.9, RRID:SCR_001757) [32], 14,119,765 SNPs were retained. The data were further filtered with Plink using the following criteria: genotyping call rate for SNPs <95% and for individuals <95%, minor allele frequency <0.5%, and, only for pure-line individuals, a test for Hardy–Weinberg equilibrium *P* < 1.0E-04. The cutoff of 0.5% for minor allele frequency corresponds to a requirement that for each locus, the minor allele should be observed at least 10 times in the data. After those filters, 12,495,895 SNPs and 986 animals were retained.

Due to missing genotypes from the parents, it is not possible to compare the parent–offspring relationship. Instead, we compared pedigree and genomic relationships among the 986 animals. We removed conflicting animals manually using the following steps, which were performed within WW, YY, and combined crossbred genetic groups (WY and YW): calculated pedigree and genomic relationships and sorted the animals within the genetic groups based on descending absolute difference between pedigree and genomic relationships. In the top 100 of those relationships, we counted the occurrences for each animal. Starting from the top, we removed from all of those 100 relationships the animal that was involved in the largest number of “conflicts.” This process improved the credibility of the genomic data (Supplementary Figs. S2–S7). The principal component analysis (PCA) revealed that the grouping based on the first 2 principal components, which accounted for 63.47% (PC1) and 5.59% (PC2) of the total variance for the animals retained after quality control, coincides with the 4 genetic groups (Supplementary Fig. S8). After quality control, 933 animals were retained for subsequent analysis. Beagle (v.4.1, RRID:SCR_001789) was used to impute missing genotypes [33]. To avoid confounding between genetic group and genotype in the statistical analysis, SNPs were eliminated if 1 genotype class was observed in both WW and YY lines with 5 or fewer animals.

### Variance components estimation

Additive and dominance variances were estimated with the restricted maximum likelihood method. For each trait, 2 models were fitted in Wombat [34]: one with only additive effects (model A) and the other with additive and dominance effects (model AD). Model A was


\begin{eqnarray*}
y = {X_1}{b_1} + u + e
\end{eqnarray*}

where **y** was the vector with phenotypic values; **b_1_** was a vector of the fixed effects, including genetic group (WW, YY, YW, and WY) and rack effects; **X_1_** was the corresponding design matrix; **u** was the vector of the random animal effects with $N(0,G\sigma _a^2)$, where **G** was the genomic relationship matrix and $\sigma _a^2$ was the additive genetic variance; and **e** was the vector of random residual effects with $N( {0,I\sigma _e^2} )$, where **I** was the identity matrix and $\sigma _e^2$ was the residual variance.

Model A was extended with a dominance deviation as model AD:


\begin{eqnarray*}
y = {X_1}{b_1} + u + v + e
\end{eqnarray*}

where **v** was a vector of random dominance deviations with $N( {0,D\sigma _d^2} )$, where **D** was the dominance relationship matrix and $\sigma _d^2$ was the dominance variance.

The genomic relationship matrix was computed with program Calc_grm according to the first version of VanRaden [35], $G = \frac{{Z{Z^{\prime}}}}{{2\sum\nolimits_{{p_i}} {{p_i}{{(1 - {p_i})}^{\prime}}} }}$; **Z** was the matrix of SNP genotypes (coded as 0, 1, 2) for individuals with phenotypes for each trait; and *p_i_* was the frequency of the counted alleles at SNP *i*. The dominance relationship matrix was computed with program Calc_grm according to Vitezica et al. [36], $D = \frac{{M{M^{\prime}}}}{{\sum\nolimits_{_i} {{{( {2{p_i}(1 - {p_i})} )}^2}} }}$, where **M** was a matrix of heterozygote coefficients (coded as 0, 1, 0) for individuals with phenotypes for each trait. When individual *j* was homozygous for locus *i*, ${M_{ij}} = {\mathrm{0}} - {\mathrm{2}}{p_i}\ (1 - {p_i})$, and when it was heterozygous, ${M_{ij}} = {\mathrm{1}} - {\mathrm{2}}{p_i}\ (1 - {p_i})$.

### Genome-wide association study

A single SNP GWAS was performed to estimate additive and dominance effects per SNP. For each SNP, the following model was fitted for model A:


\begin{eqnarray*}
y = {X_1}{b_1} + j\alpha + u + e
\end{eqnarray*}

where **j** was a vector with allele counts (coded as 0 for homozygotes of the reference allele, 1 for heterozygotes, and 2 for homozygotes of the alternative allele); *α* was the additive effect.

Model AD was extended with additive and dominance SNP effects:


\begin{eqnarray*}
y = {X_1}{b_1} + j\alpha + k\beta + u + v + e
\end{eqnarray*}

where **k** was a vector representing heterozygosity status, coded as 0 for homozygotes of the reference allele, 1 for heterozygotes, and 2 for homozygotes of the alternative allele; *β* was the dominance effect. The genomic relationship matrix was computed using the same method as described above. SNPs on the sex chromosomes were excluded from the dataset, as model AD does not allow for differentiation between additive and dominance SNP effects in the genotype matrix when only 1 allele is observed at a locus, which is the case for the ZW females.

Solutions and *t*-statistics of the SNP effects were obtained from the output of Wombat, and corresponding *P*-values were computed. The genome-wide significance threshold for the SNP effects was based on a false discovery rate (FDR). FDR was calculated using the R-package “qvalue” (RRID:SCR_001073), and FDR < 0.01 was considered significant. Manhattan and Q–Q plots were derived from the GWAS results using the R-package “CMplot” (RRID:SCR_024514) [37]. The variant effect predictor (VEP; RRID:SCR_007931) software [38] was used to predict the maximal consequence of the significant SNPs. For comparison, we also calculated the maximal consequences of all SNPs. The SNP ratio was calculated based on the *t*-statistics from the model AD, $r = | {\frac{{{t_{Dom}}}}{{{t_{Add}}}}} |$ [9]. The *t*-statistics (t_Dom_ and t_Add_) is the ratio of the estimated SNP effect and its standard error. Based on the SNP ratios, SNPs were considered additive (*r* < 0.2), partial dominance (0.2 < *r* < 0.8), complete dominance (0.8 < *r* < 1.2), or over-dominance (*r* > 1.2) [9]. In addition to the SNP ratios, we calculated the sum of the dominance effects (d) multiplied by the squared difference in allele frequency (y) between the parental lines (dy^2^) for all SNPs and significant SNPs to investigate the relation with observed heterosis. To enable comparison across different traits, we standardized the dominance SNP effects based on the phenotypic standard deviation of the trait.

### Transcriptome sequencing

From each of the 4 genetic groups, 6 chickens were randomly selected at 150, 250, 320, 500, and 700 days of age to collect ovaries for RNA sequencing, yielding 120 samples in total. Total RNA was isolated from the tissue of each hen using TRIzol Reagent (Invitrogen) according to the manufacturer’s guidelines. RNA sequencing (RNA-seq) was performed using Novaseq 6000 (Illumina) to generate 150-bp paired-end reads. Quality control, mapping, and transcriptome assembly were done following the steps described previously [39], getting the transcripts per kilobase per million mapped reads (TPM) for each gene when mapped to the chicken reference genome (GRCg7W).

Of the 120 samples with transcriptome sequencing data, genotypic data for 67 of the samples were obtained from whole-genome sequencing. Genotypic data for the remaining 53 animals were obtained based on transcriptome sequencing data, and missing genotypes were imputed using the whole-genome sequencing data as a reference panel. For RNA-seq, STAR (v.2.7.11a, RRID:SCR_004463) was used to map the high-quality reads to the chicken reference genome (GRCg7W) with an average mapping rate of 93.76% (Supplementary Fig. S9) [40]. Picard (v. 2.7.1, RRID:SCR_006525) was used to sort the BAM files, mark duplicates, and reorder BAM files [41]. Samtools (v. 1.14) was used to index the BAM files [27]. GATK (v. 4.2.6.1, RRID:SCR_001876) was used to split the overlapping intron reads and detect variants [42]. The CombineGVCFs function was then used to jointly genotype all these samples into 1 GVCF per tissue. “GenotypeGVCFs” was used to transfer the GVCF to VCF file, and SNPs were extracted using SelectVariants and filtered with “QD < 2.0 || MQ < 40.0 || FS > 60.0 || SOR > 3.0 || MQRankSum < −12.5 || ReadPosRankSum < −8.0.” Beagle (v. 4.1) was used to impute the missing genotype using the whole-genome sequencing data as the reference panel [33]. To evaluate the imputation accuracy, we used the 67 animals that had genotypes based on transcriptome sequencing data and whole-genome sequencing. Using genotypes from the transcriptome sequencing, we imputed missing genotypes, using the whole-genome sequence data across the 3 genetic groups (WW, YY, and YW/WY) or only data from the genetic group to which the animal belonged. We calculated the concordance using the “pdiff” function in Plink and found a very similar distribution of concordance for SNPs and individuals in both cases (imputation based on all groups or their own genetic group—Supplementary Fig. S10). Based on these results, we decided to do the imputation for the 53 animals using whole-genome sequence data of all 3 genetic groups. Subsequently, when using the 67 animals, we evaluated the impact of removing SNPs with low quality from the transcriptome sequencing on the concordance between the 2 data sources. For the 67 animals, around 30% of the SNPs showed a high concordance (>90% matching rate between whole-genome sequencing and RNA data source). Removing SNPs from the transcriptome sequencing data with a call rate <90% considerably increased the percentage of SNPs (around 65.32%) that showed a high concordance with the 2 data sources (>90%; Supplementary Fig. S11). We removed SNPs with a call rate below 90% and excluded SNPs on the sex chromosomes, resulting in 1,967,661 SNPs for subsequent analysis.

### eQTL mapping

MatrixEQTL (RRID:SCR_025513) was used to carry out the eQTL analysis for the ovary tissue using the following model [43]:


\begin{eqnarray*}
y = {X_2}{b_2} + j\alpha + e
\end{eqnarray*}

where **y** was the TPM from the ovary tissue per gene; **b_2_** was a vector of the fixed effects, including genetic group, age, and probabilistic estimation of expression residual (PEER) effects calculated from R-package “peer” (RRID:SCR_009326) [44], where genetic group, age, and PEER were modeled as a linear regression; **X_2_** was the corresponding design matrix; **j** was a vector with allele counts (coded as 0 for homozygotes of the reference allele, 1 for heterozygotes, and 2 for homozygotes of the alternative allele); and *α* was the additive SNP effect. We used 10 PEER factors, as the diagnostic plot of the factor relevance showed a very similar pattern for the variance beyond the 10th factor (Supplementary Fig. S12). The *cis*-eQTL mapping window was defined from 1 Mb upstream/downstream of the transcription start site; all other SNP expression combinations were defined as *trans* associated. For both *cis*- and *trans*-eQTL, we applied the *P*-value threshold that corresponded to FDR < 0.01.

### Transcriptome-wide association study and Mendelian randomization analysis

We corrected gene expression data for age, genetic group, and PEER factors, and the residuals were used as the response variable in TWAS analysis. With S-PrediXcan [45], we first estimated the weights and covariance matrices of the SNPs within each gene to build a gene expression prediction model. Second, we estimated the associations between predicted gene expression levels and the traits using the GWAS summary statistics and gene expression prediction models. The identified genes were visualized using Rldeogram [46]. Subsequently, an MR analysis was done using the software MR-JTI [47] to assess causal inference between gene expression and egg production. This analysis combined LD scores, eQTL mapping results, and GWAS summary statistics to obtain candidate genes. The input SNPs were pruned such that only relatively independent variants (LD < 0.2) associated with the expression of TWAS-identified genes were used. LD scores for each independent variant were obtained with GCTA [48]. Bonferroni adjustment was applied to correct for multiple testing.

## Results

### Variance components for cumulative egg number and egg number at different stages

Estimated phenotypic variances were equivalent between models A and AD (Table 1). Phenotypic variance increased with age for cumulative egg number and increased from EN400 to EN600 for egg number at different stages. For cumulative egg number, the additive variance explained a similar proportion of the phenotypic variance at the early and late laying stage for the 2 models, 56% and 57% for CEN200, 17% and 19% for CEN300, and 13% to 14% for CEN600 and CEN700. In model AD, the dominance variance accounted for a small proportion of the total phenotypic variance at the early stages (3% for CEN200 and 6% for CEN300), a substantial proportion at the middle stages (15% for CEN400 and 23% for CEN500), and a negligible proportion at late stages, below 0.5%. For egg number at different stages, the additive variance explained a similar proportion of phenotypic variance for both models only at the early stage of the laying cycle (16% and 19% for EN300). In model AD, dominance contributed 9% to 45% of the phenotypic variance for cumulative egg number and egg number at different stages for the whole laying period.

**Table 1:** Estimates of variances for cumulative egg number and egg number at different stages

Trait	Model A		Model AD			
	$\sigma _a^2/\sigma _p^2$ (%)	$\sigma _p^2$	$\sigma _a^2/\sigma _p^2$ (%)	$\sigma _d^2/\sigma _p^2$ (%)	$\sigma _G^2/\sigma _p^2$ (%)	$\sigma _p^2$
CEN200	57	66	56	3	59	66
CEN300	19	180	17	6	23	180
CEN400	16	426	12	15	27	431
CEN500	11	1,158	7	23	29	1,188
CEN600	13	2,589	13	1e-2	13	2,589
CEN700	14	4,400	14	3e-1	14	4,401
EN300	19	104	16	9	25	104
EN400	25	93	16	31	48	96
EN500	12	315	2	45	47	331
EN600	17	547	13	14	26	552
EN700	18	554	9	23	32	560
EN300_500	13	595	8	27	35	615
EN500_700	21	1,732	15	19	34	1,749

CENX: cumulative egg number until X days of age; EN300_500: egg number between 300 and 500 days of age; EN500_700: egg number between 500 and 700 days of age; ENX: egg number in 100-day interval until X days of age; model A: model with only additive genetic effects; model AD: model with additive and dominance effects.

Standard errors for phenotypic variances ranged from 4 to 274 for model A and from 4 to 272 for model AD. Standard errors for additive variances ranged from 6 to 360 for model A and from 6 to 373 for model AD. Standard errors for dominance variances ranged from 5 to 507 for model AD.

### Genome-wide association study and candidate variants

**Figure 2:**
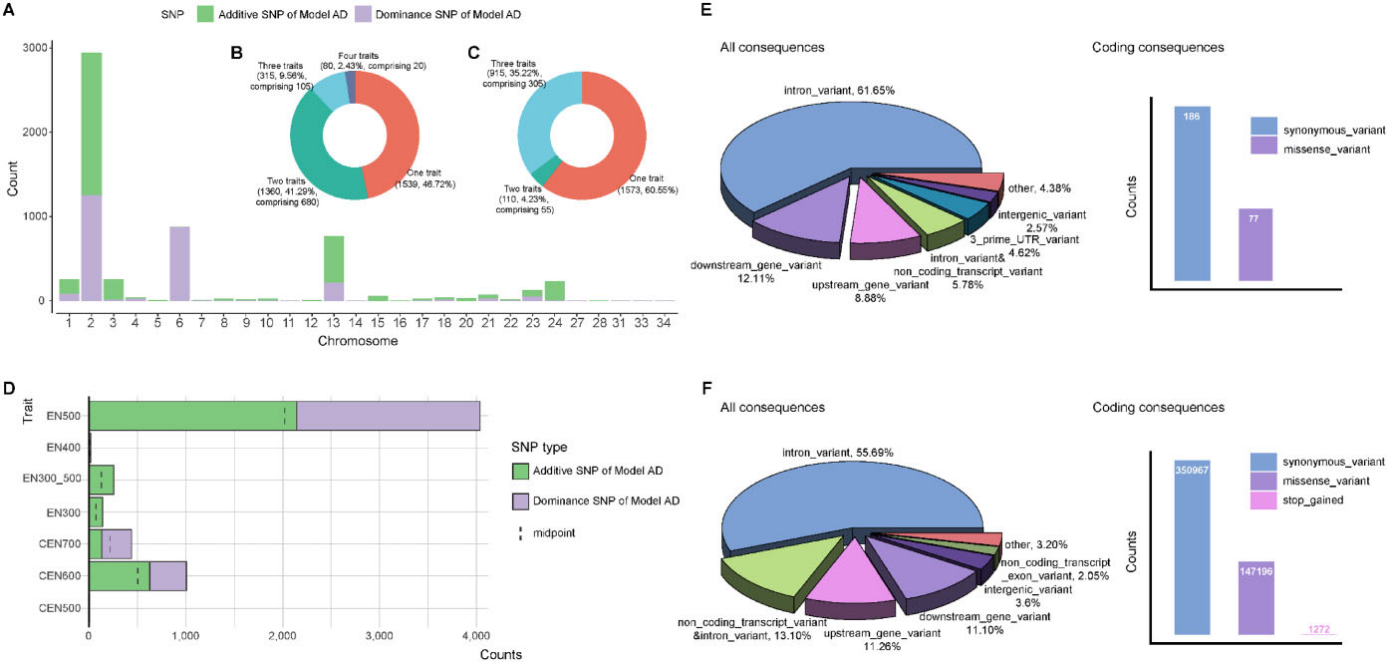
Characteristics of significant SNPs. (A) Distribution of significant SNPs across chromosomes. (B) Number and percentage of significant additive SNPs in model AD for different trait combinations. (C) Number and percentage of significant dominance SNPs in model AD for different trait combinations. (D) Distribution of significant SNPs across traits, filtered by SNP effects. (E) Variant Effect Predictor (VEP) annotations of significant SNPs. (F) VEP annotations of all SNPs.

The number of significant additive and dominance SNPs detected by model AD was 3,294 and 2,598 respectively, while no significant SNPs were detected by model A (Supplementary Table S1). The significant SNPs were mainly located on chromosomes 1, 2, 3, 6, and 13 (Fig. 2A). Among them, 805 additive SNPs and 360 dominance SNPs were shared between multiple traits (Fig. 2B, C). For each trait, more than 50% of the significant SNPs were detected as additive SNPs, except for CEN700 (Fig. 2D). For the significant SNPs, most of the annotated variants were intron variants, 61.65%, and for all SNPs, this was 55.69% (Fig. 2E, F). Among the coding variants, synonymous variants were the most abundant variants, accounting for ∼70% of both significant and all SNPs. While traditionally considered neutral, emerging evidence highlights the potential function of synonymous variants in mRNA stability and splicing [49]. The 3′ untranslated region (3′UTR) variants were over 3-fold enriched among the significant SNPs compared to all SNPs (Supplementary Table S2), which was demonstrated to play a crucial role in posttranscriptional and translational processes [50, 51].

**Figure 3:**
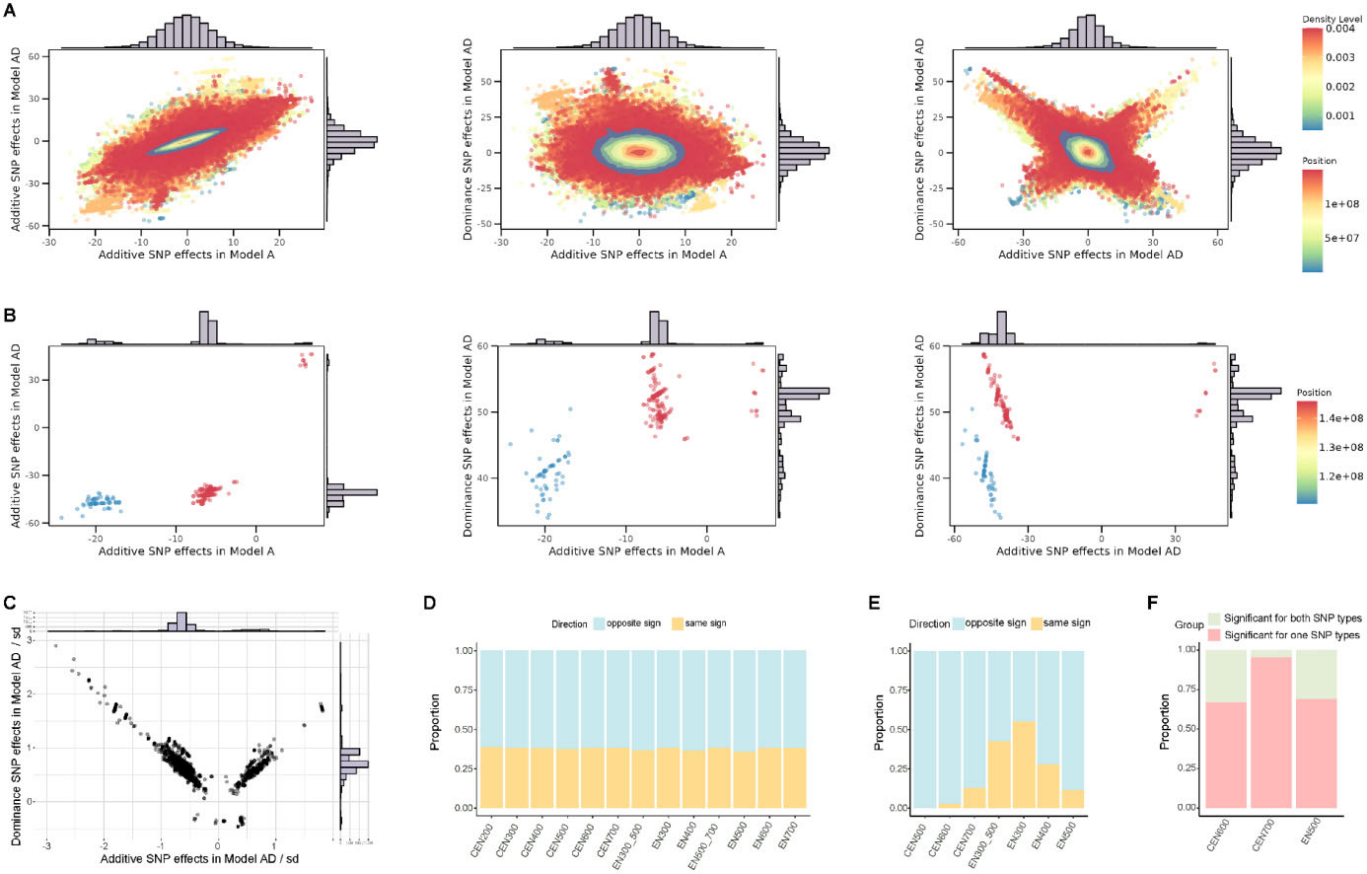
Estimated additive and dominance SNP effects from an additive model and an additive-dominance model. (A) SNP effects for all SNPs on chromosome 2 for CEN700. (B) SNP effects for significant SNPs on chromosome 2 for CEN700. (C) Distribution of SNP effects standardized using the phenotypic standard deviation for significant SNPs across traits. (D) Direction of estimated additive and dominance SNP effects for all SNPs in an additive-dominance model. (E) Direction of estimated additive and dominance SNP effects for significant SNPs in an additive-dominance model. (F) SNPs significant for both additive and dominance as a proportion of total significant SNPs.

To further explore the advantages of incorporating dominance into the GWAS model to identify the trait-related variants, we examined estimated SNP effects from the different models. The detected additive SNP effects located at chromosome 2 of CEN700 from A and AD models were clearly correlated to each other (Fig. 3A), and a subset of SNPs with extreme effects in model AD were regarded as significant (Fig. 3B). The pattern was similar for other chromosomes and traits. For significant SNPs, most of the estimated additive SNP effects were negative, while most of the estimated dominance effects were positive (Fig. 3C). The additive and dominance SNP effects were in opposite directions for approximately 60% of all SNPs (Fig. 3D), and this proportion was even higher for significant SNPs (Fig. 3E). Moreover, 251, 20, and 958 SNPs were significant for additive SNP and dominance SNP effects at the same time in traits CEN600, CEN700, and EN500 (Fig. 3F). If we trimmed the significant SNPs based on LD with *r*^2^ ≤ 0.2 via the Plink command (–indep-pairwise 50 5 0.2), a total of 132 independent genomic regions related to egg production traits were identified.

### eQTL mapping results

**Figure 4:**
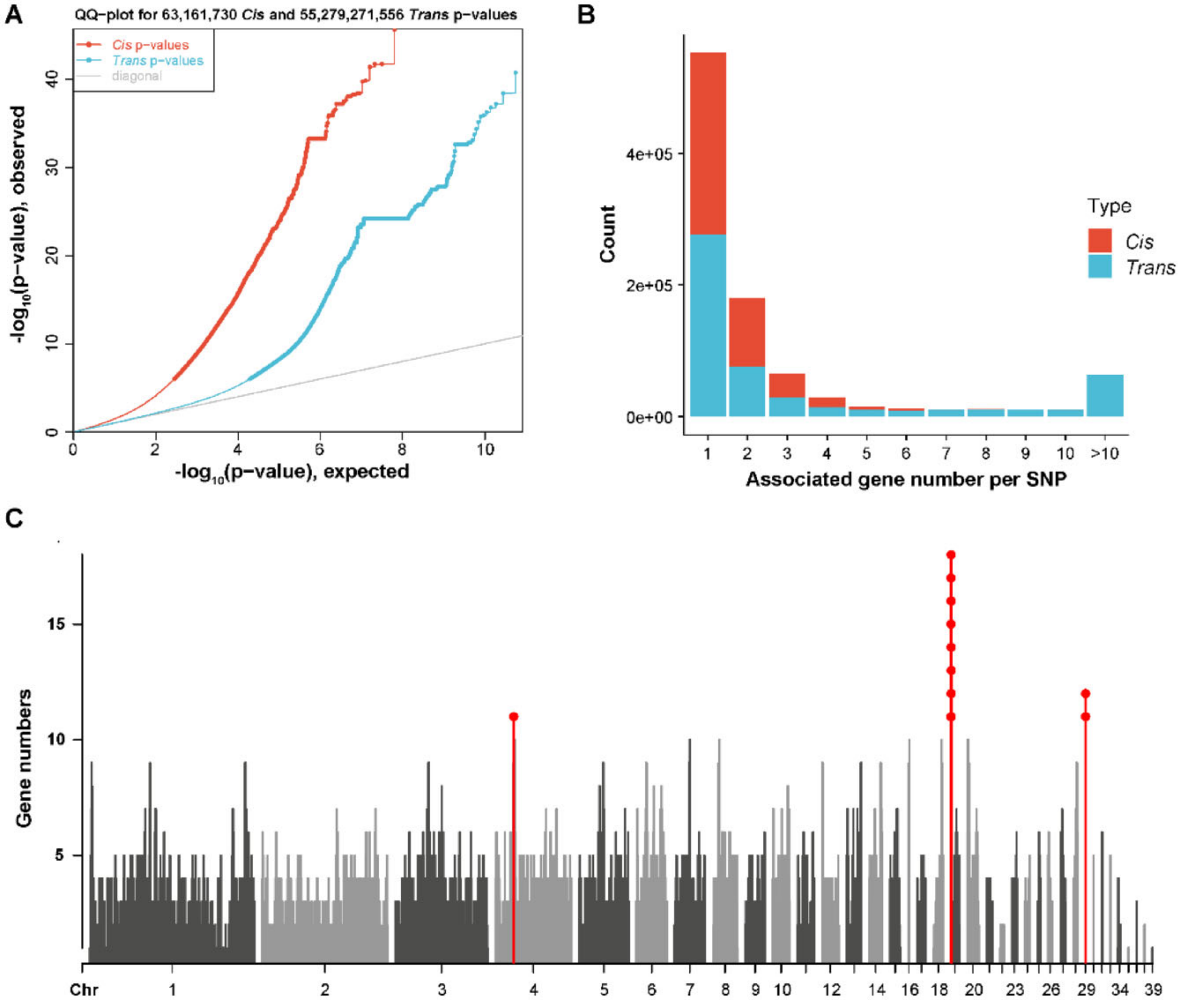
eQTL profiles based on transcriptome sequencing data of ovary tissue collected at 150, 250, 320, 500, and 700 days of age (*n* = 120). (A) Q–Q plot of −log_10_(*P*-value) of eQTL analysis. (B) Analysis of eQTL pleiotropy. The x-axis of the histogram represents the number of different gene expressions with which an SNP was associated, and the y-axis represents the eQTL count. (C) Distribution of eQTL hotspot for *cis*-eQTLs. The x-axis represents the location on the chromosomes, and the y-axis indicates the number of genes associated with each eQTL. If the number of associated genes is greater than 10, it is shown in red.

After removing genes with TPM values equal to 0 in all samples, expression data from 28,126 genes were kept for eQTL mapping analysis. The eQTL analysis assessed associations between 1,967,661 SNPs and expression of 28,126 genes, of which 63,161,730 were *cis*-SNP-expression combinations, and 55,279,271,556 were *trans*-SNP-expression combinations (Fig. 4A). At the threshold *P* < 1.12E-04 (FDR < 0.01), we identified 704,987 significant *cis*-SNP-expression combinations, corresponding to 440,374 *cis*-acting SNPs and 11,129 *cis*-eQTL-associated genes. At the threshold *P* < 3.68E-07 (FDR < 0.01), we identified 2,035,416 significant *trans*-SNP-expression combinations, corresponding to 516,993 *trans*-acting SNPs and 19,106 *trans*-eQTL-associated genes.

An eQTL can influence the expression of multiple genes, which is denoted as pleiotropy of eQTL [52]. Descriptive statistics revealed that 37.20% (163,815 of 440,374) of the *cis*-eQTL and 46.55% (240,662 of 516,993) of the *trans*-eQTL were associated with the expression of 2 or more genes, and 37 *cis*-eQTL and 63,608 *trans*-eQTL were associated with the expression of more than 10 genes (Fig. 4B). It appeared that the *cis*-eQTL that displayed pleiotropy were distributed in specific regions on chromosomes 4, 19, and 29, which could be qualified as eQTL hotspots (Fig. 4C).

### TWAS identify 298 unique genes for egg production traits

**Figure 5:**
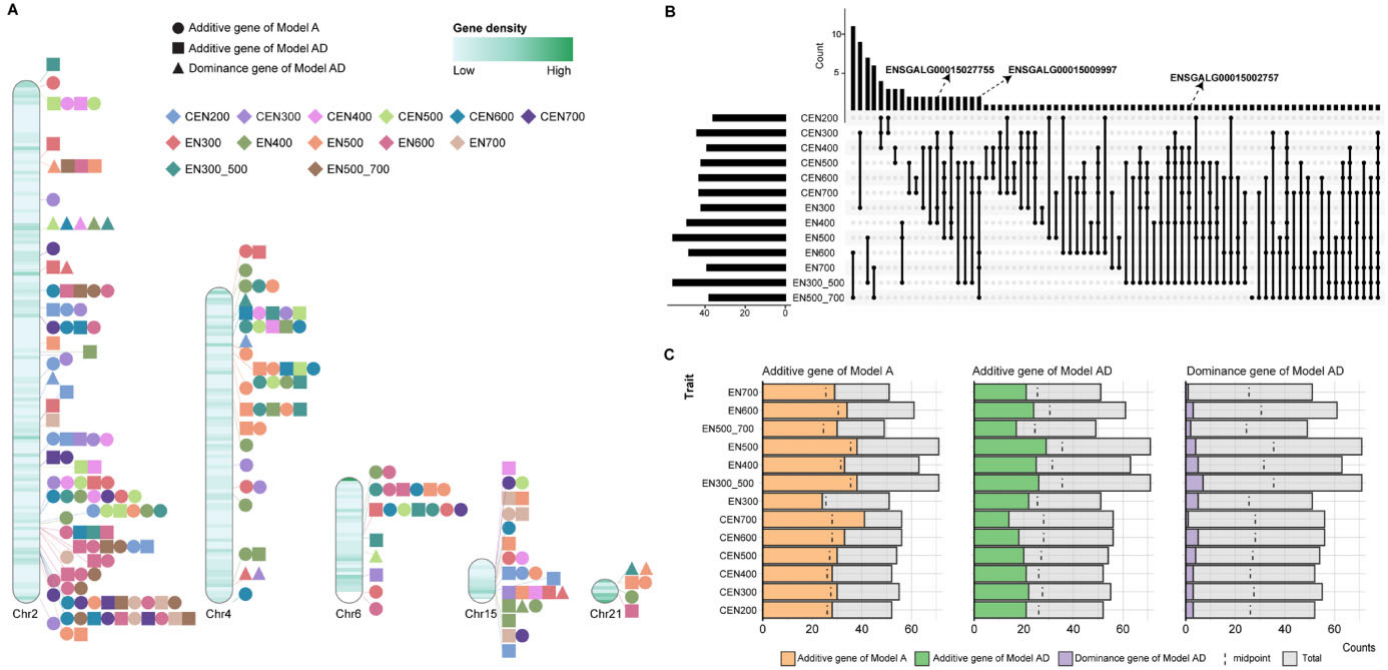
Genes affecting egg number traits identified by TWAS analysis. (A) Statistically significant gene–trait associations identified by S-predixcan. Each association is arranged according to the SNP location on each chromosome, and the points are color-coded by traits. Circle represents the additive effect from an additive model, box represents an additive effect from an additive-dominance model, and triangle represents the dominance effect from an additive-dominance model. Gene density is expressed as the average number of genes in a 1-Mb window. (B) Distribution of significant genes identified in the TWAS analysis across egg number traits. (C) Distribution of significant genes across traits, filtered by models.

We performed TWAS analysis using S-PrediXcan, revealing 742 statistically significant gene expression–egg number associations, comprising 298 genes, whose imputed expression is associated with cumulative egg number and egg number at different stages (Supplementary Table S3). Using the RIdeogram for visualization, we found several genes affecting multiple egg production traits on chromosomes 2, 4, 6, 15, and 21 (Fig. 5A) and chromosomes 1, 3, 5, 17, and 27 (Supplementary Fig. S13). Gene expression was significantly associated with multiple egg production traits, especially at the middle stage of the laying period (Fig. 5B). Across different ages, we found that *ENSGALG00015002757* influenced egg production throughout the whole laying period, affecting traits such as EN300, EN400, CEN400, CEN500, CEN600, CEN700, and EN300_500 (Fig. 5B and Supplementary Table S3). In contrast, *ENSGALG00015027755* played a role primarily in the early stage of the laying period (CEN300, CEN400, EN300, EN400; Fig. 5B and Supplementary Table S3), while *ENSGALG00015009997* was associated with the late stage of the laying period (CEN600, CEN700, EN600, EN700, and EN500_700; Fig. 5B and Supplementary Table S3). In model A, 416 significant gene–egg number combinations were found, while model AD revealed 280 significant combinations for additive effects and 46 for dominance effects (Fig. 5C and Supplementary Table S3). We further performed a MR analysis in order to test for causality using MR-JTI and to prioritize the genes identified by TWAS. We identified 125 candidate genes (Supplementary Table S4).

### Multiomics data analysis for egg production traits

**Figure 6:**
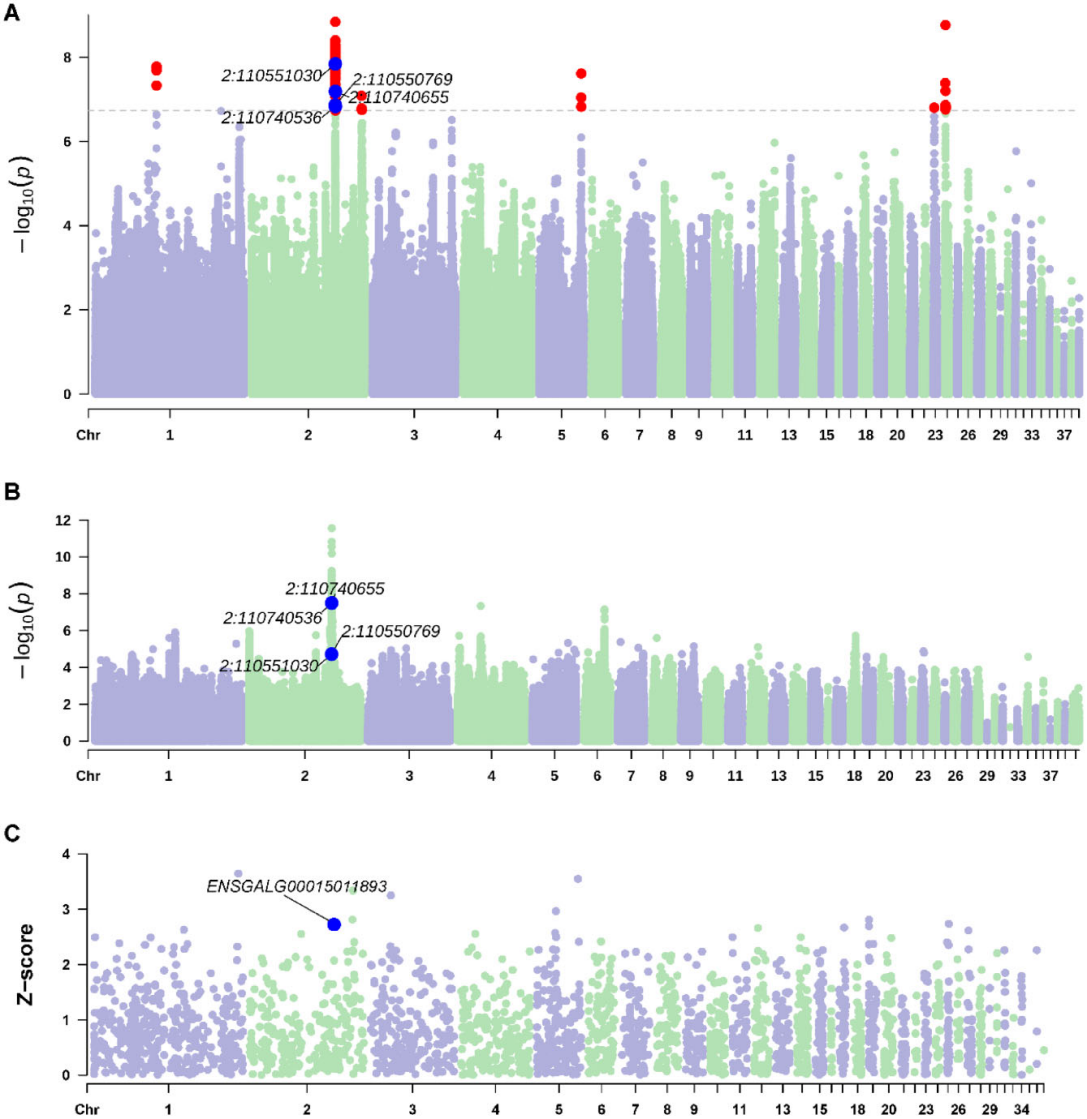
Multiomics data analysis of genetic determinants underlying cumulative egg number until 700 days of age. (A) GWAS results for trait CEN700. Each dot represents 1 SNP, and significantly associated SNPs are colored in red with a threshold FDR of 0.01. Blue dots point out candidate SNPs. (B) eQTL mapping for gene *ENSGALG00015011893*. Each dot represents 1 SNP. Blue dot indicates candidate SNPs. (C) TWAS results for trait CEN700. Each dot represents 1 gene. Blue dot indicates candidate gene.

Significant SNPs detected in the GWAS results (Table 2, Fig. 6A, and Supplementary Figs. S14–S16A) were associated with 27 genes in *cis*-SNP-gene associations identified in the eQTL analysis (Table 2, Fig. 6B, Supplementary Figs. S14–S16B, and Table S5), among which 4 genes were also detected by TWAS: *ENSGALG00015011893, ENSGALG00015011943, ENSGALG00015026475*, and *ENSGALG00015025721* (Table 2, Fig. 6C, and Supplementary Figs. S14–S16C). Further causal inference with MR analysis showed that the 4 genes were potential causal genes for egg production (Table 2 and Supplementary Table S4). *ENSGALG00015011893* is located on chromosome 2, and the expression level was significantly associated with SNPs 2:110550769, 2:110551030, 2:110740536, and 2:110740655 (*P*-values ranged from 3.18E-08 to 1.91E-05; Table 2 and Fig. 6B) and was associated with CEN700 with a *z*-score of −2.72 (corresponding *P* = 6.46E-03; Table 2, Fig. 6C, and Supplementary Table S3). The expression level of *ENSGALG00015011943* was associated with SNP 2:110551030 of trait CEN500 (*P* = 9.86E-06; Table 2 and Supplementary Fig. S14B) and was associated with CEN500 with a *z*-score of −2.99 (corresponding *P* = 2.76E-03; Table 2, Supplementary Fig. S14C, and Supplementary Table S3). Similarly, the expression level of *ENSGALG00015026475* was associated with 5 SNPs locating on chromosome 15 (*P*-values ranged from 1.09E-07 to 1.15E-07; Table 2, and Supplementary Fig. S15B) and was associated with EN500 with a *z*-score of −2.80 (corresponding *P =* 5.04E-03; Table 2, Supplementary Fig. S15C and Supplementary Table S3). The expression level of *ENSGALG00015025721* was associated with 6 SNPs locating on chromosome 21 (*P*-values ranged from 4.32E-07 to 2.54E-06; Table 2 and Supplementary Fig. S16B) and was associated with EN500 with a *z*-score of 2.91 (corresponding *P =* 3.63E-03; Table 2, Supplementary Fig. S16C, and Supplementary  Table S3).

**Table 2:** Genes associated with egg production traits in an additive-dominance model for additive SNP effects

Ensembl ID	SNP	SNP_type	Trait	GWAS_pvalue	eQTL_pvalue	*z*-score	MR_beta	MR_95%_CI
*ENSGALG00015011893 (TMEM68-like)*	2:110550769	Additive SNP effects	CEN700	1.37E-07	1.91E-05	−2.72	−0.50	(−0.51, −0.11)
	2:110551030	Additive SNP effects	CEN700	1.44E-08	1.91E-05			
	2:110740536	Additive SNP effects	CEN700	1.47E-07	3.18E-08			
	2:110740655	Additive SNP effects	CEN700	6.50E-08	3.18E-08			
*ENSGALG00015011943 (TGS1)*	2:110551030	Additive SNP effects	CEN500	2.48E-09	9.86E-06	−2.99	−0.47	(−0.46, 0.05)
*ENSGALG00015026475 (DNAH10)*	15:4509433	Additive SNP effects	EN500	1.11E-07	1.31E-06	−2.80	−0.38	(−0.58, −0.16)
	15:4509450	Additive SNP effects	EN500	1.14E-07	1.31E-06			
	15:4509495	Additive SNP effects	EN500	1.09E-07	1.31E-06			
	15:4510674	Additive SNP effects	EN500	1.15E-07	1.31E-06			
	15:4512681	Additive SNP effects	EN500	1.13E-07	1.94E-05			
*ENSGALG00015025721 (CEP104)*	21:876167	Dominance SNP effects	EN500	2.54E-06	3.46E-10	2.91	0.38	(0.01, 0.68)
	21:939370	Dominance SNP effects	EN500	7.47E-07	2.04E-11			
	21:940575	Dominance SNP effects	EN500	7.47E-07	2.04E-11			
	21:959428	Dominance SNP effects	EN500	4.32E-07	2.04E-11			
	21:960286	Dominance SNP effects	EN500	7.47E-07	2.04E-11			
	21:960523	Dominance SNP effects	EN500	1.68E-06	3.62E-11			

**Figure 7:**
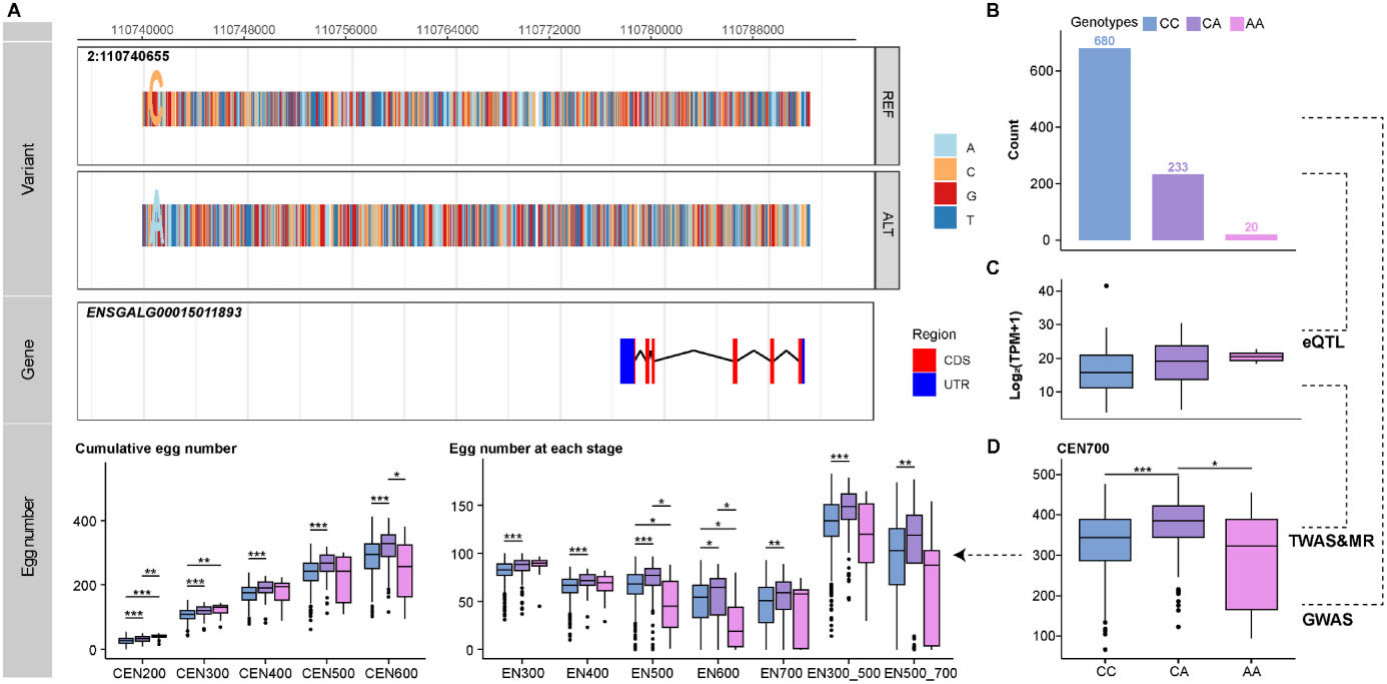
The associations between candidate genetic variant 2:110740655 and gene expression (*ENSGALG00015011893*) and egg number. (A) Schematic representation of the relationship among genetic variant, gene expression, and egg number. The top panel depicts the genomic region from 110.74 to 110.79 Mb, with the highlighted letter indicating SNP 2:110740655. The middle panel illustrates the transcript structure for gene *ENSGALG0015011893*, and the bottom panel shows phenotypic changes in egg number across genotypes for SNP 2:110740655 (*P < 0.05, **P < 0.01, ***P < 0.001). (B) Counts of different genotypes for SNP 2:110740655. (C) Expression level of gene *ENSGALG0015011893* across genotypes for SNP 2:110740655. (D) CEN700 across genotypes for SNP 2:110740655 (*P < 0.05, ***P < 0.001).

In addition to candidate genes, we also pinpointed several candidate SNPs, though they are not the leading SNPs in GWAS results, suggesting the leading SNPs may not function as expression quantitative trait locus but instead influence egg number through other mechanisms. For SNP 2:110740655, reference allele C and alternative allele A, cumulative egg number and egg number at different stages of genotype CA were higher than genotype CC, and the expression of *ENSGALG00015011893* for genotype AA was the highest (Fig. 7). For SNP 2:110551030, the egg number of genotype GG at different stages was smaller than the genotype GA (Supplementary Fig. S17A), and the expression of *ENSGALG00015011943* for genotype GG was lower than the genotype GA (Supplementary Fig. S17B). For SNP 15:450945, the egg number of genotype GG at different stages was smaller than the genotype GA (Supplementary Fig. S17C), and the expression of *ENSGALG00015026475* for genotype GG was lower than the genotype GA (Supplementary Fig. S17D). For SNP 21:940575, the egg number of genotype AA at different stages was smaller than the genotype GA (Supplementary Fig. S17E), and the expression of *ENSGALG00015025721* for genotype AA was the highest (Supplementary Fig. S17F).

### Implications for heterosis

**Figure 8:**
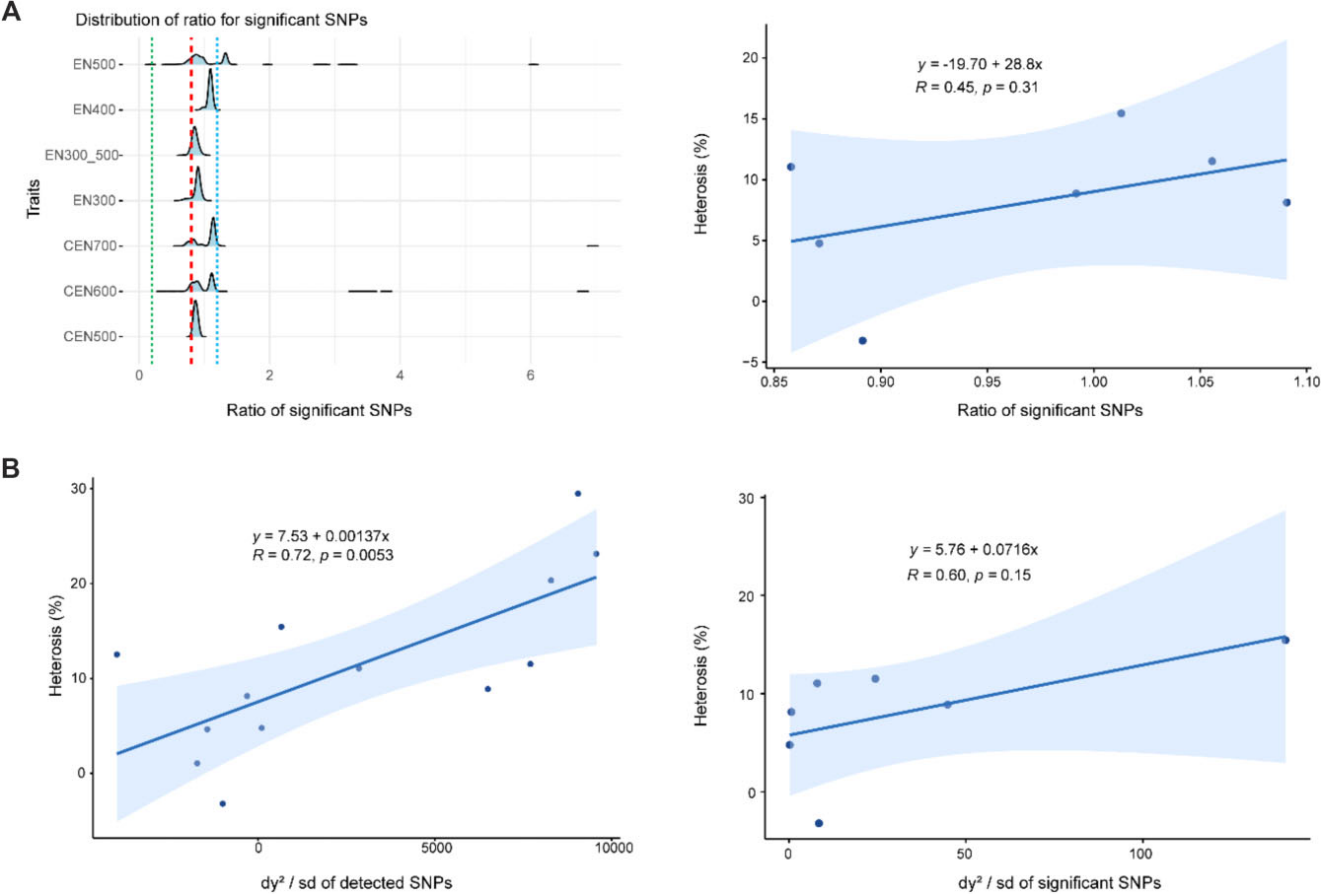
Implications for heterosis of egg production traits. (A) Distribution of ratios for significant SNPs (green dash line: *r* = 0.2, red dash line: *r* = 0.8, blue dash line: *r* = 1.2) and correlation between ratio and heterosis. *r* is the SNP ratio, $| {\frac{{{t_{Dom}}}}{{{t_{Add}}}}} |$, calculated based on the *t*-statistics from model AD. (B) Correlations between the sum of dy^2^/sd of all SNPs (left panel) or only the significant SNPs (right panel) and heterosis. Here, *d* represents dominance effects, *y* represents the squared difference in allele frequency, and *sd* represents the phenotypic standard deviation.

Observed heterosis from the predicted mean phenotypes of each genetic group increased across ages for cumulative egg number from 1.04% to 11.51%, except for CEN200. For egg number at different stages, heterosis increased from −3.22% to 29.48% (Table 3). Leveraging model AD enables the calculation of SNP ratios to assess the relative importance of dominance compared to additive SNP effects. The ratios for most of the significant SNPs were higher than 0.8 (Fig. 8A, red dash line) and smaller than 1.2 (Fig. 8A, blue dash line), suggesting complete dominance. Across traits, we observed a positive correlation coefficient of 0.45 (*P* = 0.31) between SNP ratios and heterosis (Fig. 8A). Similarly, a positive and significant correlation of 0.72 was observed between the sum of dy^2^ across all SNPs and heterosis, with *P* = 0.0053 (Fig. 8B). Finally, a positive but insignificant correlation of 0.60 was observed for significant SNPs between the sum of dy^2^ and the heterosis, with *P* = 0.15 (Fig. 8B).

**Table 3:** Predicted egg production traits for each genetic group and estimates of heterosis for the crossbreds

Trait	WW	YY	WY	YW	Heterosis (%)
CEN200	38.06	14.67	28.604	14.87	12.52
CEN300	126.10	93.37	110.72	93.37	1.04
CEN400	198.80	143.47	178.49	143.5	4.63
CEN500	269.60	194.08	247.46	194.02	4.77
CEN600	320.30	229.35	304.43	229.21	8.87
CEN700	378.20	251.50	363.34	251	11.51
EN300	88.81	77.54	82.656	77.39	−3.22
EN400	71.25	50.59	66.927	50.54	8.13
EN500	68.68	47.72	68.4657	47.69	15.44
EN600	48.77	33.54	56.161	33.08	20.33
EN700	56.36	21.01	57.0768	20.59	29.48
EN300_500	143.50	99.93	136.089	99.92	11.05
EN500_700	104.80	54.36	113.232	53.28	23.13

CENX: cumulative egg number until X days of age; ENX: egg number in 100-day interval until X days of age; EN300_500: egg number between 300 and 500 days of age; EN500_700: egg number between 500 and 700 days of age.

Heterosis (%): the average percentage of performance of crossbreds being better than the average performance of the 2 parental lines.

## Discussion

In the current study, by incorporating dominance SNP effects into the GWAS model and using whole-genome sequencing data for a complete double-crossed hybrid population of 1,004 chickens, we successfully identified genetic variants related to egg production traits. Egg production remains the most important trait for laying hens, despite the recent expansion of breeding goals to include health and welfare-related traits [53]. In total, the eQTL mapping analysis identified 704,987 *cis*-SNP-expression combinations and 2,035,416 *trans*-SNP-expression combinations, and TWAS analysis identified 298 genes. By carrying out the multiomics data analysis, 4 novel candidate genes for egg production traits were identified. Moreover, observed heterosis positively related to the heterosis predicted based on the estimated dominance SNP effects and allele frequencies, as well as average ratios of dominance to additive effects.

The traditional GWAS analysis in livestock normally focuses on estimating additive effects, while nonadditive effects, such as dominance, are frequently overlooked. When nonadditive effects actually exist but are not modeled, they may end up partly in the residual effect and partly in the additive effect, leading to bias in the estimation of additive effects [54]. Additionally, incorporating dominance SNP effects is justified by the fact that a substantial portion of genetic variance, especially in mixed populations of purebred and crossbred animals, is likely explained by nonadditive effects [55]. In the current study, with only additive SNP effects in model A, no significant SNPs were found, while by adding dominance SNP effects in model AD, several signals appeared across traits (Supplementary Figs. S18–S21). This finding agrees with previous research that emphasizes the role of including dominance effects in the model for enhancing the detection of associations [15, 56], particularly when using crossbred data [9]. To further explore the advantages of incorporating dominance into the GWAS model, we analyzed SNP effects in both models. Additive SNP effects were strongly correlated between the models, indicating that additive SNP effects remained largely unchanged in both models, while significant SNPs exhibited more extreme effects in model AD. In model A, we suspected that opposing additive and dominance SNP effects can potentially cancel each other out, as allele substitution effects, as estimated in model A, are defined as $a + (q - p)d$, where *a* is the additive effect, *d* is the dominance effect, and *q* and *p* are the allele frequencies [18]. This was supported by Fig. 3B, which showed that significant SNPs on chromosome 2 of CEN700 exhibited negative additive effects and positive dominance effects, appearing to offset each other. Model AD distinguished between additive and dominance SNP effects, leading to the identification of more variants in our population, which consists of purebred and crossbred animals. However, this finding cannot be generalized and will depend upon if traits are affected by dominance. For example, a study in Large White pigs reported different results across traits, with substantial dominance SNP effects observed for age at 100 kg, while no significant dominance SNP effects were detected for backfat thickness at 100 kg [57]. Our findings highlight the importance of including dominance effects in GWAS to improve the accuracy and power to detect egg production–related variants, especially in studies involving crossbred animals.

In the current study we identified a large number of trait-related variants, possibly due to the use of whole-genome sequencing data. Most GWAS studies in chickens have relied on genotype data obtained from SNP chips, which include only a fraction of all the variants segregating in the whole chicken genome. In contrast, whole-genome sequencing data encompass nearly all genomic variants, which can enhance the effectiveness of GWAS in identifying causal mutations for quantitative traits [58]. Taken together, incorporating dominance into the GWAS model proved beneficial for identifying genetic variants for egg production traits of layers in our full diallel cross with purebred and crossbred animals with whole-genome sequencing data.

We used the reference genome of the WW line (White Leghorn), a breed known for high egg production, and the number of alleles for the alternative allele was counted (coded as 0, 1 or 2). Given this coding and the superiority of WW over YY, we expected a higher frequency of the reference allele in WW and a higher frequency of the alternative allele in YY, and therefore this coding may lead to more negative than positive additive SNP effects. Additionally, in our previous study [22], we found substantial heterosis for crossbreds, which could result from the positive dominance effects. The negative correlation observed for additive and dominance SNP effects in model AD may change or even disappear if a different allele coding would have been applied. In other words, the general relationship between the 2 SNP effects is that higher additive SNP effects correspond to higher absolute dominance SNP effects. This can be explained by the phenomenon in which the heterozygote for the significant SNPs exhibits a phenotype that is similar to the phenotype of the best homozygote (Supplementary Fig. S22). This indicates that the presence of a specific allele, whether in a heterozygous or homozygous state, results in a similar phenotype (i.e., complete dominance), leading to Fig. 3C, where absolute values of estimated additive and dominance have equal size. These findings underscore the importance of carefully considering the coding of alleles when interpreting estimated additive effects.

Incorporating dominance effects into the GWAS model also provides insights into the relative importance of SNP effects through the ratio of dominance to additive SNP effects. By integrating the SNP ratio and estimating significant *cis*-eQTLs and *trans*-eQTLs, we showed genes associated with SNPs filtered by gene action (additive, partial dominance, complete dominance, and over-dominance; Supplementary Figs. S23 and S24). We observed comparable proportions of *cis*-eQTLs and *trans*-eQTLs for additive, partial-dominance, complete-dominance, and over-dominance gene actions, even after pruning SNPs based on LD. This indicated that *cis*-acting and *trans*-acting contribute equally to SNPs of additive, partial-dominance, complete-dominance, and over-dominance gene action. In contrast, Cui et al. [9] found that genes associated with SNPs of additive gene action are mainly *cis*-acting, and genes associated with SNPs of dominant gene action are mainly *trans*-acting. The higher SNP density in the current study, along with differences in the methods used to estimate additive and dominance SNP effects, may explain the observed discrepancies.

In the current study, integrating GWAS, eQTL mapping analysis, and TWAS, we ultimately discovered 4 novel genes with potential causal roles in influencing egg production. *ENSGALG00015011893* is a novel gene potentially mapped to *LOC421125* in the NCBI dataset and annotated as *TMEM68-like* (transmembrane protein 68-like). *TMEM68* has been identified as an enzyme involved in lipid metabolism [59], which may be crucial for the formation of yolk. *ENSGALG00015011943*, annotated as *TGS1* (trimethylguanosine synthase 1), was also reported as a differential expressed gene in pre-recruitment and pre-ovulatory follicles [60], indicating a potential role in regulating the egg-producing process. Notably, SNP 2:110551030 was associated with the expression of both genes for cumulative egg number until different ages, illustrating its pleiotropic effects. Despite the absence of direct regulatory annotation in our reference genome, the significant eQTL association between this SNP and both genes supports the hypothesis that the variant may have a regulatory function or serve as proxy for causal variants within regulatory elements. This integrated analysis of genomic and transcriptome data helps to identify trait-related genetic variants and genes, showing that multiomics data can contribute to deciphering genetic mechanisms underlying egg production by establishing connections between genetic variants, gene expression, and egg number.

In addition to identifying candidate genetic variants related to egg production traits, we also explored implications regarding heterosis utilizing estimated dominance SNP effects, given that chicken is one of the most well-known animals in which hybrid vigor, or heterosis, is leveraged in commercial populations. Following the methods described previously [9, 22], we calculated observed heterosis from predicted means for genetic groups of egg production traits and assessed the extent of dominance effects per SNP through SNP ratios. Ratios of significant SNPs identified by GWAS generally ranged from 0.8 to 1.2, with the mean value of 1.00 (Supplementary Table S6), suggesting a complete-dominance gene action [9]. Based on these results, complete dominance is expected to be the predominant pattern in egg production heterosis. We did not find other studies in animals attempt to investigate this, but this observation aligns with previous reports in plants [61, 62]. It should be noted that in our study, the observed correlations between SNP ratio and heterosis could be affected by the fact that the traits and thus also the significant SNPs across traits are highly related, or by the limited number of traits considered. Nevertheless, we did observe a positive correlation between SNP ratio and heterosis, consistent with the theory and the expectation, stating that with the increasing extent of dominance compared to additive effects, a larger amount of heterosis is expected. Moreover, we observed a lower correlation between heterosis and the SNP ratios for all compared to only the significantly detected SNPs (Supplementary Fig. S25).

In addition to the SNP ratios, we considered the quantitative genetics theory that the amount of heterosis depends on dy^2^ (i.e., the product of the dominance effects multiplied by the squared difference in allele frequency) [18]. We observed a positive and significant correlation between the sum of dy^2^ and heterosis for all SNPs, but an insignificant correlation for significant SNPs. Given the similar correlation of 0.72 for all SNPs and 0.60 for significant SNPs, we argue that the limited number of significant SNPs still had an important impact on phenotypes. In line with this, Amuzu-Aweh et al. [63] reported an accuracy of ∼0.5 for predicting heterosis using the genome-wide squared difference in allele frequency between parental pure lines. Apart from the implications to heterosis, we also observed differences between reciprocal crosses for egg production traits and also in other egg-laying traits [22]. However, we could not unambiguously determine a cause of these reciprocal differences due to the confounding effects between sex-linked genes and genetic group in our dataset. Other plausible explanations for differences between the reciprocal crosses include parent-of-origin effects or different breed origin of the mitochondrial DNA [64, 65].

## Conclusion

In the current study, we identified 5,982 SNPs and 3 candidate genes for egg production traits by analyzing 1,004 fully sequenced layers. These results suggest that incorporating dominance into the GWAS model not only helps to detect the variants for egg production traits of layers in a mixed population with purebred and crossbred animals but also demonstrates that traits with higher heterosis tended to be more affected by genes with a dominant mode of action. Moreover, multiomics data allow the contribution to deciphering genetic mechanisms underlying egg production by establishing connections between genetic variants, gene expression, and egg number.

## Availability of Source Code and Requirements

Project name: Identifying candidate genetic variants for egg number by analyzing over 1,000 fully sequenced layer project

Project homepage: https://github.com/aixin951/EP_WGS_1004_layers.

Operating system(s): Platform independent

Programming language: R

Other requirements: R 4.2.3 or higher

License: MIT

Software Heritage PID: swh:1:snp:abd33c288a589ee4a1aecd5f12238479c6c98117

## Supplementary Material

giaf064_Supplemental_Files

giaf064_Authors_Response_To_Reviewer_Comments_original_submission

giaf064_GIGA-D-24-00467_original_submission

giaf064_GIGA-D-24-00467_Revision_1

giaf064_Reviewer_1_Report_Original_submission Hua Li -- 12/23/2024

giaf064_Reviewer_2_Report_Original_submission Zhenfang Wu -- 12/29/2024

## Data Availability

The genomic and transcriptomic sequence data generated in this study are available under the following BioProject accessions: PRJCA032894 in the National Genomics Data Center (NGDC) database and PRJEB82328 and PRJEB88001 in the European Nucleotide Archive (ENA) database. All additional supporting data are available in the *GigaScience* repository, GigaDB [66]. The software code is available in SoftwareHeritage [67].

## Additional Files

**Supplementary Fig. S1**. Mapping quality of 1,004 whole-genome sequenced animals.

**Supplementary Fig. S2**. Scatterplot for pedigree and genome coefficients for crossbreds.

**Supplementary Fig. S3**. Scatterplot for pedigree and genome coefficients for crossbreds after removing conflict animals.

**Supplementary Fig. S4**. Scatterplot for pedigree and genome coefficients for WW.

**Supplementary Fig. S5**. Scatterplot for pedigree and genome coefficients for WW after removing conflict animals.

**Supplementary Fig. S6**. Scatterplot for pedigree and genome coefficients for YY.

**Supplementary Fig. S7**. Scatterplot for pedigree and genome coefficients for YY after removing conflict animals.

**Supplementary Fig. S8**. PCA plot for the 4 genetic groups.

**Supplementary Fig. S9**. Mapping quality of transcriptome data.

**Supplementary Fig. S10**. Distribution of concordance for individual and SNPs for different imputation methods.

**Supplementary Fig. S11**. Distribution of concordance for SNPs when applying missing genotype smaller than 10%.

**Supplementary Fig. S12**. Diagnostic plot of the factor relevance (automatic relevance determination parameters).

**Supplementary Fig. S13**. Genes identified by TWAS analysis.

**Supplementary Fig. S14**. Multiomics data analysis of genetic determinants underlying cumulative egg number until 500 days of age for additive SNP effects.

**Supplementary Fig. S15**. Multiomics data analysis of genetic determinants underlying egg number between 400 and 500 days of age for additive SNP effects.

**Supplementary Fig. S16**. Multiomics data analysis of genetic determinants underlying egg number between 400 and 500 days of age for dominance SNP effects.

**Supplementary Fig. S17**. The correlations between candidate variants and phenotype, as well as expression of candidate genes.

**Supplementary Fig. S18**. Q−Q plots across traits.

**Supplementary Fig. S19**. Manhattan plots of additive SNP effects for cumulative egg number and egg number at different stages in model A.

**Supplementary Fig. S20**. Manhattan plots of additive SNP effects for cumulative egg number and egg number at different stages in model AD.

**Supplementary Fig. S21**. Manhattan plots of dominance SNP effects for cumulative egg number and egg number at different stages in model AD.

**Supplementary Fig. S22**. Proportion of significant SNPs where heterozygotes exhibit a phenotype similar to the phenotype of the best homozygote.

**Supplementary Fig. S23**. *Cis-* and *trans*-acting eQTLs and their associated genes.

**Supplementary Fig. S24**. *Cis-* and *trans*-acting eQTLs and their associated genes after pruning with linkage equilibrium.

**Supplementary Fig. S25**. Correlation between SNP ratios of all SNPs and heterosis.

**Supplementary Table S1**. Significant SNPs identified by GWAS.

**Supplementary Table S2**. Annotation of significant and all SNPs.

**Supplementary Table S3**. Significant genes identified by TWAS.

**Supplementary Table S4**. MR analyzed for candidate genes identified by TWAS.

**Supplementary Table S5**. Expression level of 27 genes associated with significant SNPs identified by GWAS.

**Supplementary Table S6**. Ratio values of significant SNPs.

## Abbreviations

3′UTR: 3′ untranslated region; CEN200: cumulative egg number until 200 days of age; CEN300: cumulative egg number until 300 days of age; CEN400: cumulative egg number until 400 days of age; CEN500: cumulative egg number until 500 days of age; CEN600: cumulative egg number until 600 days of age; CEN700: cumulative egg number until 700 days of age; CEP104: centrosomal protein 104; DNAH10: dynein axonemal heavy chain 10; dy2: the sum of the dominance effects multiplied by the squared difference in allele frequency between the parental lines; EN300: egg number from 200 to 300 days of age; EN300_500: egg number from 300 to 500 days of age; EN400: egg number from 300 to 400 days of age; EN500: egg number from 400 to 500 days of age; EN500_700: egg number from 500 to 700 days of age; EN600: egg number from 500 to 600 days of age; EN700: egg number from 600 to 700 days of age; eQTL: expression quantitative trait loci; FDR: false discovery rate; GWAS: genome-wide association study; LD: linkage disequilibrium; Mb: megabase; model A: additive model; model AD: additive-dominance model; MR: Mendelian randomization; QTL: quantitative trait loci; SNP: single-nucleotide polymorphism; TGS1: trimethylguanosine synthase 1; TMEM68: transmembrane protein 68; TPM: transcripts per kilobase per million mapped reads; TWAS: transcriptome-wide association study; VEP: variant effect predictor; WW: White Leghorn chickens; WY: offspring of a cross between White Leghorn as the sire line and Beijing-You as the dam line; YW: offspring of a cross between Beijing-You as the sire line and White Leghorn as the dam line; YY: Beijing-You chickens.
