# Supplementary material for: Identifying candidate genetic variants for egg number by analyzing over 1,000 fully sequenced layers
Source: Gigascience. 2025 Jun 17;14:giaf064. doi: 10.1093/gigascience/giaf064 (PMC12203006; doi:10.1093/gigascience/giaf064)
Supplement: giaf064_Supplemental_Files [file giaf064_supplemental_files.zip › Additional_file_AN_20250307.docx]

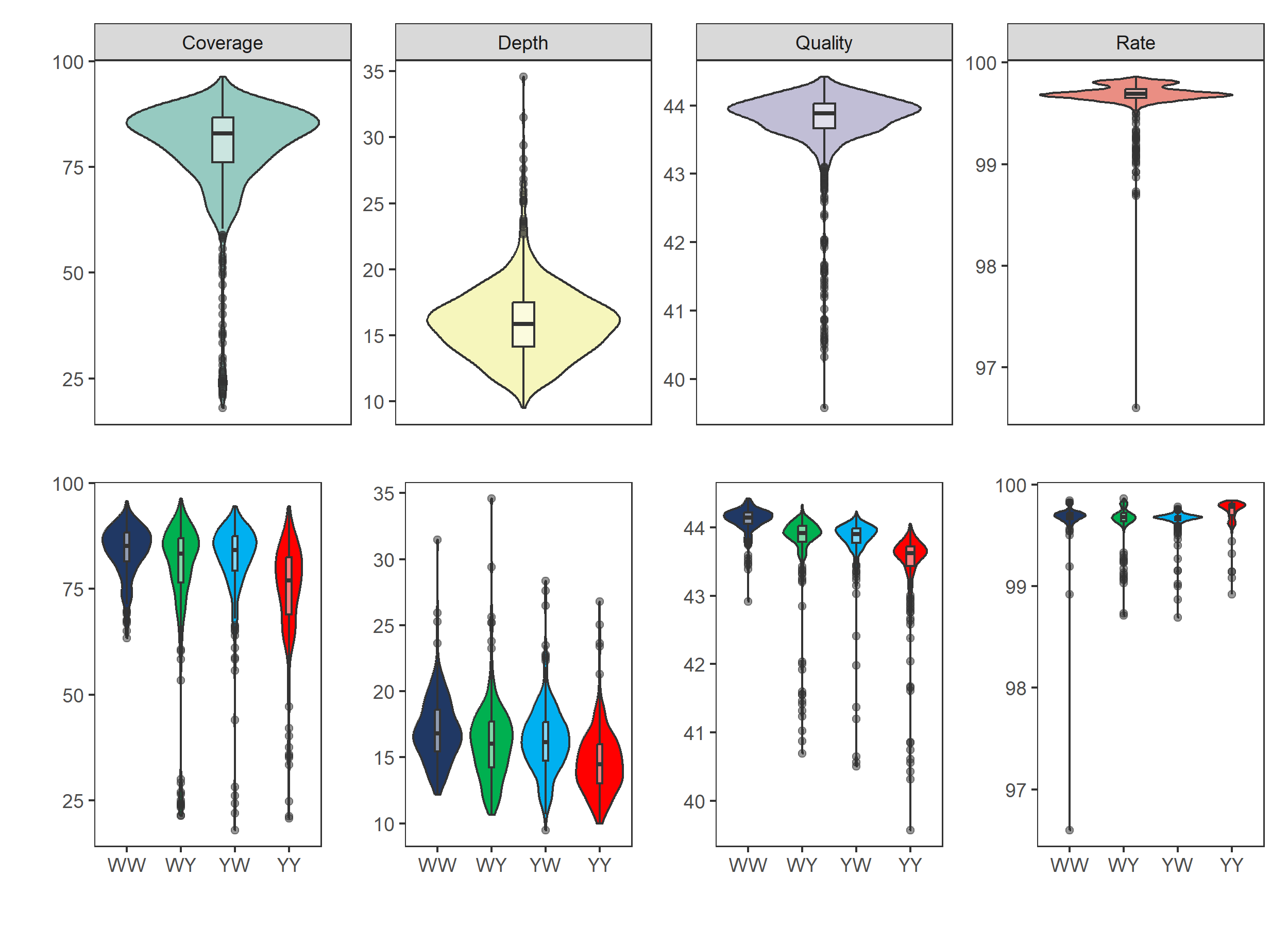


**Fig. S1.** Mapping quality of 1,004 whole-genome sequenced animals.

## Pedigree and genome inconsistencies


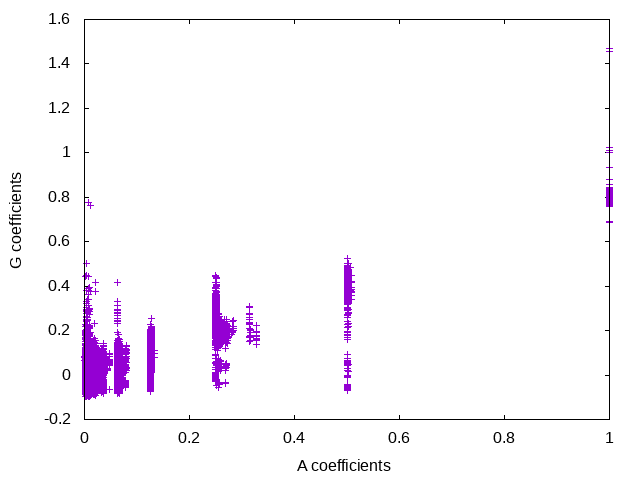
We removed conflict animals manually by calculating the pedigree-based relationship and genomic based relationship coefficients. After inconsistencies check, we removed 53 animals for the dataset, 15 for crossbreds, 8 for WW, and 30 for YY. Here are the initial and subsequent scatter plot for the relationships between pedigree and genomic.


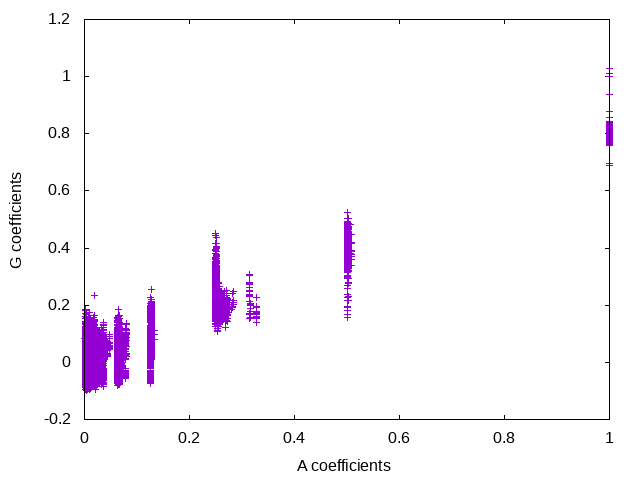
**Fig. S2.** Scatter plot for pedigree and genome coefficients for crossbreds

**Fig. S3.** Scatter plot for pedigree and genome coefficients for crossbreds after removing conflict animals


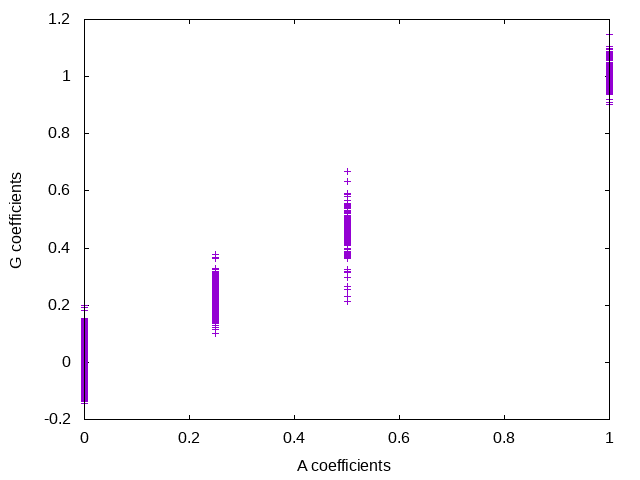

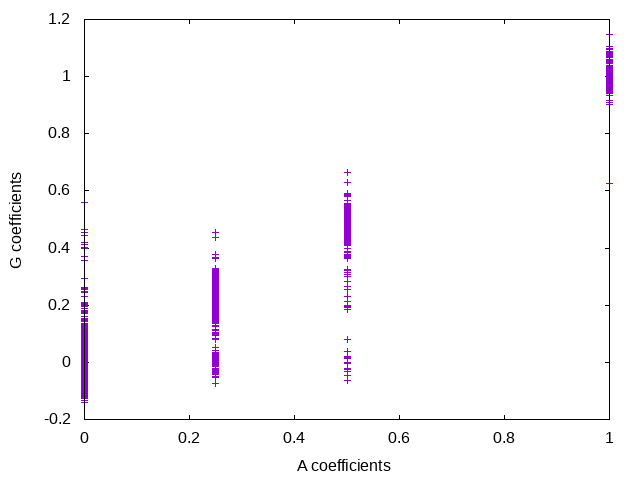
**Fig. S4.** Scatter plot for pedigree and genome coefficients for WW

**Fig. S5.** Scatter plot for pedigree and genome coefficients for WW after removing conflict animals


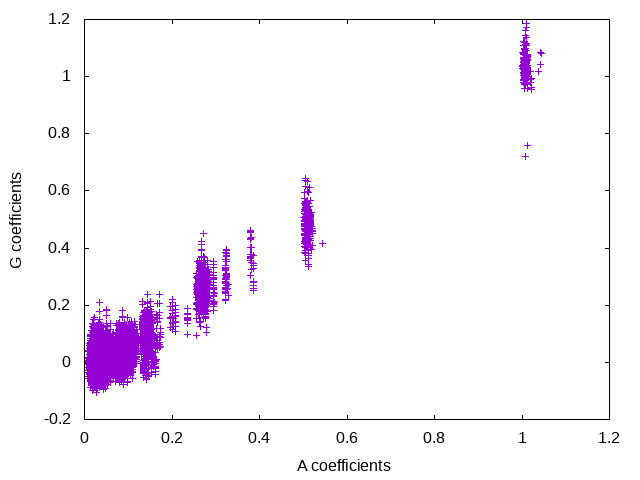

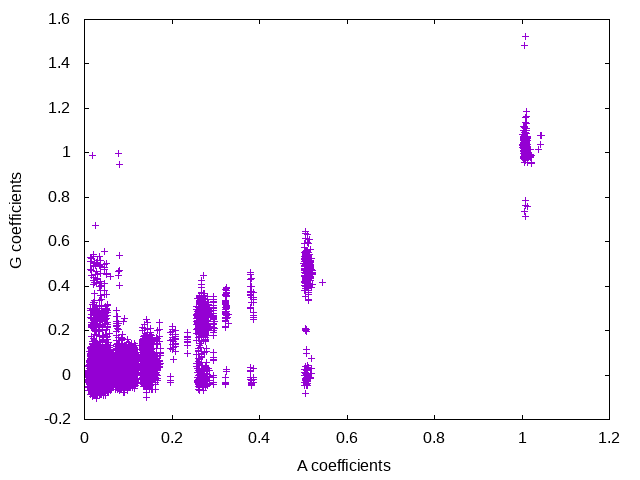
**Fig. S6.** Scatter plot for pedigree and genome coefficients for YY

**Fig. S7.** Scatter plot for pedigree and genome coefficients for YY after removing conflict animals


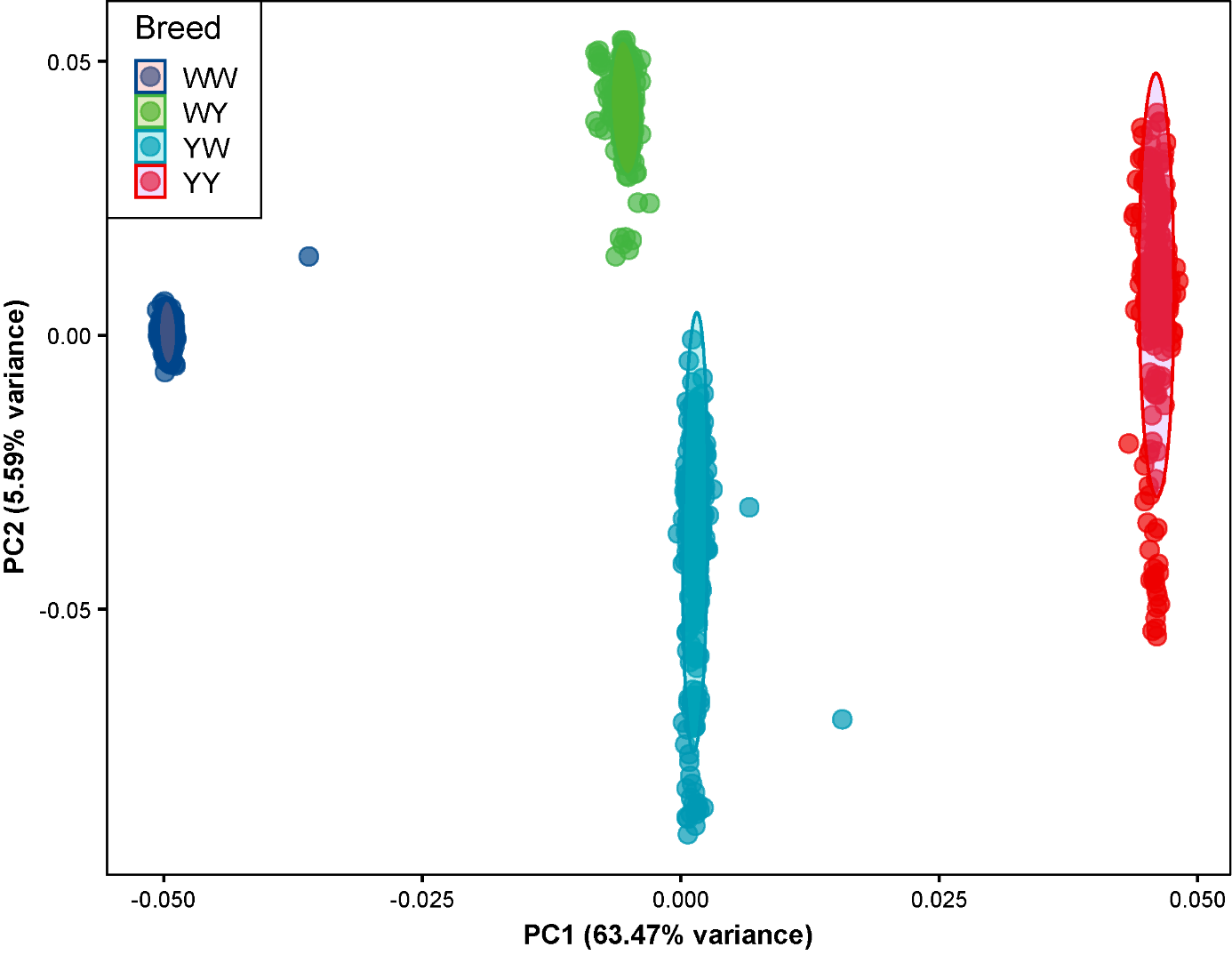


**Fig. S8.** PCA plot for the four genetic groups


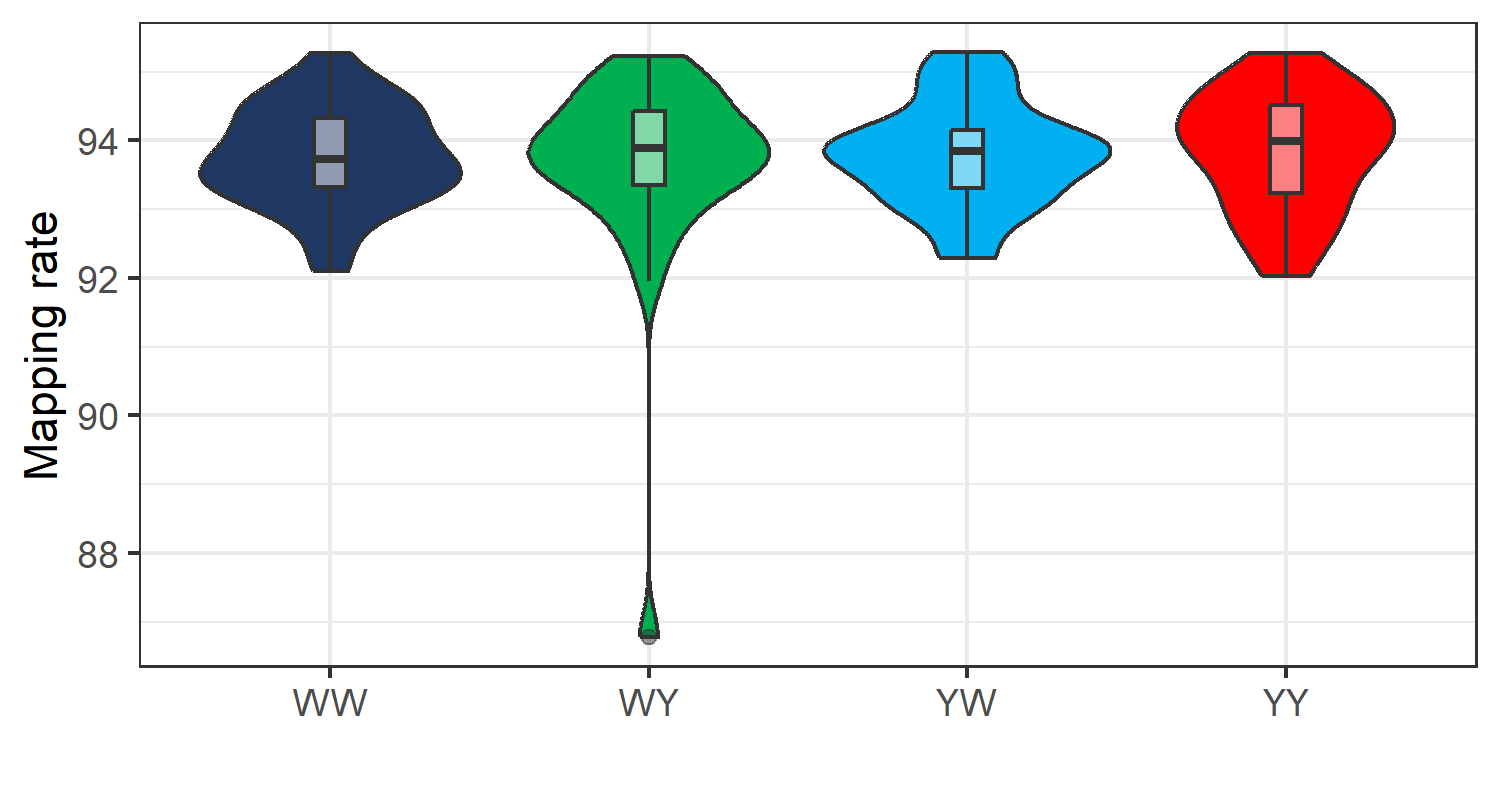


**Fig. S9.** Mapping quality of transcriptome data


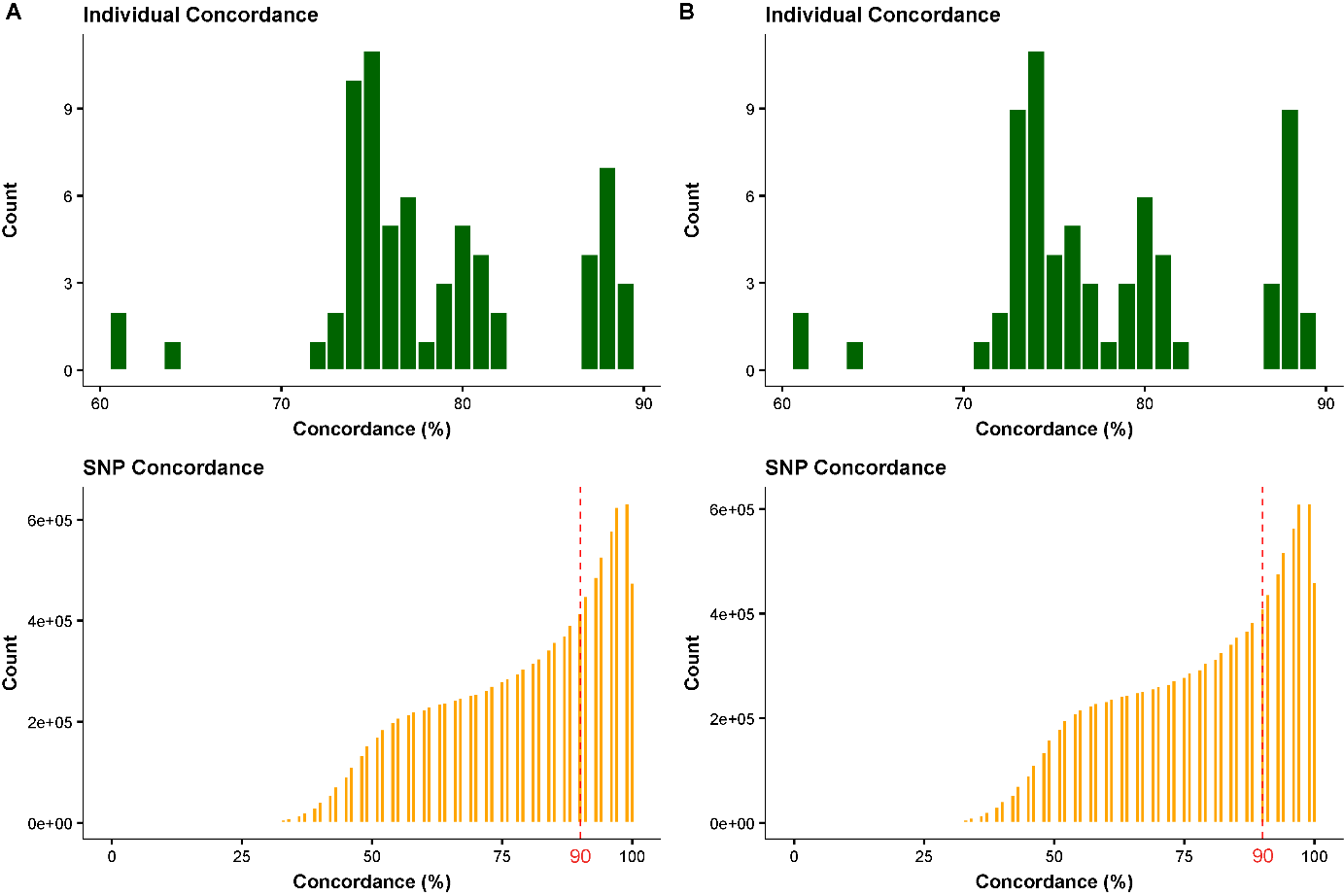


**Fig. S10.** Distribution of concordance for individual and SNPs for different imputation methods. **(A)** Concordance of whole-genome sequencing and transcriptome sequencing data when imputing across genetic groups. **(B)** Concordance of whole-genome sequencing and transcriptome sequencing data when imputing within genetic groups.


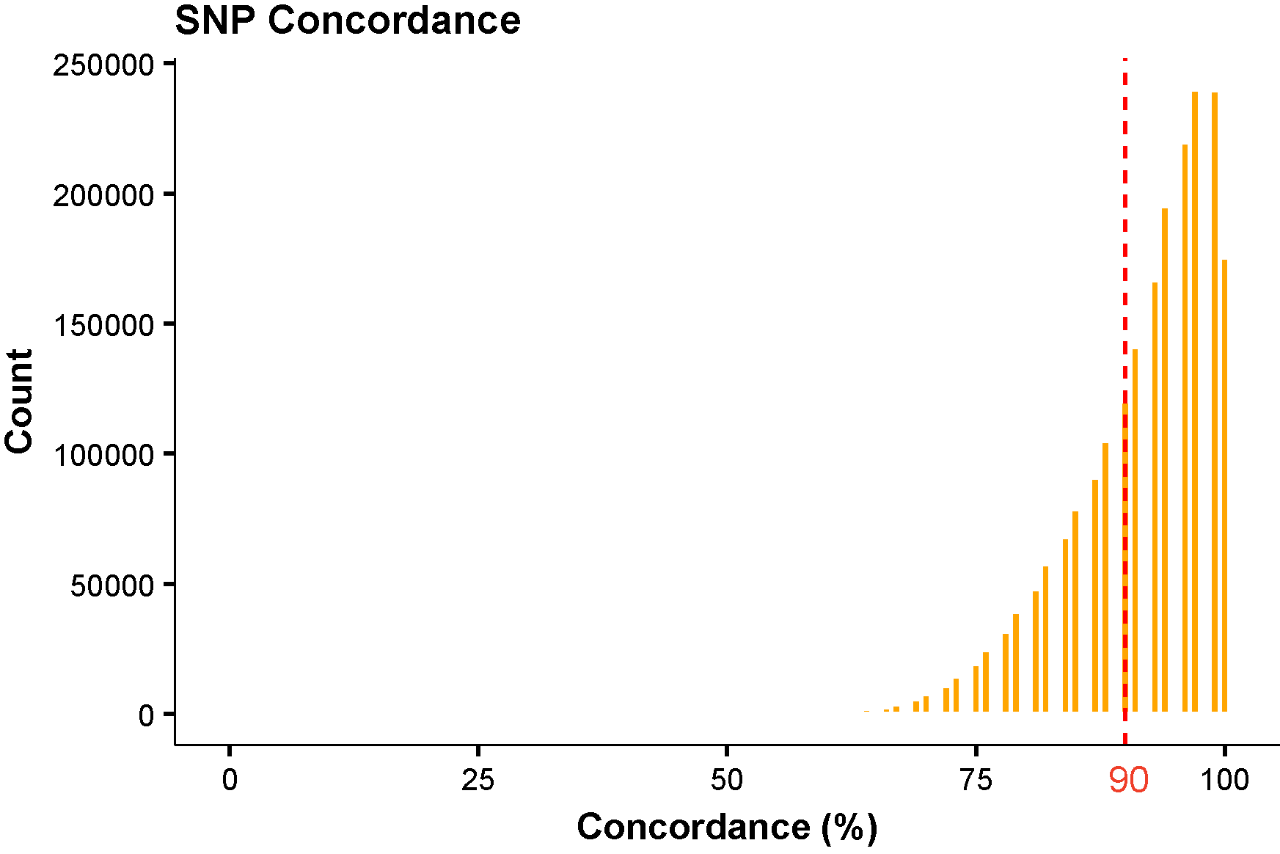


**Fig. S11.** Distribution of concordance for SNPs when applying missing genotype smaller than 10%.


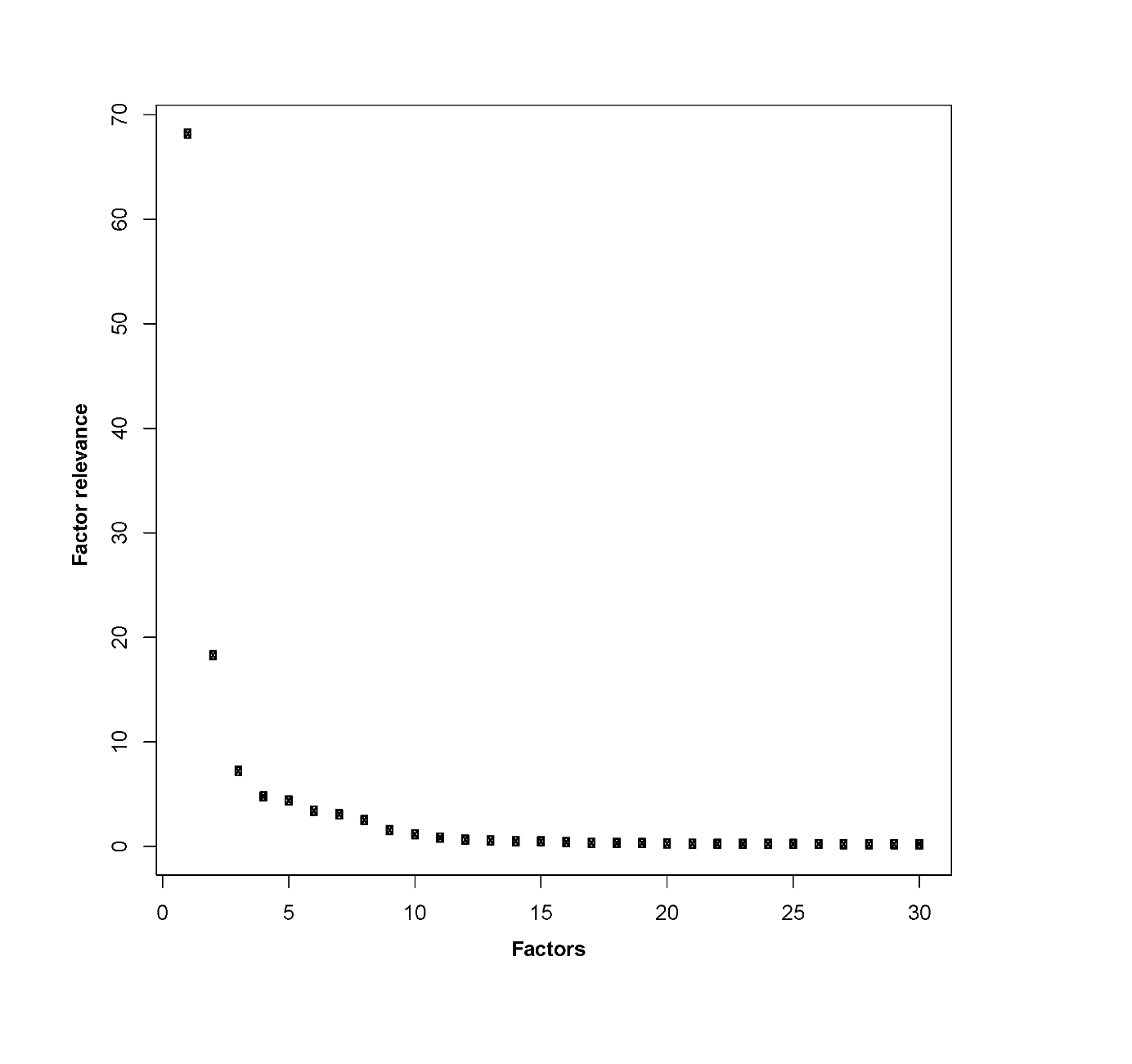


**Fig. S12.** Diagnostic plot of the factor relevance (automatic relevance determination parameters).


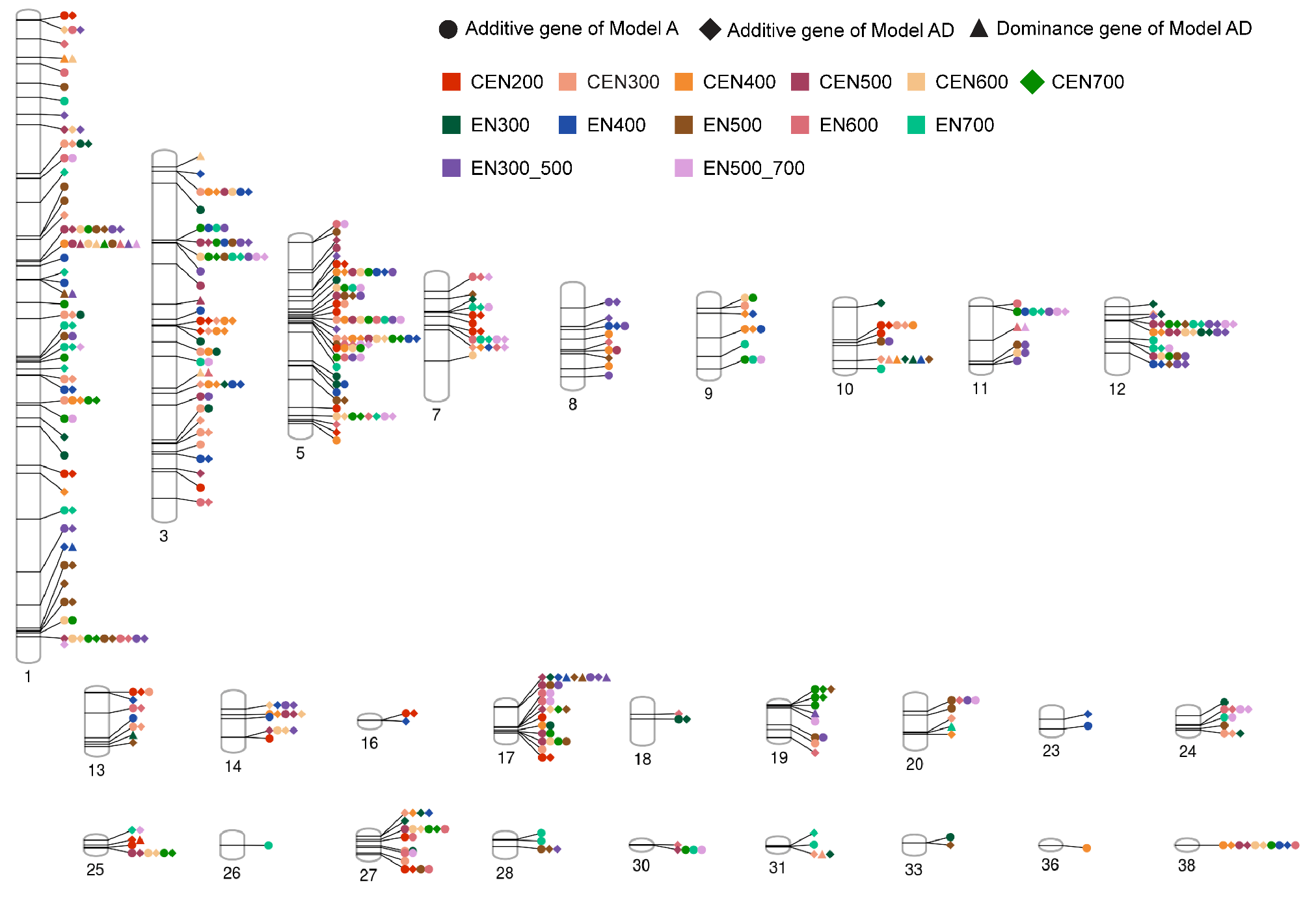


**Fig. S13.** Genes identified by TWAS analysis. PhenoGram of statistically significant gene-trait associations identified by S-predixcan in whole genome expect chromosomes 2, 4, 6, 15 and 21. Each association is arranged according to the SNP location on each chromosome and the points are color-coded by traits. Circle represented additive SNP from additive model, diamonds represented additive SNP from additive-dominance model, and triangle represented dominance SNP from additive-dominance model.


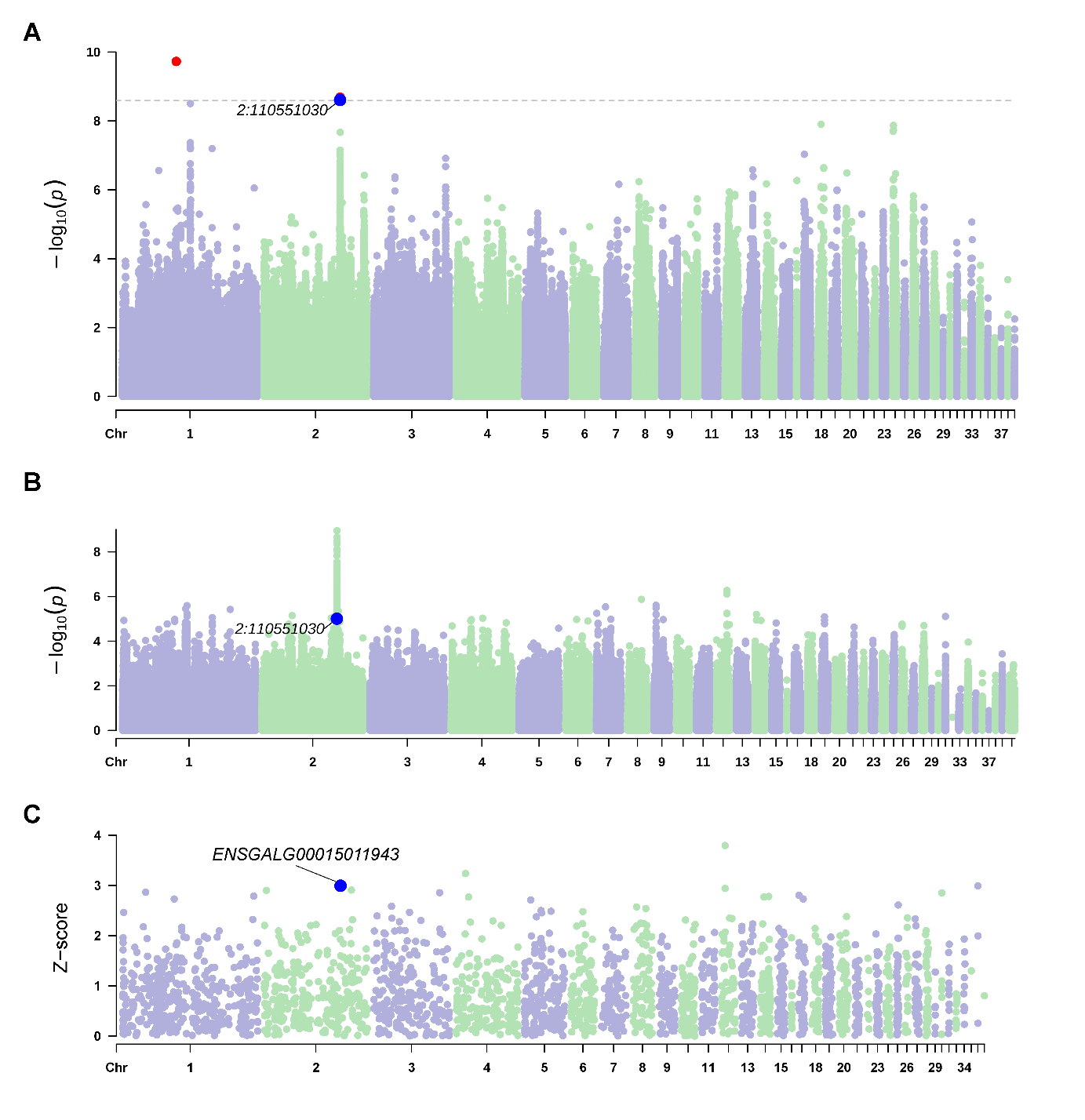


**Fig. S14.** Multi-omics data analysis of genetic determinants underlying cumulative egg number till 500 days of age for additive SNP effects. **(A)** GWAS for trait CEN500. Each dot represent one SNP, and associated SNPs were colored in red with threshold FDR 0.01. Blue dot point out candidate SNP. **(B)** eQTL mapping for gene *ENSGALG00015011943*. Each dot represent one SNP. Blue dot point out candidate SNP. **(C)** TWAS for trait CEN500. Each dot represent one gene. Blue dot point out candidate gene.

**
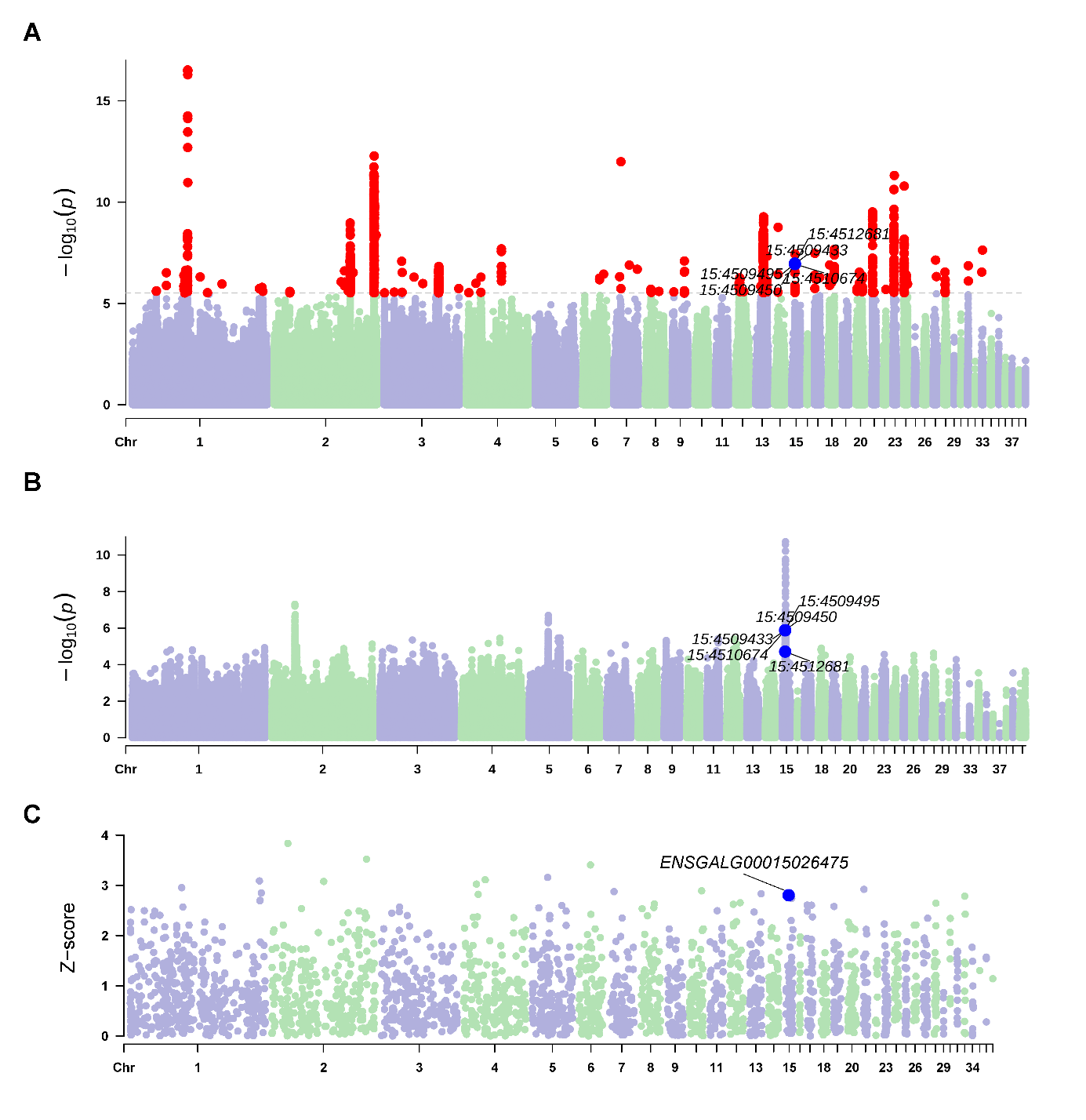
**

**Fig. S15.** Multi-omics data analysis of genetic determinants underlying egg number between 400 and 500 days of age for additive SNP effects. **(A)** GWAS for trait CEN500. Each dot represents one SNP, and associated SNPs were colored in red with threshold FDR 0.01. Blue dots point out candidate SNPs. **(B)** eQTL mapping for gene *ENSGALG00015026475*. Each dot represents one SNP. Blue dots point out candidate SNPs. **(C)** TWAS for trait EN500. Each dot represent one gene. Blue dot point out candidate gene.

**
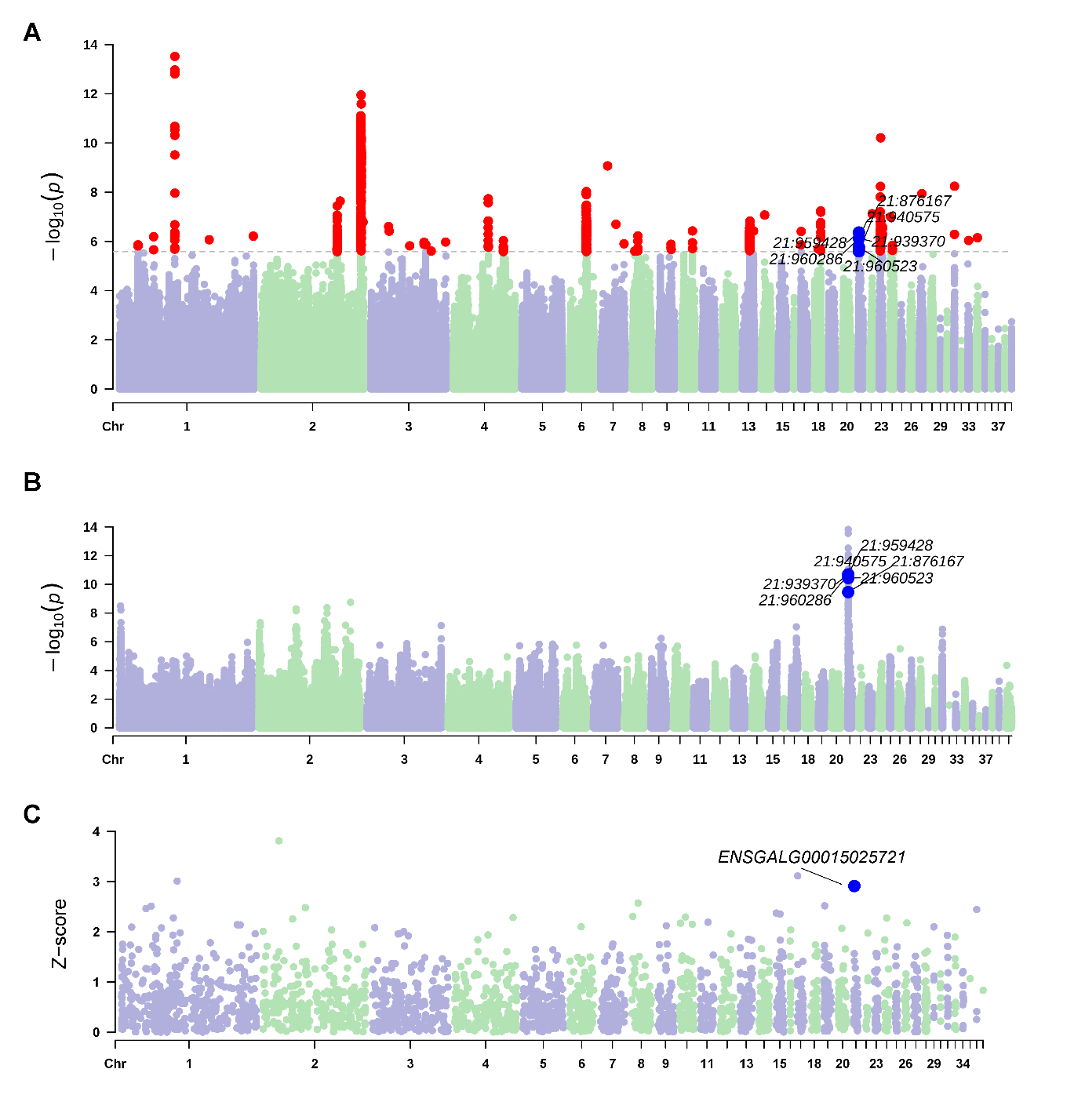
**

**Fig. S16.** Multi-omics data analysis of genetic determinants underlying egg number between 400 and 500 days of age for dominance SNP effects. **(A)** GWAS for trait EN500. Each dot represent one SNP, and associated SNPs were colored in red with threshold FDR 0.01. Blue dots point out candidate SNPs. **(B)** eQTL mapping for gene *ENSGALG00015025721*. Each dot represent one SNP. Blue dots point out candidate SNPs. **(C)** TWAS for trait EN500. Each dot represent one gene. Blue dot point out candidate gene.


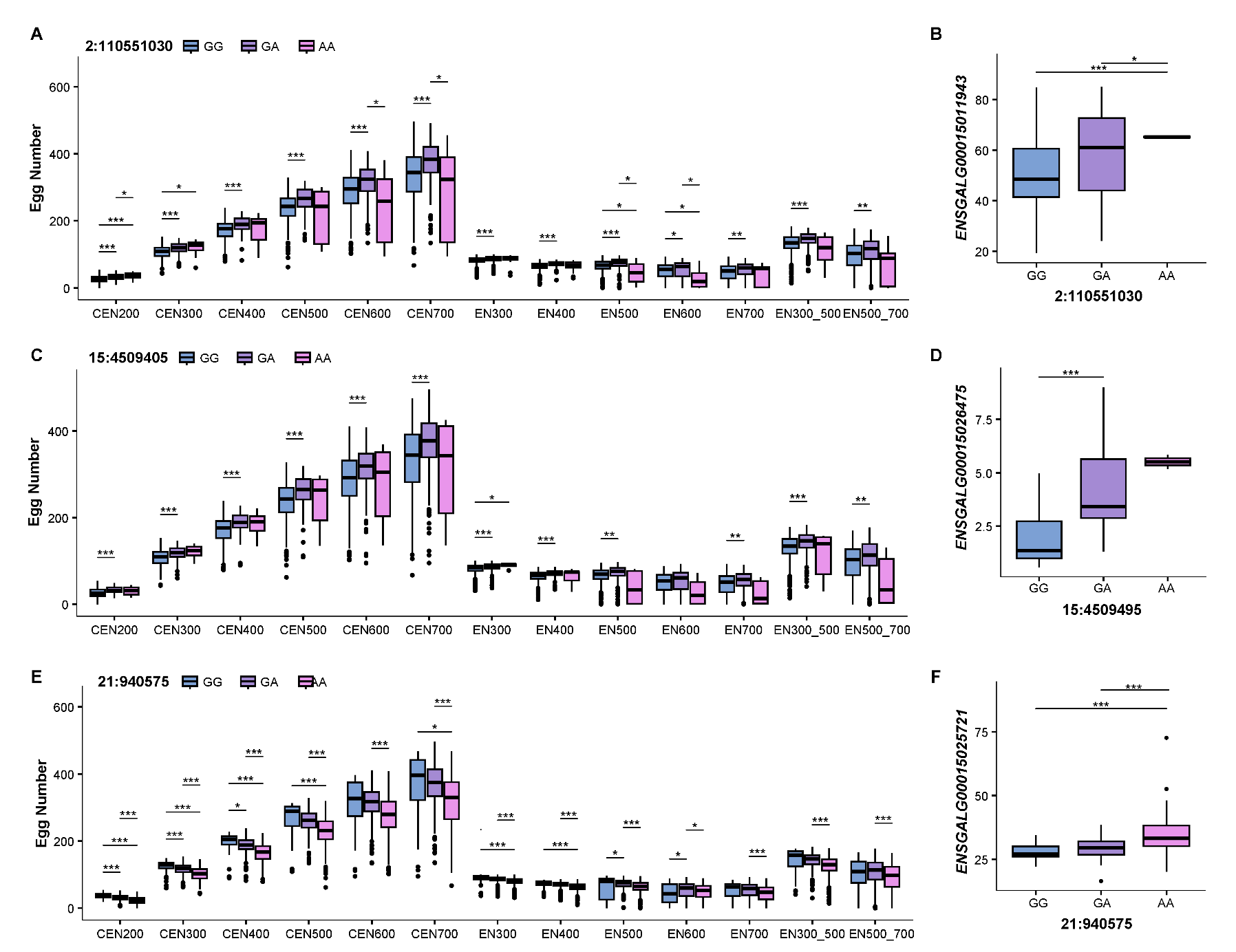


**Fig. S17.** The correlations between candidate variants and phenotype, and expression of candidate genes. **(A)** Egg number across genotypes of SNP 2:110551030. **(B)** The expression level of gene *ENSGALG00015011943* across genotypes of SNP 2:110551030. **(C)** Egg number across genotypes of SNP 15:4509495. **(D)** The expression level of gene *ENSGALG00015026475* across genotypes of SNP 15:4509495. **(E)** Egg number across genotypes of SNP 21:940575. **(F)** The expression level of gene *ENSGALG00015025721* across genotypes of SNP 21:940575. (^*^: *P* < 0.05, ^**^: *P* < 0.01, ^***^: *P* < 0.001)


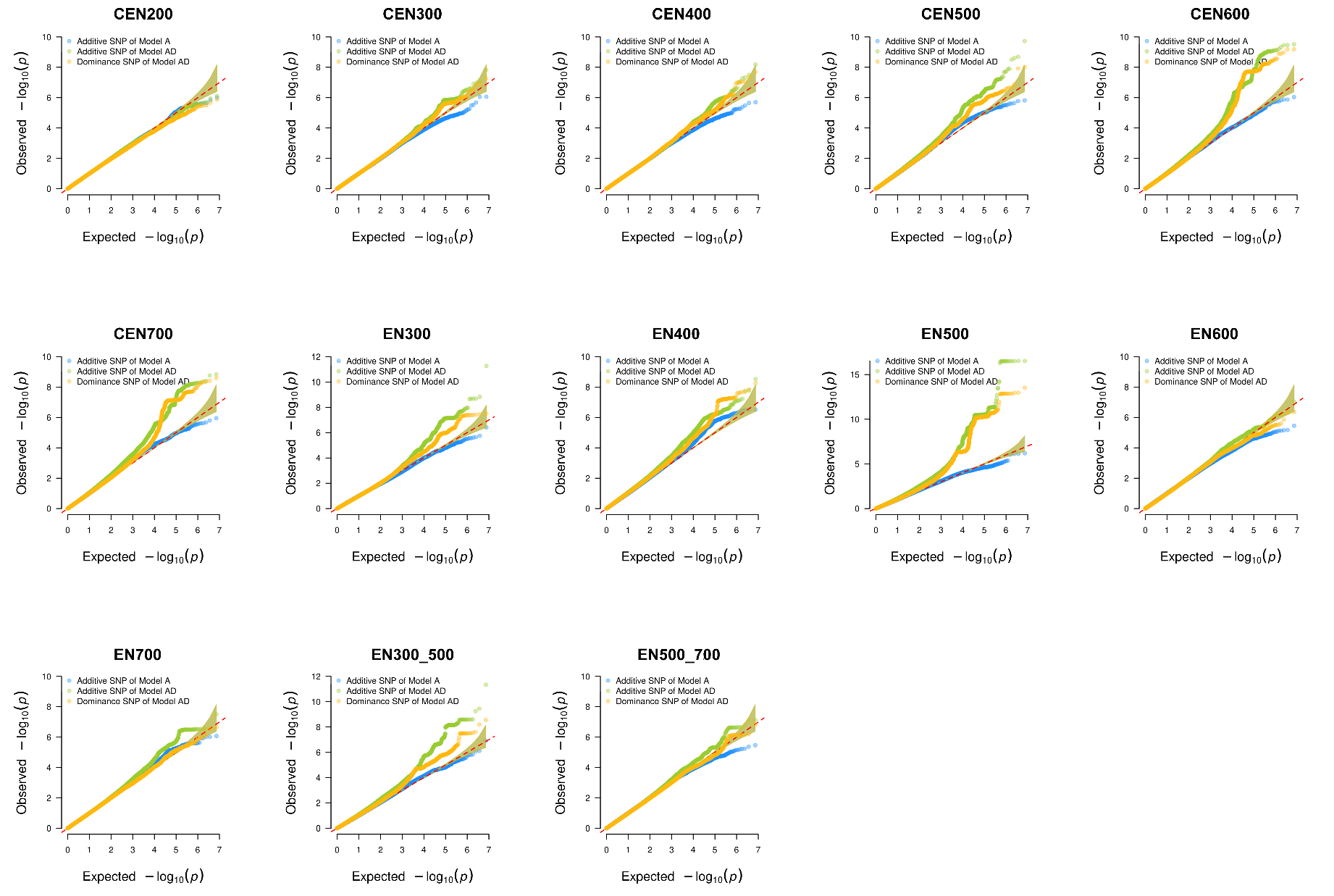


**Fig. S18.** QQplots across traits. Blue dots are the additive SNP effects of Model A, green dots are the additive SNP effects of Model AD, yellow dots are the dominance SNP effects of Model AD.


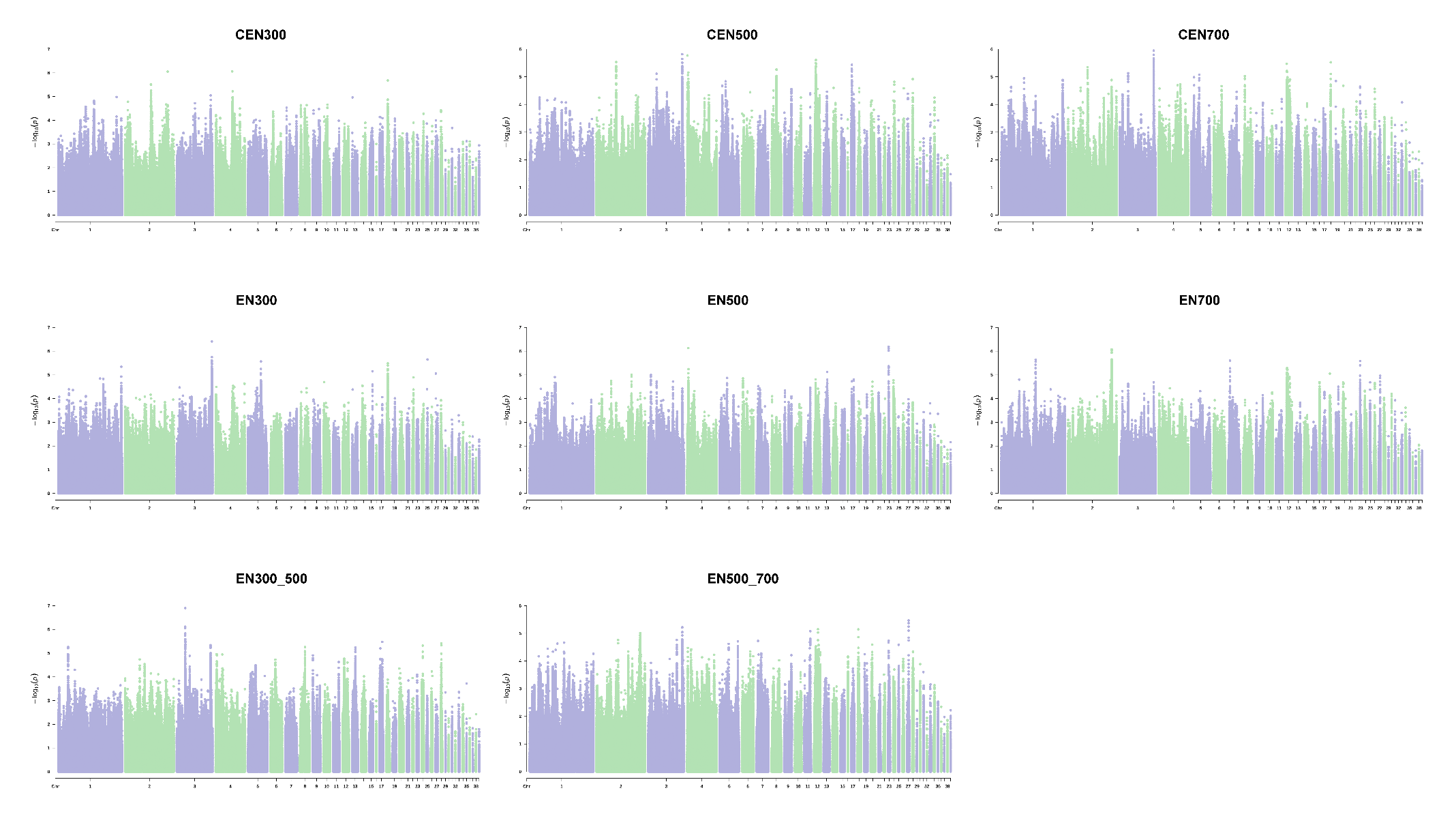


**Fig. S19.** Manhattan plots of additive SNP effects for cumulative egg number and egg number at different stages in Model A.

**
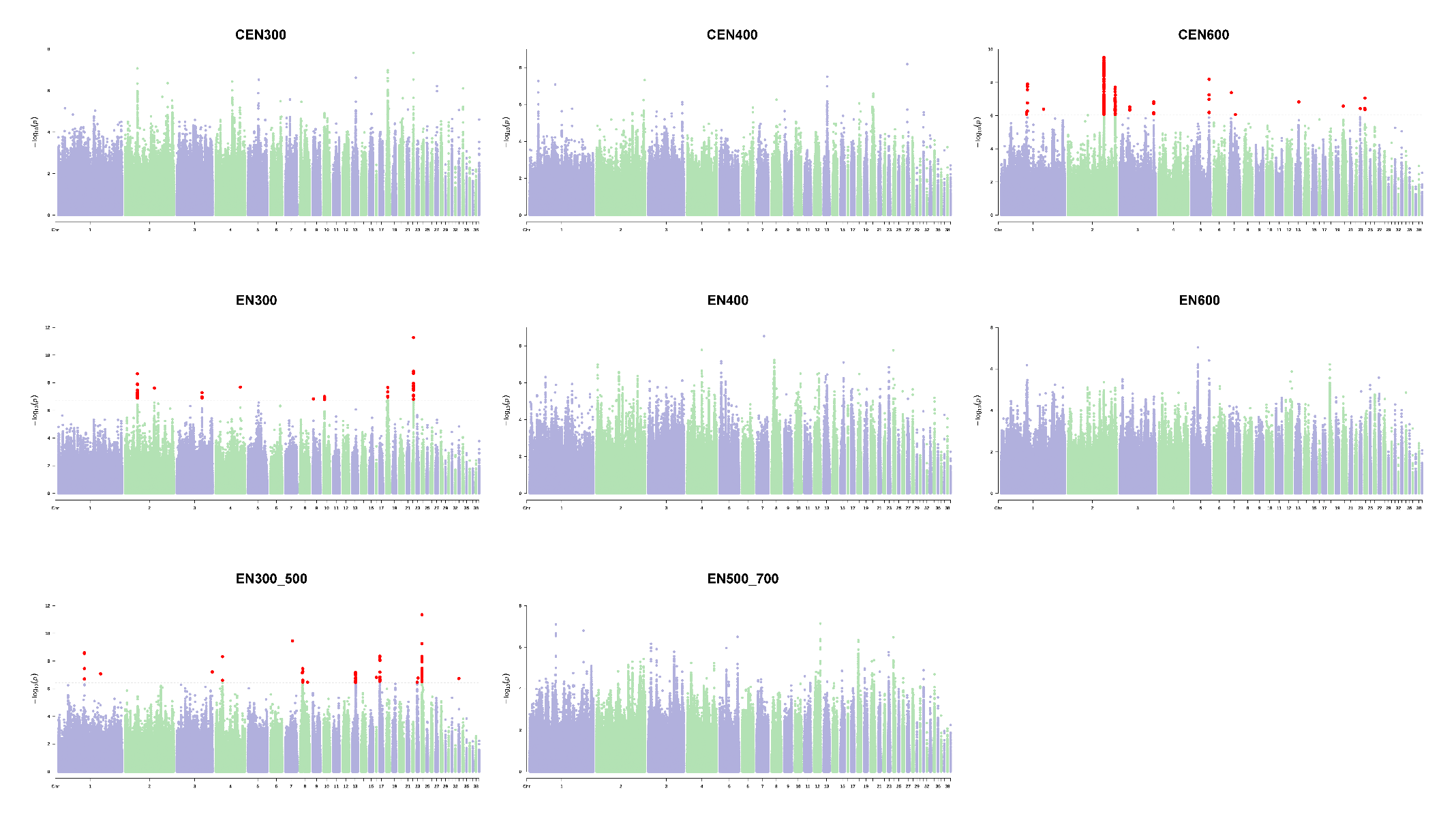
**

**Fig. S20.** Manhattan plots of additive SNP effects for cumulative egg number and egg number at different stages in Model AD.

**
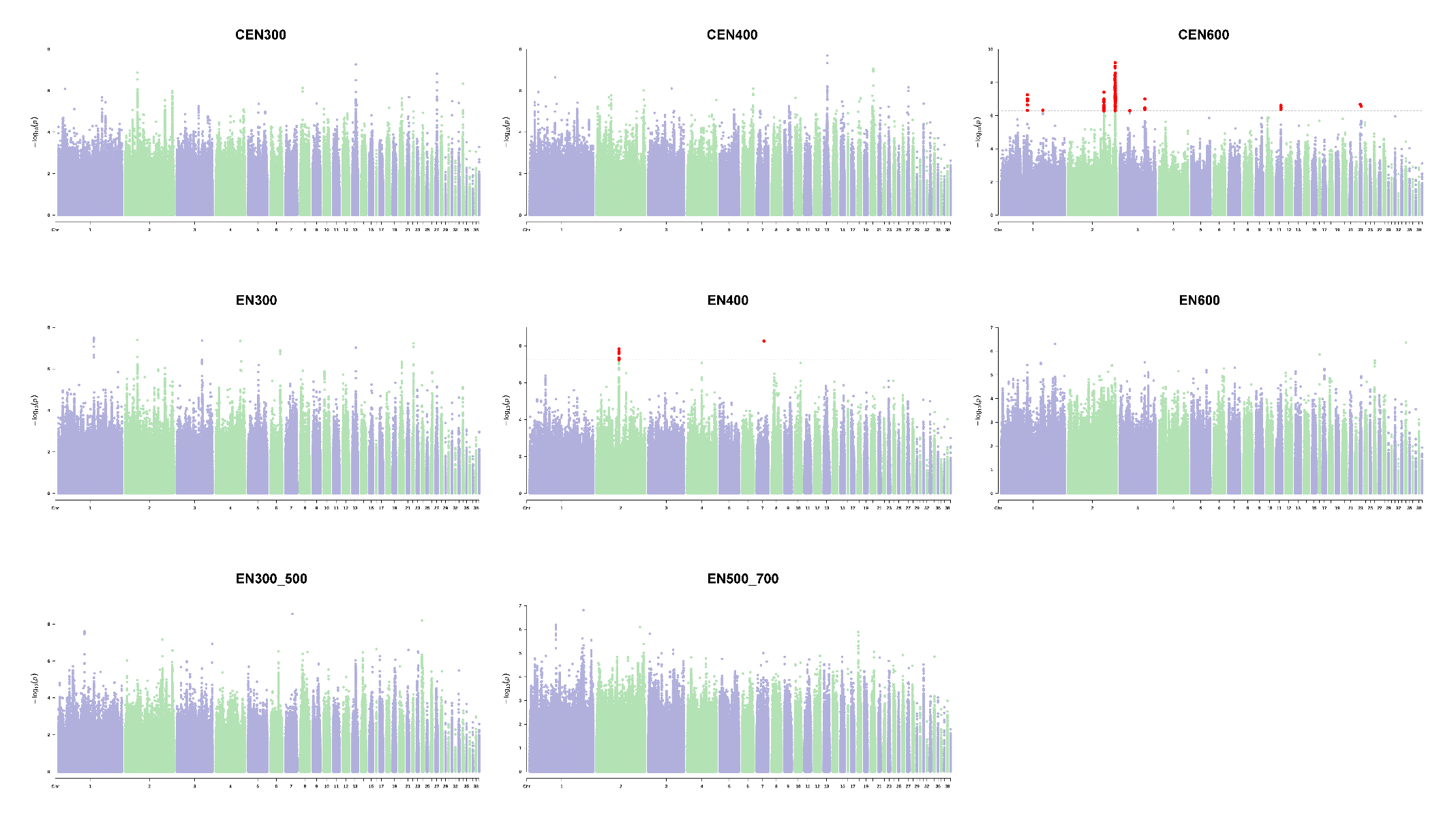
**

**Fig. S21.** Manhattan plots of dominance SNP effects for cumulative egg number and egg number at different stages in Model AD.


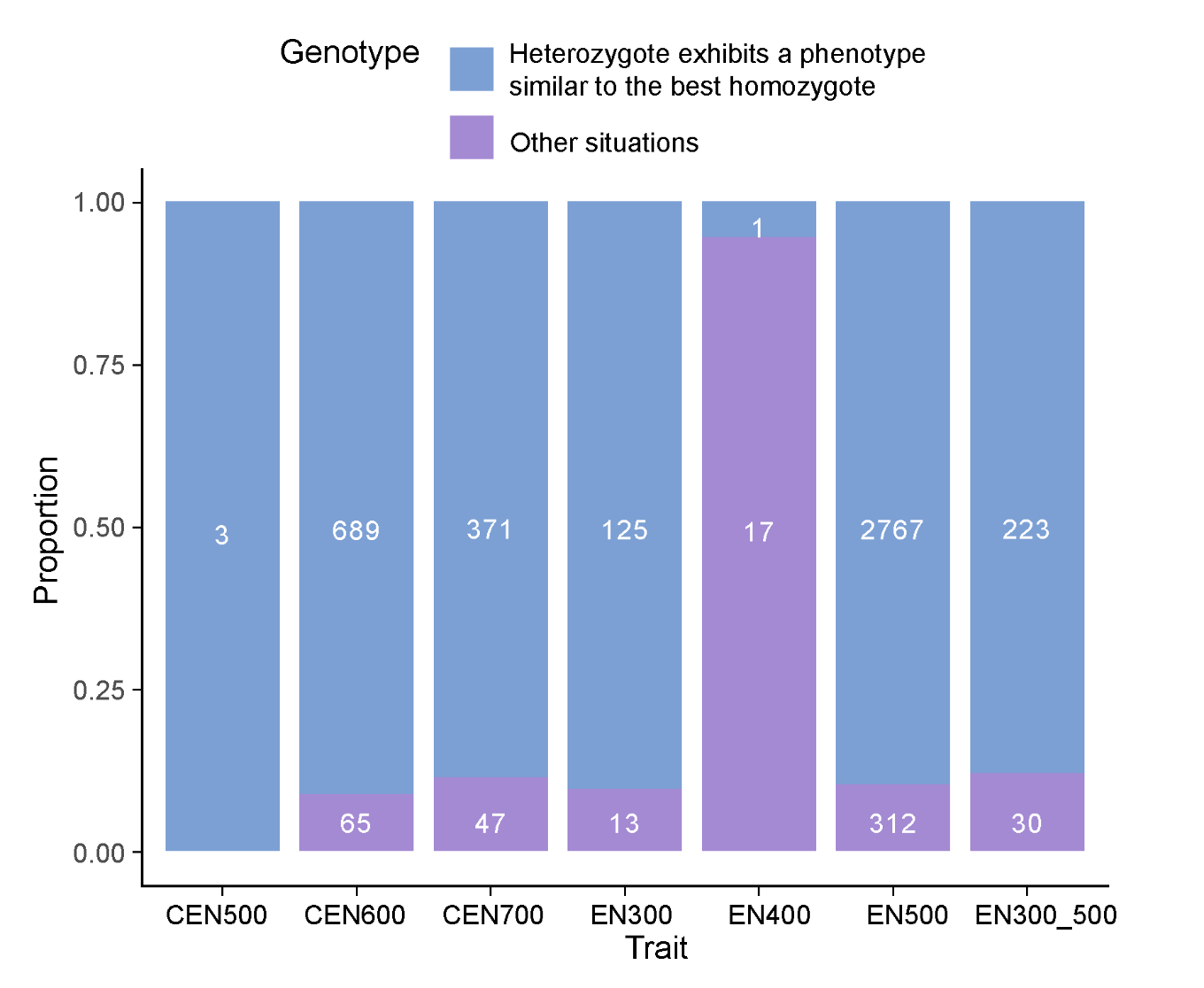


**Fig. S22.** Proportion of significant SNPs where heterozygotes exhibit a phenotype similar to the phenotype of the best homozygote.


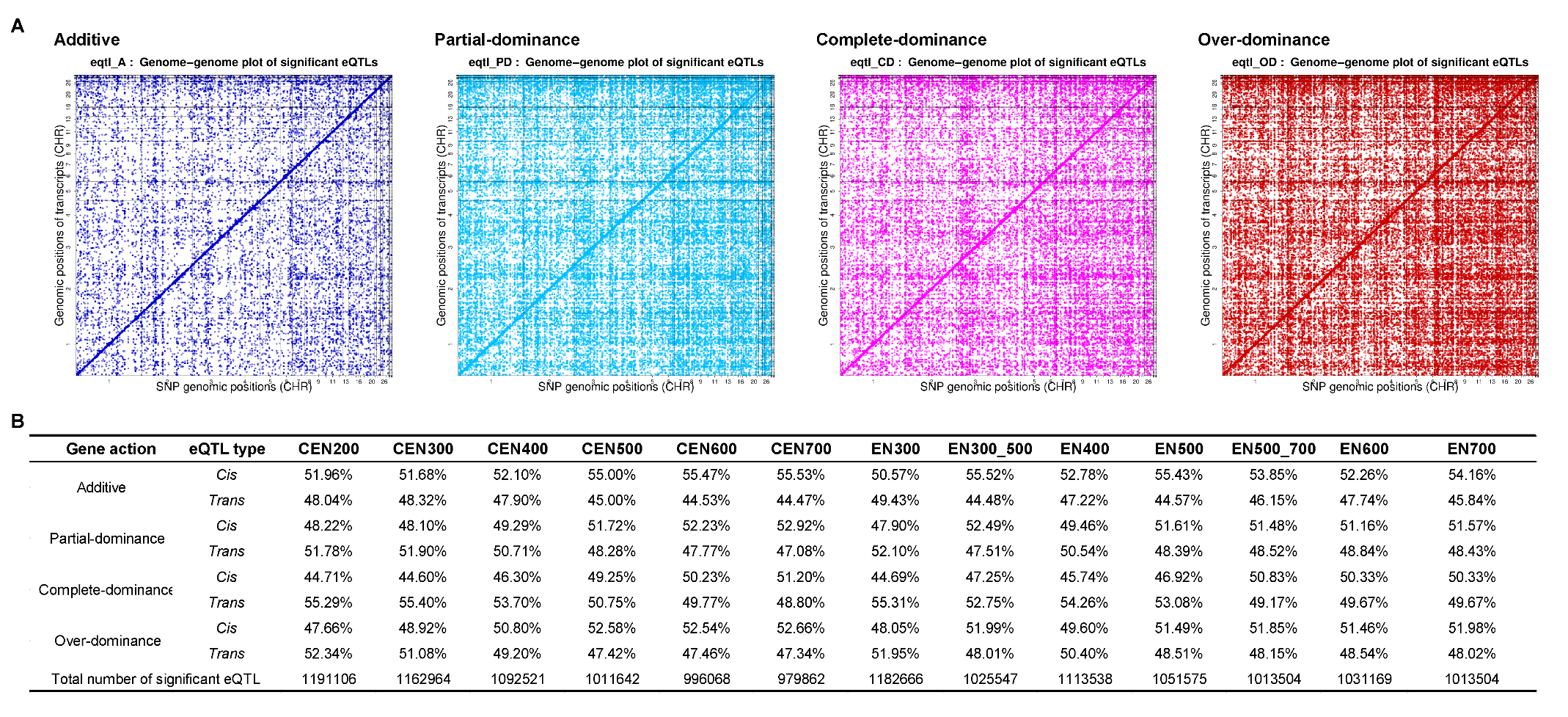


**Fig. S23.** *Cis-* and *trans*-acting eQTLs and their associated genes. **(A)** SNP genomic position and genomic position of significantly associated genes. **(B)** Quantification of *cis-* and *trans-*acting eQTLs across each filtered based on SNP ratio.


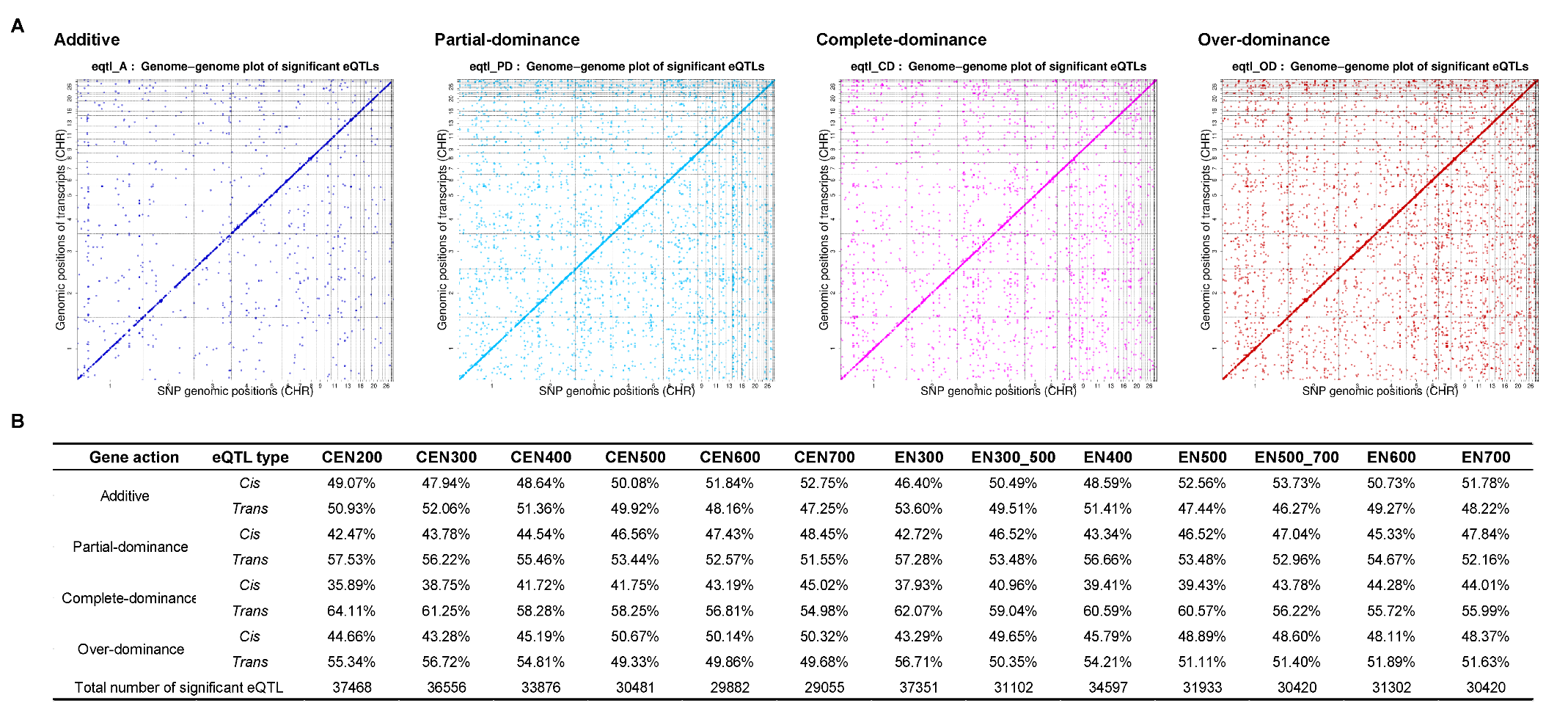


**Fig. S24.** *Cis-* and *trans*-acting eQTLs and their associated genes after pruning with linkage equilibrium. **(A)** SNP genomic position and genomic position of significantly associated genes. **(B)** Quantification of *cis-* and *trans-*acting eQTLs across each filtered based on SNP ratio.


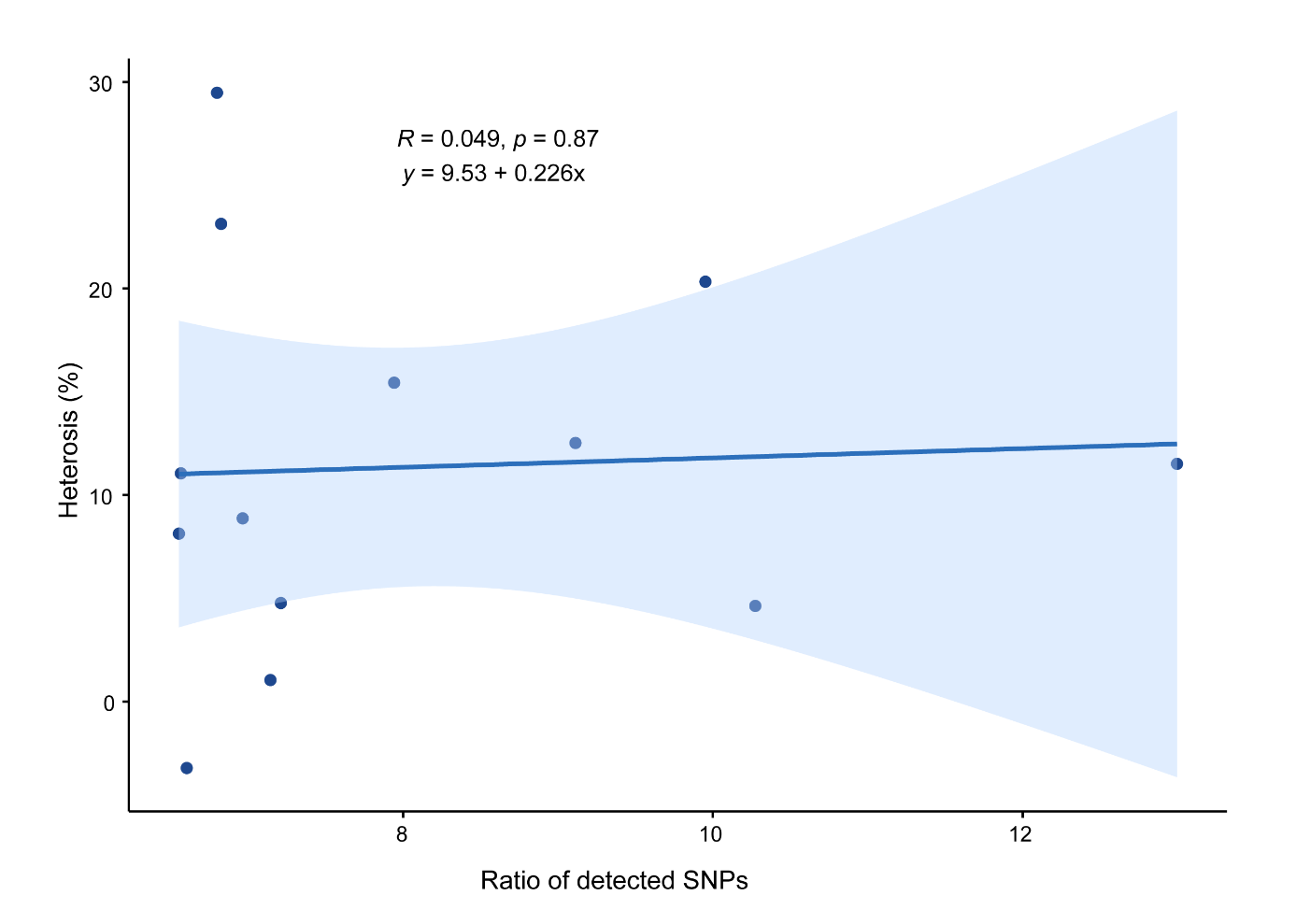


**Fig. S25.** Correlation between SNP ratios of all SNPs and heterosis
