# Supplementary material for: Identifying candidate genetic variants for egg number by analyzing over 1,000 fully sequenced layers
Source: Gigascience. 2025 Jun 17;14:giaf064. doi: 10.1093/gigascience/giaf064 (PMC12203006; doi:10.1093/gigascience/giaf064)
Supplement: giaf064_GIGA-D-24-00467_original_submission [file giaf064_giga-d-24-00467_original_submission.pdf]

GigaScience

Identifying candidate genetic variants for egg number by analyzing over 1000 fully sequenced layers

--Manuscript Draft--

**Manuscript Number:**	GIGA-D-24-00467
**Full Title:**	Identifying candidate genetic variants for egg number by analyzing over 1000 fully sequenced layers
**Article Type:**	Research
**Funding Information:**	National Natural Science Foundation of China (32172721) — Prof. Yanyan Sun<br>Agriculture Research System of China (CARS-40) — Prof. Jilan Chen<br>Agricultural Science and Technology Innovation Program (ASTIP-2021-IAS-16) — Prof. Jilan Chen
**Abstract:**	Background: Egg production over a long laying cycle until 700 days of age is fancy for modern layer chickens breeding. It is supposed to be influenced by the onset of laying, stability during the peak period, and persistence at late laying stages. Conventional single- single nucleotide polymorphisms (SNP) association analyses have identified additive loci, but few studies have explored dominance effects or integrated multi-omics data to investigate the genetic basis of egg production traits from the onset to 700 days of age.A full diallel cross of 1,004 chickens was·subjected to whole-genome sequencing. Transcriptome data from the ovary was available for a subset of the chickens. Genome-wide association study (GWAS) was conducted using an additive-dominance model for cumulative egg number and egg number at different stages. Transcriptome-wide association study (TWAS) was used to explore the associations between gene expression and investigated traits to identify candidate genes.<br>Results: The additive-dominance model identified 5,892 significant SNPs, with 805 additive SNPs and 360 dominance SNPs shared between two or more traits. By integrating loci identified through GWAS with expression quantitative trait loci (eQTL) mapping, the expression level of 46 genes were found to be associated with significant SNPs. Further intersection with TWAS results revealed three novel candidate genes. For the loci with significant SNP effects, we found a positive but insignificant correlation between the ratios of dominance to additive effects and observed heterosis. Observed heterosis was positively correlated with heterosis predicted based on dominance effects and allele frequencies of all SNPs.<br>Conclusions: We identified candidate genetic variants for egg production traits by analyzing 1,004 fully sequenced layers. Detection benefited from incorporating dominance into GWAS model. Traits with higher heterosis tended to be more affected by genes with dominant mode of action. Moreover, multi-omics data allowed to contribute to deciphering genetic mechanisms underlying egg production by establishing connections between genetic variants, gene expression, and egg number.<br>Key words: whole-genome sequencing, genetic variants, egg number, additive-dominance model, multi-omics, TWAS, heterosis
**Corresponding Author:**	Jilan Chen<br>Chinese Academy of Agricultural Sciences Institute of Animal Science<br>Beijing, CHINA
**Corresponding Author Secondary Information:**	
**Corresponding Author's Institution:**	Chinese Academy of Agricultural Sciences Institute of Animal Science
**Corresponding Author's Secondary Institution:**	
**First Author:**	Aixin Ni
**First Author Secondary Information:**	

Powered by Editorial Manager® and ProduXion Manager® from Aries Systems Corporation

Order of Authors:	Aixin Ni
	Henk Bovenhuis
	Mario P.L. Calus
	Yunlei Li
	Jingwei Yuan
	Yanyan Sun
	Jilan Chen
Order of Authors Secondary Information:	
Additional Information:	
Question	Response
Are you submitting this manuscript to a special series or article collection?	No
**Experimental design and statistics**<br><br>Full details of the experimental design and statistical methods used should be given in the Methods section, as detailed in our Minimum Standards Reporting Checklist. Information essential to interpreting the data presented should be made available in the figure legends.<br><br>Have you included all the information requested in your manuscript?	Yes
**Resources**<br><br>A description of all resources used, including antibodies, cell lines, animals and software tools, with enough information to allow them to be uniquely identified, should be included in the Methods section. Authors are strongly encouraged to cite Research Resource Identifiers (RRIDs) for antibodies, model organisms and tools, where possible.<br><br>Have you included the information requested as detailed in our Minimum Standards Reporting Checklist?	Yes
**Availability of data and materials**	Yes

Powered by Editorial Manager® and ProduXion Manager® from Aries Systems Corporation

All datasets and code on which the conclusions of the paper rely must be either included in your submission or deposited in [publicly available repositories](#) (where available and ethically appropriate), referencing such data using a unique identifier in the references and in the “Availability of Data and Materials” section of your manuscript.<br><br>Have you have met the above requirement as detailed in our [Minimum Standards Reporting Checklist](#)?	
GigaScience has policies and guidelines in place for the use of generative AI-writing tools such as ChatGPT. If you have used such writing tools to assist with writing the manuscript this must be declared and cited in the text. Authors should not list AI-writing tools and other AI-assisted technologies as an author or co-author and should acknowledge that they are fully responsible for text generated or refined by AI-writing tools.<p><br><br>A summary of use (particularly in the introduction or among methods) needs to be included at the end of the paper, and the outputs should also be included as a supplementary file hosted in GigaDB or other open repositories. Please read our guidelines for more information. <p><br><br>By submitting to GigaScience, you are aware of the journal's AI-writing tools policy, and if you have declared use of such tools below, you have acknowledged this where appropriate in your manuscript and have made a summary of use and outputs available. <p><br><b>AI-assisted writing tools have been used in the preparation of this manuscript?	No

Powered by Editorial Manager® and ProduXion Manager® from Aries Systems Corporation

Manuscript Click here to access/download;Manuscript;EP_AN_20241021.docx

Identifying candidate genetic variants for egg number by analyzing over 1000 fully sequenced layers

Aixin Ni[1,2], Henk Bovenhuis[2], Mario P.L. Calus[2], Yunlei Li[1], Jingwei Yuan[1], Yanyan Sun[1,*], Jilan Chen[1,*]

[1]State Key Laboratory of Animal Biotech Breeding, Key Laboratory of Animal (Poultry) Genetics Breeding and Reproduction of Ministry of Agriculture and Rural Affairs, Institute of Animal Science, Chinese Academy of Agricultural Sciences, 100193 Beijing, China

[2]Animal Breeding and Genomics, Wageningen University and Research, P.O. Box 338, 6700 AH Wageningen, the Netherlands

*Correspondence address: Yanyan Sun, Institute of Animal Science, Chinese Academy of Agricultural Sciences, 100193 Beijing, China. E-mail: sunyanyan02@caas.cn; Jilan Chen, Institute of Animal Science, Chinese Academy of Agricultural Sciences, 100193 Beijing, China. E-mail: chen.jilan@163.com

E-mail addresses:

Aixin Ni: aixin.ni@wur.nl

Henk Bovenhuis: henk.bovenhuis@wur.nl

Mario P.L. Calus: mario.calus@wur.nl

Yunlei Li: liyunlei@caas.cn

Jingwei Yuan: yuanjingwei@caas.cn

Yanyan Sun: sunyanyan02@caas.cn

Jilan Chen: chen.jilan@163.com

Abstract

Background: Egg production over a long laying cycle until 700 days of age is fancy for modern layer chickens breeding. It is supposed to be influenced by the onset of laying, stability during the peak period, and persistence at late laying stages. Conventional single-single nucleotide polymorphisms (SNP) association analyses have identified additive loci, but few studies have explored dominance effects or integrated multi-omics data to investigate the genetic basis of egg production traits from the onset to 700 days of age. A full diallel cross of 1,004 chickens was·subjected to whole-genome sequencing. Transcriptome data from the ovary was available for a subset of the chickens. Genome-wide association study (GWAS) was conducted using an additive-dominance model for cumulative egg number and egg number at different stages. Transcriptome-wide association study (TWAS) was used to explore the associations between gene expression and investigated traits to identify candidate genes.

Results: The additive-dominance model identified 5,892 significant SNPs, with 805 additive SNPs and 360 dominance SNPs shared between two or more traits. By integrating loci identified through GWAS with expression quantitative trait loci (eQTL) mapping, the expression level of 46 genes were found to be associated with significant SNPs. Further intersection with TWAS results revealed three novel candidate genes. For the loci with significant SNP effects, we found a positive but insignificant correlation between the ratios of dominance to additive effects and

observed heterosis. Observed heterosis was positively correlated with heterosis predicted based on dominance effects and allele frequencies of all SNPs.

Conclusions: We identified candidate genetic variants for egg production traits by analyzing 1,004 fully sequenced layers. Detection benefited from incorporating dominance into GWAS model. Traits with higher heterosis tended to be more affected by genes with dominant mode of action. Moreover, multi-omics data allowed to contribute to deciphering genetic mechanisms underlying egg production by establishing connections between genetic variants, gene expression, and egg number.

Key words: whole-genome sequencing, genetic variants, egg number, additive-dominance model, multi-omics, TWAS, heterosis

Background

The global egg production (in tons) has been doubled since 1990 and eggs play crucial roles in providing high-quality and low-cost animal protein for the growing population all along (1). Egg production is not only a reflection of laying efficiency and economic efficiency but also one of the most important breeding goal traits for laying hens. Combing genomic information and knowledge of the genetic architecture of traits are of increasing importance in selective breeding. Nowadays, to reduce brooding costs and to lower environmental impact, there is a trend to extend the laying cycle from the traditional 500 to 700 days of age (2). This emphasizes the need for studies to look for loci underlying egg production traits throughout the laying period, especially at the extended period, to provide insights for selective breeding.

Quantitative trait loci (QTL) mapping and genome-wide association study (GWAS) have revealed genomic variants statistically associated with egg production traits. About 115 QTL on 27 chromosomes were reported to be associated with egg number

in chickens (https://www.animalgenome.org/cgi-bin/QTLdb/GG/index). While GWAS establishes the connection between genotype and phenotype, the underlying biological mechanisms remain unclear. Furthermore, most of the identified variants are located in non-coding regions and several variants are in high linkage disequilibrium (LD), making it difficult to determine the causal variants. These issues can be alleviated by using multi-omics strategies such as expression quantitative trait loci (eQTL) mapping, transcriptome-wide association study (TWAS) and Mendelian randomization (MR) analysis to unravel the underlying genetic architecture of complex traits. eQTL-mapping identifies genomic regions associated with the expression levels of genes based on single nucleotide polymorphisms (SNP) genotypes and gene expression data. TWAS establishes a connection between gene expression and phenotypes by predicting gene expression levels in genotyped animals. It then leverages the summary-level GWAS results and expression data to identify the expression of gene and phenotypes associations. MR provides evidence for putative causal relations between gene expression and phenotypes (3). The joint analysis of genomic and transcriptomic data has contributed to deciphering the biological functions of candidate genes for various traits in cattle (4), pigs (5, 6), and chickens (7).

Another limitation of many GWAS studies is that non-additive effects are usually ignored, while with few focusing on dominance effects. Dominance is believed to be common in mammals (8) and has been studied in the context of genetic parameter estimation, genomic selection, and genomic prediction in many farm animals in traits such as carcass weight of cattle (9), body weight of quails (10), reproductive performance of Holstein dairy cattle (11), and growth of tilapia (12). In chickens, Amuzu-Aweh found that dominance variance accounted for up to 37% of the genetic variance, and up to 6% of the phenotypic variance in egg number depending upon the

line (13), highlighting the substantial role of non-additive genetic effects. Furthermore, loci with dominance effects have been identified in several species with different traits, contributing to phenotypic variance. In cattle, some semen production traits showed a reduction of 1.03 to 2.17 adjusted phenotypic standard deviations when comparing one homozygous genotype to the others (14). Dominance loci could explain 12% to 13% of variance in sheep's resistance to *H. contortus* and 0.69% to 0.84% of variance in broilers' egg number (15, 16). These examples underscore the importance and potential of considering dominance effects to gain a more comprehensive understanding of the genetic architecture underlying complex traits.

Examining the role of SNPs, particularly non-additive effects, might provide valuable insights into heterosis, which is thought to be driven by non-additive genetic effects (17). Quantitative genetic theory suggests that heterosis, expressed as the difference between the crossbreds and the mid-parent value, is proportional to the sum of the dominance effects multiplied by the squared difference in allele frequency between the parental lines (17). Moreover, the integration of genomic and transcriptomic data has led to the identification of heterosis-related genetic variants in several plants. For instance, *RH8* was identified as a heterosis-related candidate gene for yield in rice (18), structural variants in *ZAR1* and *ZmACO2* were found to increase yield heterosis in maize (19), and a CACTA-like transposable element upstream of *BnaA9.CYP78A9* was shown to contribute to the heterosis of cell number in oilseed rape (20). In the current study, we aimed to identify additive and non-additive candidate genetic variants for egg number, and explore the genetics underlying these complex traits using multi-omics data, and discuss implications for heterosis. To achieve our objective, we sequenced the genome of 1,004 animals, which enabled including all genome-wide segregating variants in the analysis, and would increase the power to

detect variants associated with egg production. We employed a model incorporating both additive and dominance SNP effects and combined this with transcriptome data of ovary tissue to map eQTLs. These analyses were followed by a TWAS to prioritize candidate genes for egg production traits. The flowchart of analyses to identify candidate genetic variants for egg production traits is shown in Fig. 1. Estimated dominance SNP effects were used to investigate its relationship with observed heterosis.

Methods

Resource population and phenotypic data

Animal resources used in our study were previously described in detail (21). Briefly, four genetic groups were used, pure line Beijing-You chickens (YY) and White Leghorns (WW); and their reciprocal crosses with either Beijing-You (YW) or White Leghorns (WY) as the sire line. The YW and YY animals were created using the same 30 Beijing-You sires, and the WY and WW were created using the same 30 White Leghorns sires. The chickens were kept in individual cages in the same hen house during the experiment. Cumulative egg number and egg number at different stages from 200, till 700 days of age were recorded, CEN200 (cumulative egg number till 200 days of age), CEN300, CEN400, CEN500, CEN600, CEN700, EN300 (egg number from 200 to 300 days of age), EN400, EN500, EN600, EN700, EN300_500, EN500_700, were computed from individual egg-laying recordings. In the current study, we defined the time period before 300 days of age as the early stage, 300 to 500 days of age as the middle stage, and 500 to 700 days of age as the late stage. Observed heterosis was estimated for different traits based on the predicted mean phenotypes for each genetic group, using the “predict” statement in ASReml 4.2 following the

model described previously (21).

Whole genome sequencing

Genomic DNA was extracted using the phenol-chloroform method. The genomes of 1,004 chickens were sequenced at ~15.98× coverage (Supplementary Fig. S1), containing 210 WW, 240 WY, 268 YY, and 286 YW. After sequencing, FastQC was used to evaluate the quality of sequencing (22). Trimmomatic was used to remove adapters and the low quality reads with the following parameters: LEADING:3 TRAILING:3 SLIDINGWINDOW:4:15 and MINLEN:15 (23).

Clean reads were aligned using BWA-MEM v0.7.17 (24). Samblaster v0.1.26 was used to mark duplicates (25), and Samtools v1.14 to sort and index the BAM files (26). Freebayes v1.3.1 was used with the chicken reference genome (GRCg7W) (27) for variant calling with: --use-best-n-alleles 4 --min-base-quality 10 --min-alternate-fraction 0.2 --haplotypelength 0 --ploidy 2 --min-alternate-count 2 (28). The vcffilter module from vcflib v0.00.2019.07.10 was used to discard variants with low phred quality score (<=20) (29). Tabix, a module from htslib v1.9, was used to index the VCF files (29). Alignment quality control statistics were computed with QualiMap v.2.2.2-dev (30).

A total of 16,828,475 variants were called. After removing indels with Plink v1.9 (31), 14,119,765 SNPs were retained. The data were further filtered with Plink using the following criteria: genotyping call-rate for SNPs < 95% and for individuals < 95%, minor allele frequency < 0.5%, and only for pure lines individuals a test for Hardy Weinberg equilibrium $P < 1e{-}4$.

After those filters, 12,495,895 SNPs and 986 animals were retained and subjected to a check for inconsistencies between pedigree and genomic information. Due to missing

genotypes from the parents, pedigree and genomic relationships were compared among the 986 animals. We removed conflicting animals manually using the following steps which were performed within WW, YY, and combined crossbred genetic groups: calculated pedigree and genomic relationships, and sorted the animals within the genetic groups based on descending absolute difference between pedigree and genomic relationships. In the top 100 of those relationships, we counted the occurrences for each animal. Starting from the top, we removed from every of those 100 relationships the animal that was involved in the largest number of “conflicts”. This process improved the credibility of the genomic data (Supplementary Figs. S2-S7). After quality control, 933 animals were used for subsequent analysis. Beagle (v.4.1) was used to impute the missing genotype (32). To avoid confounding between genetic group and genotype, SNPs were eliminated if a genotype was observed in both WW and YY lines with five or fewer animals.

Variance components estimation

Additive and dominance variances were estimated with restricted maximum likelihood method. For each trait, two models were fitted in Wombat (33): one with only additive effects (Model A), the other with additive and dominance effects (Model AD). Model A was:

$$\mathbf{y} = \mathbf{X_1}\mathbf{b_1} + \mathbf{u} + \mathbf{e}$$

where $\mathbf{y}$ was the vector with phenotypic values, $\mathbf{b_1}$ was a vector of the fixed effects including genetic group (WW, YY, YW and WY) and rack effects, $\mathbf{X_1}$ was the corresponding design matrix, $\mathbf{u}$ was the vector of the random animal effects with $N(\mathbf{0}, \mathbf{G}\sigma_a^2)$, where $\mathbf{G}$ was the genomic relationship matrix and σ_a^2 was the additive genetic variance, $\mathbf{e}$ was the vector of random residual effects with $N(\mathbf{0}, \mathbf{I}\sigma_e^2)$, where $\mathbf{I}$

was the identity matrix and σ_e^2 was the residual variance.

Model A was extended with a dominance deviation as Model AD:

$$\mathbf{y} = \mathbf{X_1 b_1} + \mathbf{u} + \mathbf{v} + \mathbf{e}$$

where $\mathbf{v}$ is a vector of random dominance deviations with $N(\mathbf{0}, \mathbf{D}\sigma_d^2)$, where $\mathbf{D}$ was the dominance relationship matrix and σ_d^2 was the dominance variance.

The genomic relationship matrix was computed with program Calc_grm according to the first version of VanRaden (34), $\mathbf{G} = \frac{\mathbf{ZZ'}}{2\sum_{p_i} p_i(1-p_i)'}$, $\mathbf{Z}$ is the matrix of all SNP genotypes of all individuals, and p_i is the frequency of the counted allele at SNP i. The dominance relationship matrix was computed with program Calc_grm according to Vitezica et al (35), $\mathbf{D} = \frac{\mathbf{MM'}}{\sum_i (2p_i(1-p_i))^2}$, where $\mathbf{M}$ is a matrix of heterozygous coefficients of all SNPs of all individuals. When individual j is homozygous for locus i, $M_{ij} = 0 - 2p_i(1-p_i)$, and when it is heterozygous, $M_{ij} = 1 - 2p_i(1-p_i)$.

Genome-wide association study

A single SNP GWAS was performed to estimate additive and dominance effects per SNP. For each SNP, the following model was fitted for Model A:

$$\mathbf{y} = \mathbf{X_1 b_1} + \mathbf{j}\alpha + \mathbf{u} + \mathbf{e}$$

where $\mathbf{j}$ was a vector with allele counts (coded as 0, 1, and 2 when homozygote for the alternative allele, heterozygote, and homozygote for the reference allele, respectively); α is the additive effect.

Model A was extended with a dominance deviation and a dominance effect as Model AD:

$$\mathbf{y} = \mathbf{X_1 b_1} + \mathbf{j}\alpha + \mathbf{k}\beta + \mathbf{u} + \mathbf{v} + \mathbf{e}$$

where **k** was a vector with heterozygosity status (coded as 0, 1, and 0 for genotype AA, AB, and BB, respectively); β is the dominance effect.

Solutions and t-statistics of the SNP effects were obtained from the output of Wombat, and corresponding P-values were computed. The genome-wide significance threshold for the SNP effects was based on a false discovery rate (FDR). FDR was calculated using the R-package "qvalue" and FDR < 0.01 was considered significant. Manhattan and Q-Q plots were derived from the GWAS results using the R-package "CMplot" (36). The variant effect predictor (VEP) software (37) was used to predict the maximal consequence of the all and significant SNPs. The SNP ratio was calculated based on the T-statistics from the Model AD, $r = \left|\frac{t_{Dom}}{t_{Add}}\right|$ (8). T-statistics (t_{Dom} and t_{Add}) is the ratio of the estimated SNP effect and its standard error. Based on the SNP ratios, SNPs were considered additive (r < 0.2), partial-dominant (0.2 < r < 0.8), complete-dominant (0.8 < r < 1.2), or over-dominant (r > 1.2) (8). In addition to the SNP ratios, we calculated the sum of the dominance effects multiplied by the squared difference in allele frequency between the parental lines (dy^2) for all SNPs and significant SNPs to investigate the relation to observed heterosis. To enable comparison across different traits, we standardized the dominance SNP effects with phenotypic standard deviation.

Transcriptome sequencing

From each of the four genetic groups, six chickens were randomly selected at 150, 250, 320, 500, and 700 days of age to collect ovaries for RNA sequencing, yielding 120 samples in total. Total RNA was isolated from the tissue of each hen using TRIzol® Reagent (Invitrogen, Carlsbad, CA, USA) according to the manufacturer's guidelines. RNA-seq was performed using Novaseq 6000 (Illumina, San Diego, USA) to generate 150 bp paired-end reads. Quality control, mapping, and transcriptome

assembly were done following the steps described previously (38), getting the transcripts per kilobase per million mapped reads (TPM) for each gene when mapped to the chicken reference genome (GRCg7W).

From the 120 samples, for 67 samples the genomic data was obtained from the whole genome sequencing. The genomic data of the remaining 53 animals was achieved using the following steps. STAR (v.2.7.11a) was used to map the high-quality reads to the chicken reference genome (GRCg7W) with average mapping rate 93.76% (Supplementary Fig. S8) (39). Picard (v. 2.7.1) was used to sort the BAM files, mark duplicates and reorder BAM files (40). Samtools (v. 1.14) was used to index the BAM files (26). GATK (v. 4.2.6.1) was used to split the overlapping intron reads and detect variants (41). The CombineGVCFs function was then used to jointly genotype all these samples into one GVCF per tissue. "GenotypeGVCFs" was used to transfer GVCF to VCF file, and SNPs were extracted using SelectVariants and filtered with "QD < 2.0 || MQ < 40.0 || FS > 60.0 || SOR > 3.0 || MQRankSum < -12.5 || ReadPosRankSum < -8.0". Beagle (v. 4.1) was used to impute the missing genotype using the whole genome sequencing data as the reference panel (32).

eQTL mapping

MatrixEQTL was used to carry out the eQTL analysis for the ovary tissue based on the following model (42),

$$\mathbf{y} = \mathbf{X_2 b_2} + \mathbf{j}\alpha + \mathbf{e}$$

where $\mathbf{y}$ was the TPM from ovary tissue per gene, $\mathbf{b_2}$ was a vector of the fixed effects including genetic group and age effects, $\mathbf{X_2}$ was the corresponding design matrix, $\mathbf{j}$ was a vector with allele counts (coded as 0, 1, and 2 when homozygote for the alternative allele, heterozygote, and homozygote for the reference allele, respectively);

and α is the additive SNP effect. The *cis*-eQTL mapping window was defined from 1 megabase (Mb) upstream/downstream of the transcription start site; all other SNP-expression of gene pairs were defined as trans associated. For both *cis*- and *trans*-eQTL, we applied the *P*-values threshold that corresponds to FDR < 0.01.

Transcriptome-wide association study and Mendelian randomization analysis

We corrected gene expression data for age and genetic group effects, and the residuals were used as the response variable in TWAS analysis. Only *cis*-eQTLs, as identified in the eQTL analysis, were kept as input for the TWAS analysis. With S-PrediXcan (43), we first estimated the weights and covariance matrices of the SNPs within each gene to build a gene expression prediction model. Second, we estimated the associations between predicted gene expression levels and the traits using the GWAS summary statistics and gene expression prediction models. The identified genes were visualized using Rldeogram (44). Subsequently, a MR analysis was done using the software MR-JTI (45) to assess causal inference between gene expression and egg production. This analysis combined LD scores, eQTL-mapping results, and GWAS summary statistics to obtain candidate genes. The input SNPs were pruned such that only relatively independent variants (LD < 0.2) that are associated with the expression of TWAS identified genes were used. LD scores for each independent variant were obtained with GCTA (46). Bonferroni adjustment was applied to correct for multiple testing.

Results

Variance components for cumulative egg number and egg number at different stages

Estimated phenotypic variances were equivalent between models A and AD (Table 1). Phenotypic variance increased with age for cumulative egg number, and increased from EN400 to EN600 for egg number at different stages. For cumulative egg number, the additive variance explained a similar proportion of the phenotypic variance at the early and late laying stage for the two models, 56% and 57% for CEN200, 17% and 19% for CEN300, 13% to 14% for CEN600 and CEN700. In Model AD, the dominance variance accounted for a small proportion of the total phenotypic variance at the early stages (3% for CEN200 and 6% for CEN300), a substantial proportion at the middle stages (15% for CEN400 and 23% for CEN500), and a negligible proportion at late stages, below 0.5%. For egg number at different stages, the additive variance explained a similar proportion of phenotypic variance for both models only at the early stage of laying cycle (16% and 19% for EN300). In Model AD, dominance contributed 9% to 45% to the phenotypic variance for the whole laying period.

Genome-wide association study and candidate variants

The number of significant additive and dominance SNPs detected by Model AD was 3,294 and 2,598, while no significant SNPs were detected by Model A (Supplementary Table S1). The significant SNPs were mainly distributed on chromosomes 1, 2, 3, 6 and 13 (Fig. 2A). Among them, 805 additive SNPs and 360 dominance SNPs were shared between two traits or among multiple traits (Figs. 2B and 2C). For each trait, more than 50% of the significant SNPs were detected as

additive SNPs, except for CEN700 (Fig. 2D). Most of the annotated variants were intron variants, 61.65% based on significant SNPs and 55.69% based on all SNPs (Figs. 2E and 2F). Among the coding consequences, the most abundant variants are synonymous variants with the similar proportion ~0.70 in significant and all SNPs, which were non-neutral (47). The 3' untranslated region (3'UTR) variants were over 3-fold enriched among the significant SNPs compared to all SNPs (Supplementary Table S2), which was demonstrated to play a crucial role in post-transcriptional and translational processes (48, 49).

To further explore the advantages of incorporating dominance into GWAS model to identify the trait-related variants, we examined estimated SNP effects from the different models. The detected additive SNP effects located at chromosome 2 of EN500 from A and AD models were clearly correlated to each other, but effects differed considerably (different scales, Fig. 3A). The additive and dominance SNP effects from Model AD are strongly related to each other (Fig. 3A). These patterns were similar for other chromosomes and traits. For all significant SNPs, most of the additive SNP effects are negative, while most of the dominance effects are positive (Fig. 3C). The additive and dominance SNP effects are in opposite directions for approximately 60% of all SNPs. (Fig. 3D), and this proportion is even higher for significant SNPs (Fig. 3E). Moreover, 251, 20, and 958 SNPs were significant for additive SNP and dominance SNP effects at the same time in traits CEN600, CEN700, and EN500 (Fig. 3F). If we trimmed the significant SNPs based on LD with $r^2 \leq 0.2$ (--indep-pairwise 50 5 0.2), in total 132 independent genomic regions related to egg production traits were identified.

eQTL mapping results

After removing genes with TPM values equal to 0 in all samples, expression data

from 28,126 genes were kept for eQTL mapping analysis. After removing fixed genotypes and genotypes on the sex chromosomes in transcriptome-sequencing animals, 9,030,769 SNPs were kept for subsequent analysis. The eQTL analysis assessed associations between SNPs and gene expression for 508,421,281 *cis*-SNP-expression of gene pairs, and 253,490,987,613 *trans*-SNP-expression of gene pairs (Fig. 4A). At the threshold P < 2.15e-05 (FDR < 0.01), we identified significant 1,093,490 *cis*-SNP-expression of gene pairs, corresponding to 915,638 *cis*-acting SNPs, and 11,471 *cis*-eQTL-associated genes. At the threshold P < 5.90e-07 (FDR<0.01), we identified 14,967,653 *trans*-SNP-expression of gene pairs, corresponding to 2,449,280 *trans*-acting SNPs, and 26,086 *trans*-eQTL-associated genes.

An eQTL can influence the expression of multiple genes, which is denoted as pleiotropy of eQTL (50). Descriptive statistics revealed that 14.90% (136,474/915,638) of *cis*-eQTL and 60.18% (1,473,949/2,449,280) of *trans*-eQTL were associated with the expression of two or more target genes, and 36 *cis*-eQTL and 455,738 *trans*-eQTL were associated with more than ten genes (Fig. 4B). It appeared that the *cis*-eQTL that displayed pleiotropy were distributed in specific regions on chromosomes 2, 19 and 25, which could be regarded as eQTL hotspots (Fig. 4C).

TWAS identify 120 unique genes for egg production traits

We performed TWAS analysis using S-PrediXcan, revealing 312 statistically significant gene expression-egg number associations, comprising 120 genes, whose imputed expression is associated with cumulative egg number and egg number at different stages (Supplementary Table S3). Using the RIdeogram for visualization, we found several genes affecting multiple egg number traits on chromosomes 1, 2, 4, 5, 7, 12, 13, 15, 17, 19, 21 and 26 (Fig. 5A, and Supplementary Fig. S9). There were

shared genes among the traits CEN500, CEN600, CEN700, EN300_500, and EN500 (Fig. 5B). Across different models, 154 significant expression of gene-egg number pairs were found in Model A, 133 significant expression of gene-egg number pairs for additive effects and 25 significant expression of gene-egg number pairs for dominance effects in Model AD (Fig. 5C, and Supplementary Table S3). We further performed a MR causal inference analysis using MR-JTI to prioritize the genes identified by TWAS, and identified 97 candidate genes (Supplementary Table S4).

Multi-omics data analysis for egg production traits

Significant SNPs detected in the GWAS results (Table 2, and Fig. 6A) were associated with 46 genes in *cis*-SNP-gene associations identified in the eQTL analysis (Supplementary Table S5), among which three genes were also detected by TWAS, being *ENSGALG00015008881*, *ENSGALG00015025757*, and *ENSGALG00015027788* (Table 2, and Fig. 6B). Further causal inference with MR analysis showed that the three genes were potential causal genes for egg production (Table 2, Fig. 6F, and Supplementary Table S4). *ENSGALG00015008881* is located on chromosome 1, and the expression level was significantly associated with SNPs 1:74853287 and 1:75008123 (P = 1.47e-6 and P = 1.10e-8, respectively, Table 2 and Fig. 6C), and was associated with EN500 with Z-score 3.19 (corresponding P = 1.44e-03, Table 2, Fig. 6B, and Supplementary Table S3). The expression level of *ENSGALG00015027788* was associated with SNP 17:644033 of trait EN500 (P = 1.27e-05, Table 2, and Fig. 6D), and was associated with EN500 with Z-score -2.78 (corresponding P = 5.47e-03, Table 2, Fig. 6B, and Supplementary Table S3). Similarly, the expression level of *ENSGALG00015025757* was associated with 17 SNPs locating on chromosome 21 (P-values ranged from 4.63e-07 to 1.52e-05, Table 2, and Fig. 6E), and was associated with EN500 with Z-score 3.13 (corresponding P =

1.74e-03, Table 2, Fig. 6B, and Supplementary Table S3).

In addition to candidate genes, we also pinpointed several candidate SNPs. For SNP 21:1055253, reference allele C and alternative allele T, egg number of genotype TT was smaller than that of genotype CC, and the expression of *ENSGALG00015025757* for genotype TT was the highest (Fig. 7). For SNP 1:74853287, the egg number of genotype GG at each stage was smaller than the genotype CG (Supplementary Fig. S10A), and the expression of *ENSGALG00015008881* for genotype CC was lower than the genotype GG (Supplementary Fig. S10B). For SNP 17:644033, the egg number of genotype TT at each stage was smaller than the genotype TG (Supplementary Fig. S10C), and the expression of *ENSGALG00015027788* for genotype GG was the highest (Supplementary Fig. S10D).

Implications for heterosis

Observed heterosis from the predicted mean phenotypes of each genetic group increased across ages for cumulative egg number from 1.04% to 11.51%, except for CEN200. For egg number at different stages, heterosis increased from -3.22% to 29.48% (Table 3). Leveraging Model AD enables the calculation of SNP ratios to assess the relative importance of dominance compared to additive SNP effects. The ratios for most of the significant SNPs were higher than 0.8 (Fig. 8A red dash line), and smaller than 1.2 (Fig. 8A blue dash line), suggesting complete-dominance. Across traits, we observed a positive correlation coefficient of 0.45 (P = 0.31) between SNP ratios and heterosis (Fig. 8A). Similarly, a positive and significant correlation of 0.72 was observed between the sum of dy^2 across all SNPs and heterosis, with P = 0.0053 (Fig. 8B). Finally, a positive but insignificant correlation of 0.60 was observed for significant SNPs between the sum of dy^2 and the heterosis, with P = 0.15 (Fig. 8B).

Discussion

In the current study, by incorporating dominance SNP effects into the GWAS model and using whole-genome sequencing data for a complete double-crossed hybrid population of 1,004 chickens, we successfully identified genetic variants related to egg production traits. Egg production remains the most important traits for laying hens, despite the recent expansion of breeding goals to include increasingly more health and welfare related traits (51). In total, the eQTL-mapping analysis identified 1,093,490 *cis*-SNPs and 14,967,653 *trans*-SNPs, and TWAS analysis identified 120 genes. By carrying out the multi-omics data analysis, three novel candidate causal genes for egg production traits were identified. Moreover, observed heterosis positively related to the heterosis predicted based on the estimated dominance SNP effects and allele frequencies, and average ratios of dominance to additive effects.

The traditional GWAS analysis in livestock normally focus on estimating additive effects, while non-additive effects, such as dominance, are frequently overlooked. When non-additive effects actually exist but are not modelled, they may end up partly in the residual effect and partly in the additive effect, leading to bias in the estimation of additive effects (52). Additionally, incorporating dominance SNP effects is justified by the fact that a substantial portion of genetic variance, especially in mixed populations of purebred and crossbred animals, is likely explained by non-additive effects (53). In the current study, with only additive SNP effects in Model A, no significant SNPs were found, while by adding dominance SNP effects in Model AD, several signals appeared across traits (Supplementary Fig. S11). This finding agrees with previous research that emphasizes the role of dominance in enhancing the detection of associations (14, 54), and in particular when using crossbred data (8). To

further explore the advantages of incorporating dominance into GWAS model, we analyzed SNP effects in both models. Although additive SNP effects were correlated between the models, their scales differ. In Model A, we suspected that opposing additive and dominance SNP effects can potentially cancel out. In contrast, Model AD distinguished between additive and dominance SNP effects, leading to the identification of more variants in our population consisting of purebred and crossbred animals. We observed generally positive dominance SNP effects for significant SNPs, consistent with the observation that the heterozygote is the favorable genotype in most cases (Supplementary Fig. S12). The sign of additive SNP effects depends on which allele is counted to derive the genotypes used in the model, whereas the sign of the dominance SNP effects is not dependent on the allele coding. To maintain consistency, in the current study, we derived genotypes by counting the alternate allele. Irrespective of the sign of SNP effects, the magnitudes of additive and dominance SNP effects are not independent, in line with previous finding (55).

Another reason for identifying a larger number of trait-related variants is the use of whole-genome sequencing data. Most GWAS studies in chickens have relied on genotype data obtained from SNP chips, which include only a fraction of all the variants segregating in the whole chicken genome. In contrast, whole-genome sequencing data encompass nearly all genomic variants, which can enhance the effectiveness of GWAS in identifying causal mutations for quantitative traits (56). Taken together, incorporating dominance into the GWAS model proved beneficial for identifying genetic variants for egg production traits of layers in our full diallel cross with purebred and crossbred animals with whole-genome sequencing data.

In the current study, integrating GWAS, eQTL-mapping analysis, and TWAS, we ultimately discovered three novel genes with potential causal roles in influencing egg

production. *ENSGALG00015008881* is a novel gene without annotation and locates upstream of the gene *ANO2* (Anoctamin 2). Reviewing the annotations in NCBI database, we found this gene is part of *ANO2*. *ANO2* belongs to a family of calcium-activated chloride channels, encoding the protein TMEM16B (transmembrane protein 16B), which was regarded as a critical mediator of activity at the photoreceptor terminals. (57). The calcium signaling pathway associated with this gene was essential for ovarian follicle development and ovulation in chickens, thus influencing egg production performance. *ENSGALG00015025757*, annotated as *ARHGEF16* (Rho Guanine Nucleotide Exchange Factor 16), is reported to participate in regulating the activation of Rho-like GTPases (58). There are only few studies on the function of *ARHGEF* gene family in poultry, but Dunn et al. reported differential expression of *ARHGEF* in the shell gland tissue between hens of 12 and 25 weeks of age (59), indicating the potential function in regulating the egg producing process. Finally, *ENSGALG00015027788*, annotated as the *NDOR1* (NADPH Dependent Diflavin Oxidoreductase 1), is involved in the NADPH-hemoprotein reductase activity, flavin mononucleotide binding, and oxidoreductase activity (60). Oxidative stress could impact egg quality and reproductive performance (61), indicating the potential role of *NDOR1* on egg production. This integrated analysis of genomic and transcriptome data helps to identify trait-related genetic variants and genes, showing that multi-omics data can contribute to deciphering genetic mechanisms underlying egg production by establishing connections between genetic variants, gene expression, and egg number.

In addition to identifying candidate genetic variants related to egg production traits, we also explored implications regarding heterosis utilizing estimated dominance SNP effects, given that chicken is one of the most well-known animals where hybrid vigor,

or heterosis, is leveraged in commercial populations. Following the methods described previously (8, 21), we calculated observed heterosis from predicted means for genetic groups of egg production traits, and assessed the extent of dominance effects per SNP through SNP ratios. Ratios of significant SNP identified by GWAS generally ranged from 0.8 to 1.2, with the mean value 1.00 (Supplementary Table S6), suggesting a complete-dominance SNP pattern (8). Based on these results, complete-dominance is expected to be the predominant pattern in egg production heterosis. We did not find other studies in animals attempted to investigated this, but this observation aligns with previous reports in plants (62, 63). It should be noted that in our study the observed correlations between SNP ratio and heterosis could be affected by the fact that the traits and thus also the significant SNPs across traits are highly related, or by the limited number of traits considered. Nevertheless, we did observe a positive correlation between SNP ratio and heterosis, consistent with the theory and the expectation, stating that with increasing extent of dominance compared to additive effects, a larger amount of heterosis is expected. Moreover, we observed a lower correlation between heterosis and the SNP ratios for all compared to only the significantly detected SNPs (Supplementary Fig. S13).

In addition to the SNP ratios, we considered the quantitative genetics theory that the amount of heterosis depends on dy^2, i.e. the product of the dominance effects multiplied by the squared difference in allele frequency (17). We observed a positive and significant correlation between the sum of dy^2 and heterosis for all SNPs, but an insignificant correlation for significant SNPs. Given the similar correlation of 0.72 for all SNPs and 0.60 for significant SNPs, we argue that the limited number of significant SNPs still had an important impact on phenotypes. In line with this, Amuzu-Aweh et al. reported an accuracy of ~0.5 for predicting heterosis using the

genome-wide squared difference in allele frequency between parental pure lines (64).

Conclusion

In the current study, we identified 5,982 SNPs and three candidate genes for egg production traits by analyzing 1,004 fully sequenced layers. These results suggest that incorporating dominance into the GWAS model not only help to detect the variants for egg production traits of layers in a mixed population with purebred and crossbred animals, but also demonstrates that traits with higher heterosis tended to be more affected by genes with dominant mode of action. Moreover, multi-omics data allows to contribute to deciphering genetic mechanisms underlying egg production by establishing connections between genetic variants, gene expression, and egg number.

Additional Files

Supplementary Fig. S1. Mapping quality of 1,004 whole-genome sequenced animals

Supplementary Fig. S2. Scatter plot for pedigree and genome coefficients for crossbreds

Supplementary Fig. S3. Scatter plot for pedigree and genome coefficients for crossbreds after removing conflict animals

Supplementary Fig. S4. Scatter plot for pedigree and genome coefficients for WW

Supplementary Fig. S5. Scatter plot for pedigree and genome coefficients for WW after removing conflict animals

Supplementary Fig. S6. Scatter plot for pedigree and genome coefficients for YY

Supplementary Fig. S7. Scatter plot for pedigree and genome coefficients for YY after removing conflict animals

Supplementary Fig. S8. Mapping quality of transcriptome data

Supplementary Fig. S9. Genes identified by TWAS analysis

Supplementary Fig. S10. The correlations between candidate variants and phenotype, and expression of candidate genes

Supplementary Fig. S11. QQplots across traits

Supplementary Fig. S12. Counts of favorable genotype for significant SNPs

Supplementary Fig. S13. Correlation between SNP ratios of all SNPs and heterosis

Supplementary Table S1. Significant SNPs identified by GWAS

Supplementary Table S2. Annotation of significant and all SNPs

Supplementary Table S3. Significant genes identified by TWAS

Supplementary Table S4. MR analyze for candidate genes identified by TWAS

Supplementary Table S5. Expression level of 46 genes associated with significant SNPs identified by GWAS

Supplementary Table S6. Ratio values of significant SNPs

Data Availability

Raw sequence data have been submitted to the NGDC (National Genomics Data Center) database under accession number PRJCA031434.

List of abbreviations

SNP: single nucleotide polymorphisms; GWAS: genome-wide association study; TWAS: transcriptome-wide association study; eQTL: expression quantitative trait loci; QTL: quantitative trait loci; MR: Mendelian randomization; dy^2: the sum of the

dominance effects multiplied by the squared difference in allele frequency between the parental lines; YY: Beijing-You chickens; WW: White Leghorn chickens; WY: offspring of a cross between White Leghorn as the sire line and Beijing-You as the dam line; YW: offspring of a cross between Beijing-You as the sire line and White Leghorn as the dam line; CEN200: cumulative egg number till 200 days of age; CEN300: cumulative egg number till 300 days of age; CEN400: cumulative egg number till 400 days of age; CEN500: cumulative egg number till 500 days of age; CEN600: cumulative egg number till 600 days of age; CEN700: cumulative egg number till 700 days of age; EN300: egg number from 200 to 300 days of age; EN400: egg number from 300 to 400 days of age; EN500: egg number from 400 to 500 days of age; EN600: egg number from 500 to 600 days of age; EN700: egg number from 600 to 700 days of age; EN300_500: egg number from 300 to 500 days of age; EN500_700: egg number from 500 to 700 days of age; Model A: additive model; Model AD: additive-dominance model; FDR: false discovery rate; VEP: variant effect predictor; TPM: transcripts per kilobase per million mapped reads; Mb: megabase; LD: linkage disequilibrium; 3'UTR: 3' untranslated region; *ANO2*: Anoctamin 2; TMEM16B: transmembrane protein 16B; *ARHGEF*: Rho Guanine Nucleotide Exchange Factor; *NDOR1*: NADPH Dependent Diflavin Oxidoreductase 1.

Declarations

Ethical approval

The study was approved by the Animal Care and Use Committee of the Institute of Animal Science, Chinese Academy of Agricultural Sciences (No. IAS2021-48), where the experiments were conducted. All experiments were performed in accordance with the relevant guidelines and regulations set by Ministry of Agriculture and Rural

Affairs of the People’s Republic of China.

Consent for publication

Not applicable

Competing interests

The author(s) declare that they have no competing interests.

Funding

This work was supported by the National Natural Science Foundation of China (32172721), Chinese Agricultural Research System (CARS-40), and the Agricultural Science and Technology Innovation Program (ASTIP-2021-IAS-16).

Authors’ contributions

J.C. and Y.S. conceived the initial study design. H.B., M.P.L.C., Y.S., and J.C., discussed, specified the final study design and supervised the work, A.N., Y.L., and J.Y. collected the phenotype data. A.N. performed the bioinformatic analysis, and wrote the first version of the manuscript. Y.L., and J.Y. contributed to statistical analyses. A.N., H.B., M.P.L.C., Y.L., Y.S. and J.C. provided valuable insights throughout the writing process. All authors read and approved the final manuscript.

Acknowledgements

The authors extend their gratitude to Chao Chen (Institute of Animal Science, Chinese Academy of Agricultural Sciences, Beijing, China) for his assistance with raising animals. The authors also acknowledge Martijn Derks and Carolina Barros (Animal Breeding and Genomics Group, Wageningen University and Research, the Netherlands) for their help in SNP calling and gene annotations.

Reference

1. Statista. Global egg production from 1990 to 2022 2024 [Available from: https://www.statista.com/statistics/263972/egg-production-worldwide-since-1990/.

2. Bain MM, Nys Y, Dunn IC. Increasing persistency in lay and stabilising egg quality in longer laying cycles. What are the challenges? Br Poult Sci. 2016;57(3):330-8.

3. Uffelmann E, Huang QQ, Munung NS, de Vries J, Okada Y, Martin AR, et al. Genome-wide association studies. Nature Reviews Methods Primers. 2021;1(1):59.

4. Cai W, Zhang Y, Chang T, Wang Z, Zhu B, Chen Y, et al. The eQTL colocalization and transcriptome-wide association study identify potentially causal genes responsible for economic traits in Simmental beef cattle. Journal of Animal Science and Biotechnology. 2023;14(1):78.

5. Liu X, Zhang J, Xiong X, Chen C, Xing Y, Duan Y, et al. An Integrative Analysis of Transcriptome and GWAS Data to Identify Potential Candidate Genes Influencing Meat Quality Traits in Pigs. Frontiers in Genetics. 2021;12:748070.

6. Teng J, Gao Y, Yin H, Bai Z, Liu S, Zeng H, et al. A compendium of genetic regulatory effects across pig tissues. Nat Genet. 2024;56(1):112-23.

7. Guan D, Bai Z, Zhu X, Zhong C, Hou Y, Lan F, et al. The ChickenGTEx pilot analysis: a reference of regulatory variants across 28 chicken tissues. bioRxiv. 2023;06.

8. Cui L, Yang B, Xiao S, Gao J, Baud A, Graham D, et al. Dominance is common in mammals and is associated with trans-acting gene expression and alternative splicing. Genome Biol. 2023;24(1):215.

9. Liu Y, Xu L, Wang Z, Xu L, Chen Y, Zhang L, et al. Genomic Prediction and Association Analysis with Models Including Dominance Effects for Important Traits

in Chinese Simmental Beef Cattle. Animals (Basel). 2019;9(12):1055.

10. Ebrahimi K, Dashab GR, Faraji-Arough H, Rokouei M. Estimation of additive and non-additive genetic variances of body weight in crossbreed populations of the Japanese quail. Poult Sci. 2019;98(1):46-55.

11. Alves K, Brito LF, Baes CF, Sargolzaei M, Robinson JAB, Schenkel FS. Estimation of additive and non‐additive genetic effects for fertility and reproduction traits in North American Holstein cattle using genomic information. J Anim Breed Genet. 2020;137(3):316-30.

12. Joshi R, Meuwissen THE, Woolliams JA, Gjoen HM. Genomic dissection of maternal, additive and non-additive genetic effects for growth and carcass traits in Nile tilapia. Genet Sel Evol. 2020;52(1):1.

13. Amuzu‐Aweh EN. Genomics of heterosis and egg production in White Leghorns: Wageningen University and Research; 2020.

14. Nagai R, Kinukawa M, Watanabe T, Ogino A, Kurogi K, Adachi K, et al. Genome-wide detection of non-additive quantitative trait loci for semen production traits in beef and dairy bulls. Animal. 2022;16(3):100472.

15. Estrada-Reyes ZM, Rae DO, Mateescu RG. Genome-wide scan reveals important additive and non-additive genetic effects associated with resistance to Haemonchus contortus in Florida Native sheep. Int J Parasitol. 2021;51(7):535-43.

16. Tarsani E, Kranis A, Maniatis G, Avendano S, Hager-Theodorides AL, Kominakis A. Deciphering the mode of action and position of genetic variants impacting on egg number in broiler breeders. BMC Genomics. 2020;21(1):512.

17. Falconer DS. Introduction to quantitative genetics: Pearson Education India; 1996.

18. Li D, Huang Z, Song S, Xin Y, Mao D, Lv Q, et al. Integrated analysis of

phenome, genome, and transcriptome of hybrid rice uncovered multiple heterosis-related loci for yield increase. Proc Natl Acad Sci U S A. 2016;113(41):E6026-E35.

19. Wang B, Hou M, Shi J, Ku L, Song W, Li C, et al. De novo genome assembly and analyses of 12 founder inbred lines provide insights into maize heterosis. Nat Genet. 2023;55(2):312-23.

20. Ye J, Liang H, Zhao X, Li N, Song D, Zhan J, et al. A systematic dissection in oilseed rape provides insights into the genetic architecture and molecular mechanism of yield heterosis. Plant Biotechnol J. 2023;21(7):1479-95.

21. Ni A, Calus MPL, Bovenhuis H, Yuan J, Wang Y, Sun Y, et al. Genetic parameters, reciprocal cross differences, and age-related heterosis of egg-laying performance in chickens. Genet Sel Evol. 2023;55(1):87.

22. Andrews S. FastQC: a quality control tool for high throughput sequence data. Cambridge, United Kingdom; 2010.

23. Bolger AM, Lohse M, Usadel B. Trimmomatic: a flexible trimmer for Illumina sequence data. Bioinformatics. 2014;30(15):2114-20.

24. Li H, Durbin R. Fast and accurate short read alignment with Burrows–Wheeler transform. bioinformatics. 2009;25(14):1754-60.

25. Faust GG, Hall IM. SAMBLASTER: fast duplicate marking and structural variant read extraction. Bioinformatics. 2014;30(17):2503-5.

26. Li H, Handsaker B, Wysoker A, Fennell T, Ruan J, Homer N, et al. The sequence alignment/map format and SAMtools. bioinformatics. 2009;25(16):2078-9.

27. Rhie A, McCarthy SA, Fedrigo O, Damas J, Formenti G, Koren S, et al. Towards complete and error-free genome assemblies of all vertebrate species. Nature. 2021;592(7856):737-46.

28. Garrison E, Marth G. Haplotype-based variant detection from short-read

sequencing. arXiv preprint arXiv:12073907. 2012.

29. Bonfield JK, Marshall J, Danecek P, Li H, Ohan V, Whitwham A, et al. HTSlib: C library for reading/writing high-throughput sequencing data. Gigascience. 2021;10(2):giab007.

30. Okonechnikov K, Conesa A, García-Alcalde F. Qualimap 2: advanced multi-sample quality control for high-throughput sequencing data. Bioinformatics. 2016;32(2):292-4.

31. Purcell S, Neale B, Todd-Brown K, Thomas L, Ferreira MA, Bender D, et al. PLINK: a tool set for whole-genome association and population-based linkage analyses. The American journal of human genetics. 2007;81(3):559-75.

32. Browning SR, Browning BL. Rapid and accurate haplotype phasing and missing-data inference for whole-genome association studies by use of localized haplotype clustering. The American Journal of Human Genetics. 2007;81(5):1084-97.

33. Meyer K. WOMBAT—A tool for mixed model analyses in quantitative genetics by restricted maximum likelihood (REML). J Zhejiang Univ Sci B. 2007;8(11):815-21.

34. VanRaden PM. Efficient methods to compute genomic predictions. J Dairy Sci. 2008;91(11):4414-23.

35. Vitezica ZG, Varona L, Legarra A. On the additive and dominant variance and covariance of individuals within the genomic selection scope. Genetics. 2013;195(4):1223-30.

36. Yin L, Zhang H, Tang Z, Xu J, Yin D, Zhang Z, et al. rMVP: a memory-efficient, visualization-enhanced, and parallel-accelerated tool for genome-wide association study. Genomics, Proteomics and Bioinformatics. 2021;19(4):619-28.

37. McLaren W, Gil L, Hunt SE, Riat HS, Ritchie GR, Thormann A, et al. The

ensembl variant effect predictor. Genome Biol. 2016;17:1-14.

38. Yuan J, Zhao J, Sun Y, Wang Y, Li Y, Ni A, et al. The mRNA-lncRNA landscape of multiple tissues uncovers key regulators and molecular pathways that underlie heterosis for feed intake and efficiency in laying chickens. Genet Sel Evol. 2023;55(1):69.

39. Dobin A, Davis CA, Schlesinger F, Drenkow J, Zaleski C, Jha S, et al. STAR: ultrafast universal RNA-seq aligner. Bioinformatics. 2013;29(1):15-21.

40. Institute B. Picard: a set of Java command line tools for manipulating high-throughput sequencing data (HTS) data and formats.: GitHub Repository; 2020.

41. Van der Auwera GA, O'Connor BD. Genomics in the cloud: using Docker, GATK, and WDL in Terra: O'Reilly Media; 2020.

42. Shabalin AA. Matrix eQTL: ultra fast eQTL analysis via large matrix operations. Bioinformatics. 2012;28(10):1353-8.

43. Barbeira AN, Dickinson SP, Bonazzola R, Zheng J, Wheeler HE, Torres JM, et al. Exploring the phenotypic consequences of tissue specific gene expression variation inferred from GWAS summary statistics. Nature Communications. 2018;9(1):1825.

44. Hao Z, Lv D, Ge Y, Shi J, Weijers D, Yu G, et al. RIdeogram: drawing SVG graphics to visualize and map genome-wide data on the idiograms. PeerJ Computer Science. 2020;6:e251.

45. Zhou D, Jiang Y, Zhong X, Cox NJ, Liu C, Gamazon ER. A unified framework for joint-tissue transcriptome-wide association and Mendelian randomization analysis. Nat Genet. 2020;52(11):1239-46.

46. Yang J, Lee SH, Goddard ME, Visscher PM. GCTA: a tool for genome-wide complex trait analysis. Am J Hum Genet. 2011;88(1):76-82.

47. Shen X, Song S, Li C, Zhang J. Synonymous mutations in representative yeast

genes are mostly strongly non-neutral. Nature. 2022;606(7915):725-31.

48. Griesemer D, Xue JR, Reilly SK, Ulirsch JC, Kukreja K, Davis JR, et al. Genome-wide functional screen of 3'UTR variants uncovers causal variants for human disease and evolution. Cell. 2021;184(20):5247-60.

49. Mayr C, Bartel DP. Widespread Shortening of 3′UTRs by Alternative Cleavage and Polyadenylation Activates Oncogenes in Cancer Cells. Cell. 2009;138(4):673-84.

50. Tian J, Keller MP, Broman AT, Kendziorski C, Yandell BS, Attie AD, et al. The Dissection of Expression Quantitative Trait Locus Hotspots. Genetics. 2016;202(4):1563-74.

51. Wolc A. Poultry breeding programs in XXI century and beyond. 65-LECIE KOMITETU NAUK ZOOTECHNICZNYCH I AKWAKULTURY PAN. 2022:83.

52. Duenk P, Calus MPL, Wientjes YCJ, Bijma P. Benefits of Dominance over Additive Models for the Estimation of Average Effects in the Presence of Dominance. G3 (Bethesda). 2017;7(10):3405-14.

53. Bouvet JM, Makouanzi G, Cros D, Vigneron P. Modeling additive and non-additive effects in a hybrid population using genome-wide genotyping: prediction accuracy implications. Heredity (Edinb). 2016;116(2):146-57.

54. Bolormaa S, Pryce JE, Zhang Y, Reverter A, Barendse W, Hayes BJ, et al. Non-additive genetic variation in growth, carcass and fertility traits of beef cattle. Genet Sel Evol. 2015;47(1):26.

55. Bennewitz J, Meuwissen TH. The distribution of QTL additive and dominance effects in porcine F2 crosses. J Anim Breed Genet. 2010;127(3):171-9.

56. Heidaritabar M, Bink M, Dervishi E, Charagu P, Huisman A, Plastow GS. Genome-wide association studies for additive and dominance effects for body

composition traits in commercial crossbred Pietrain pigs. J Anim Breed Genet. 2023;140(4):413-30.

57. Stohr H, Heisig JB, Benz PM, Schoberl S, Milenkovic VM, Strauss O, et al. TMEM16B, a novel protein with calcium-dependent chloride channel activity, associates with a presynaptic protein complex in photoreceptor terminals. J Neurosci. 2009;29(21):6809-18.

58. Rossman KL, Der CJ, Sondek J. GEF means go: turning on RHO GTPases with guanine nucleotide-exchange factors. Nature reviews Molecular cell biology. 2005;6(2):167-80.

59. Dunn IC, Wilson PW, Lu Z, Bain MM, Crossan CL, Talbot RT, et al. New hypotheses on the function of the avian shell gland derived from microarray analysis comparing tissue from juvenile and sexually mature hens. Gen Comp Endocrinol. 2009;163(1-2):225-32.

60. Finn RD, Wilkie M, Smith G, Paine MJ. Identification of a functionally impaired allele of human novel oxidoreductase 1 (NDOR1), NDOR1*1. Pharmacogenet Genomics. 2005;15(6):381-6.

61. Ding X, Cai C, Jia R, Bai S, Zeng Q, Mao X, et al. Dietary resveratrol improved production performance, egg quality, and intestinal health of laying hens under oxidative stress. Poult Sci. 2022;101(6):101886.

62. Liu H, Wang Q, Chen M, Ding Y, Yang X, Liu J, et al. Genome-wide identification and analysis of heterotic loci in three maize hybrids. Plant Biotechnol J. 2020;18(1):185-94.

63. Yang M, Wang X, Ren D, Huang H, Xu M, He G, et al. Genomic architecture of biomass heterosis in Arabidopsis. Proc Natl Acad Sci U S A. 2017;114(30):8101-6.

64. Amuzu-Aweh EN, Bijma P, Kinghorn BP, Vereijken A, Visscher J, van

Arendonk JA, et al. Prediction of heterosis using genome-wide SNP-marker data: application to egg production traits in white Leghorn crosses. Heredity (Edinb). 2013;111(6):530-8.

Table

Click here to access/download;Table;Table_AN_20241017.docx

Table 1. Estimates of variances for cumulative egg number and egg number at different stages

Trait	Model A		Model AD			
	σ_a^2/σ_p^2 (%)	σ_p^2	σ_a^2/σ_p^2 (%)	σ_d^2/σ_p^2 (%)	σ_G^2/σ_p^2 (%)	σ_p^2
CEN200	57	66	56	3	59	66
CEN300	19	180	17	6	23	180
CEN400	16	426	12	15	27	431
CEN500	11	1158	7	23	29	1188
CEN600	13	2589	13	1e-2	13	2589
CEN700	14	4400	14	3e-1	14	4401
EN300	19	104	16	9	25	104
EN400	25	93	16	31	48	96
EN500	12	315	2	45	47	331
EN600	17	547	13	14	26	552
EN700	18	554	9	23	32	560
EN300_500	13	595	8	27	35	615
EN500_700	21	1732	15	19	34	1749

Model A: model with only additive genetic effects, Model AD: model with additive and dominance effects; CENX: cumulative egg number till X days of age, ENX: egg number in 100 days interval till X days of age; EN300_500: egg number between 300 and 500 days of age; EN500_700: egg number between 500 and 700 days of age.

Standard errors for phenotypic variances ranged from 4 to 274 for Model A, and from 4 to 272 for Model AD. Standard errors for additive variances ranged from 6 to 360 for Model A, and from 6 to 373 for Model AD. Standard errors for dominance variances ranged from 5 to 507 for Model AD.

Table 2. Genes associated with egg production traits in additive-dominance model for additive SNP effects

Ensembl ID	SNP	Trait	GWAS_pvalue	eQTL_pvlaue	Z-score	MR_beta	MR_95%_CI
ENSGALG00015008881	1:74853287	EN500	1.41E-06	1.47E-06	3.19	0.50	(0.22, 0.67)
	1:75008123	EN500	2.02E-06	1.10E-08			
ENSGALG00015025757	21:875770	EN500	5.48E-10	8.85E-06	3.13	0.30	(0.08, 0.47)
	21:876167	EN500	2.12E-09	1.52E-05			
	21:934741	EN500	5.60E-10	7.39E-06			
	21:938545	EN500	5.60E-10	3.32E-06			
	21:939370	EN500	5.60E-10	2.09E-06			
	21:940575	EN500	5.60E-10	2.09E-06			
	21:947716	EN500	4.10E-10	9.01E-06			
	21:955248	EN500	5.60E-10	8.30E-06			
	21:959037	EN500	5.60E-10	1.18E-06			
	21:959428	EN500	5.68E-10	2.09E-06			
	21:960286	EN500	5.60E-10	2.09E-06			
	21:960523	EN500	1.59E-09	1.92E-06			
	21:961892	EN500	5.86E-08	4.63E-07			
	21:1043126	EN500	2.52E-06	1.48E-05			
	21:1054536	EN500	6.65E-07	2.46E-06			
	21:1055241	EN500	2.52E-06	6.37E-07			
	21:1055253	EN500	2.52E-06	1.05E-05			
ENSGALG00015027788	17:532594	EN300_500	2.03E-07	7.34E-06	-3.5	-0.31	(-0.36, -0.14)
	17:540449	EN300_500	7.97E-09	1.20E-06			
	17:669726	EN300_500	1.43E-07	2.06E-05			
	17:644033	EN300_500	8.99E-09	1.27E-05			
		EN500	5.64E-07		-2.78	-0.27	(-0.44, -0.17)

Table 3. Predicted egg production traits for each genetic group and estimates of heterosis for the crossbreds

Trait	WW	YY	WY	YW	Heterosis (%)
CEN200	38.06	14.67	28.604	14.87	12.52
CEN300	126.10	93.37	110.72	93.37	1.04
CEN400	198.80	143.47	178.49	143.5	4.63
CEN500	269.60	194.08	247.46	194.02	4.77
CEN600	320.30	229.35	304.43	229.21	8.87
CEN700	378.20	251.50	363.34	251	11.51
EN300	88.81	77.54	82.656	77.39	-3.22
EN400	71.25	50.59	66.927	50.54	8.13
EN500	68.68	47.72	68.4657	47.69	15.44
EN600	48.77	33.54	56.161	33.08	20.33
EN700	56.36	21.01	57.0768	20.59	29.48
EN300_500	143.50	99.93	136.089	99.92	11.05
EN500_700	104.80	54.36	113.232	53.28	23.13

CENX: cumulative egg number till X days of age, ENX: egg number in 100 days interval till X days of age; EN300_500: egg number between 300 and 500 days of age; EN500_700: egg number between 500 and 700 days of age.

Heterosis (%): the average percentage of performance of crossbreds being better than the average performance of the two parental lines.

 Click here to access/download;Figure;Figure_AN_20241017.docx

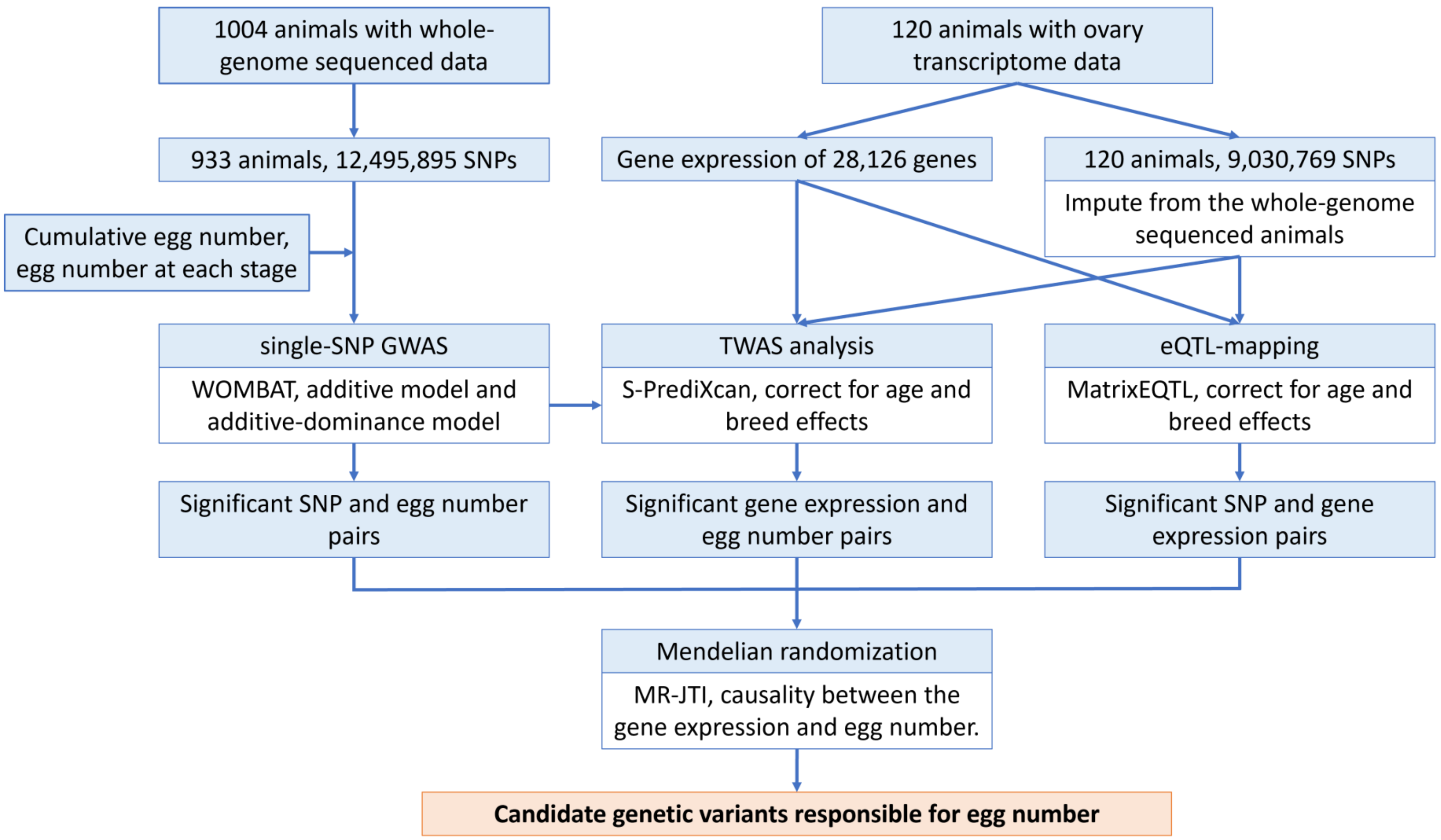

Fig. 1. Framework for identification of SNPs and genes for egg production traits using multi-omics data

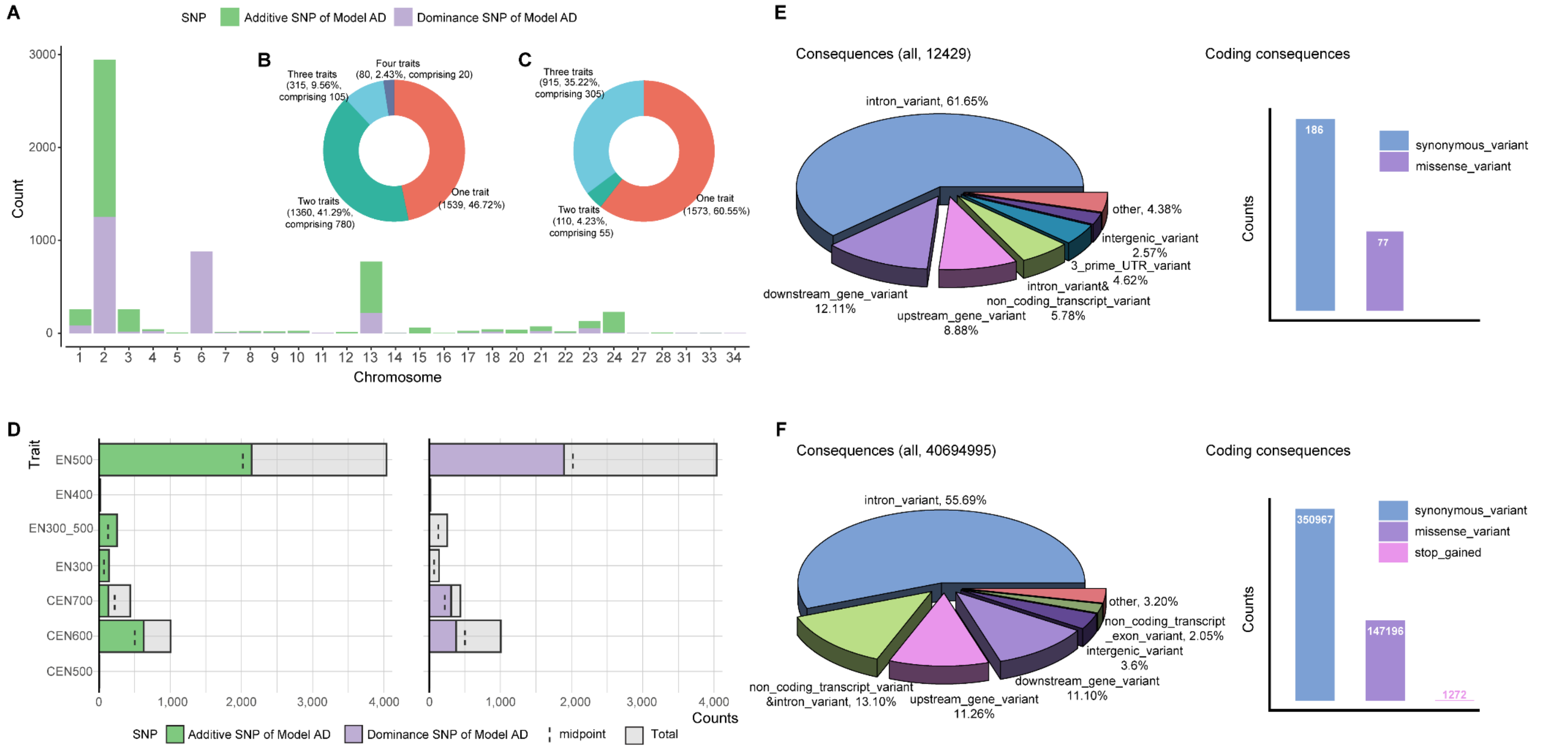

Fig. 2. Distribution of significant SNPs and appearances of significant SNPs. **(A)** Distribution of significant SNPs across chromosomes **(B)** Number and percentage of significant additive SNPs in Model AD for different appearances **(C)** Number and percentage of significant dominance SNPs in Model AD for different appearances. **(D)** Distribution of significant SNPs across traits, filtered by SNP effects. **(E)** Variant Effect Predictor (VEP)

annotations of significant SNPs. **(F)** VEP annotations of all SNPs.

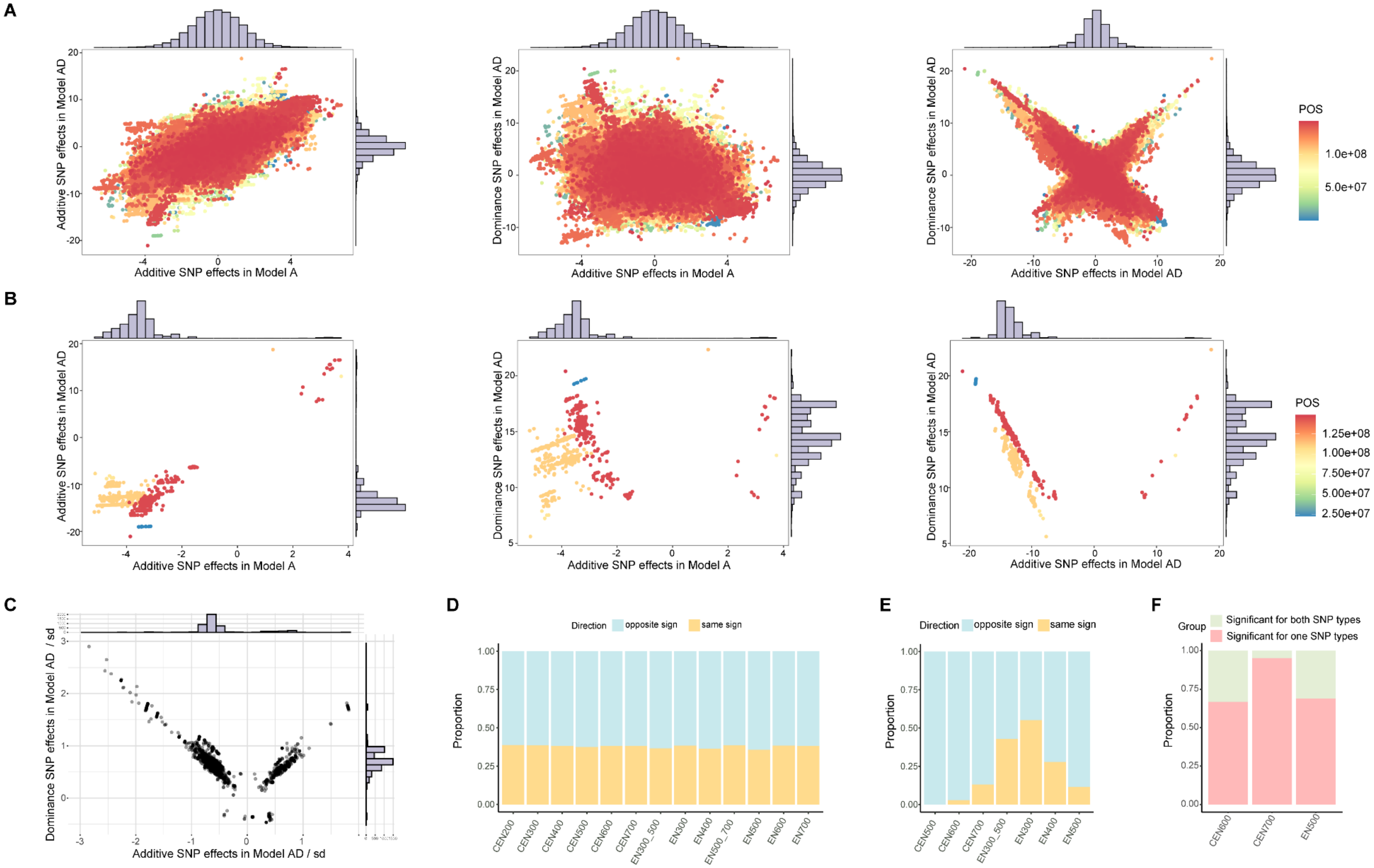
A
Additive SNP effects in Model AD
Additive SNP effects in Model A
Dominance SNP effects in Model AD
Additive SNP effects in Model A
Dominance SNP effects in Model AD
Additive SNP effects in Model AD
POS
1.0e+08
5.0e+07
B
Additive SNP effects in Model AD
Additive SNP effects in Model A
Dominance SNP effects in Model AD
Additive SNP effects in Model A
Dominance SNP effects in Model AD
Additive SNP effects in Model AD
POS
1.25e+08
1.00e+08
7.50e+07
5.00e+07
2.50e+07
C
Dominance SNP effects in Model AD / sd
Additive SNP effects in Model AD / sd
D
Direction
opposite sign
same sign
Proportion
CEN200
CEN300
CEN400
CEN500
CEN600
CEN700
EN300_500
EN300
EN400
EN500_700
EN500
EN600
EN700
E
Direction
opposite sign
same sign
Proportion
CEN500
CEN600
CEN700
EN300_500
EN300
EN400
EN500
F
Group
Significant for both SNP types
Significant for one SNP types
Proportion
CEN600
CEN700
EN500

Fig. 3. SNP effects of additive and dominance SNPs from additive model and additive-dominance model. **(A)** SNP effects for all SNPs for chromosome 2 of EN500. **(B)** SNP effects for significant SNPs for chromosome 2 of EN500. similar **(C)** Distribution of SNP effects standardized with phenotypic standard deviation for all significant SNPs. **(D)** Direction of SNP effects for all SNPs in additive-dominance model. **(E)** Direction of SNP effects for significant SNPs in additive-dominance model. **(F)** Proportion that SNPs be significant for both SNP types to all significant SNPs.

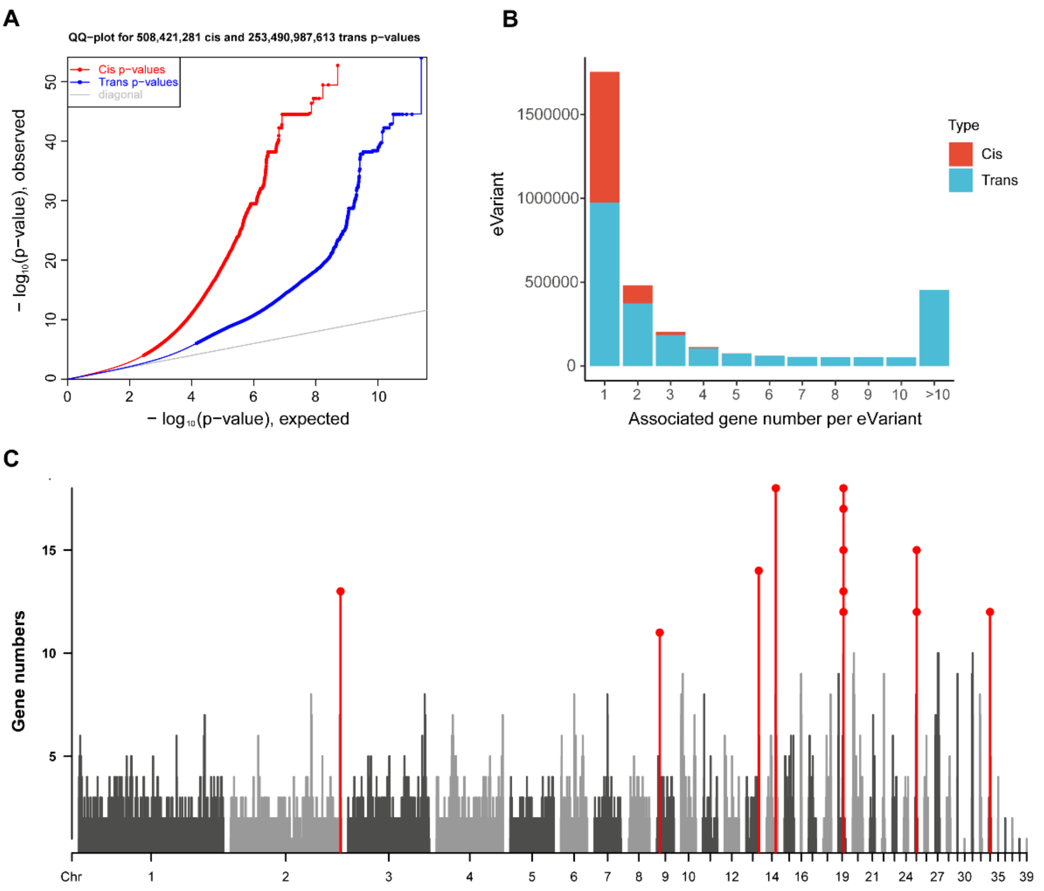

Fig. 4. eQTL profiles. **(A)** QQ-plot of -Log10(p value) of eQTL analysis. "Local p-values" represent the statistical p values for cis-eQTL and "Distant p-values" represent the statistical p values for trans-eQTL. **(B)** Analysis of eQTL pleiotropy. The X-axis of the histogram represents different groups that were classified according to the associated gene numbers per eVariant, and the Y-axis represents the eQTL count for each group. cis-eQTL in red and trans-eQTL in blue. **(C)** Distribution of eQTL hotspot for cis-eQTLs. The X-axis represents the chromosome distribution of eQTL, the Y-axis indicates the number of genes associated with each eQTL. If the number of associated genes is greater than 10, it is shown in red.

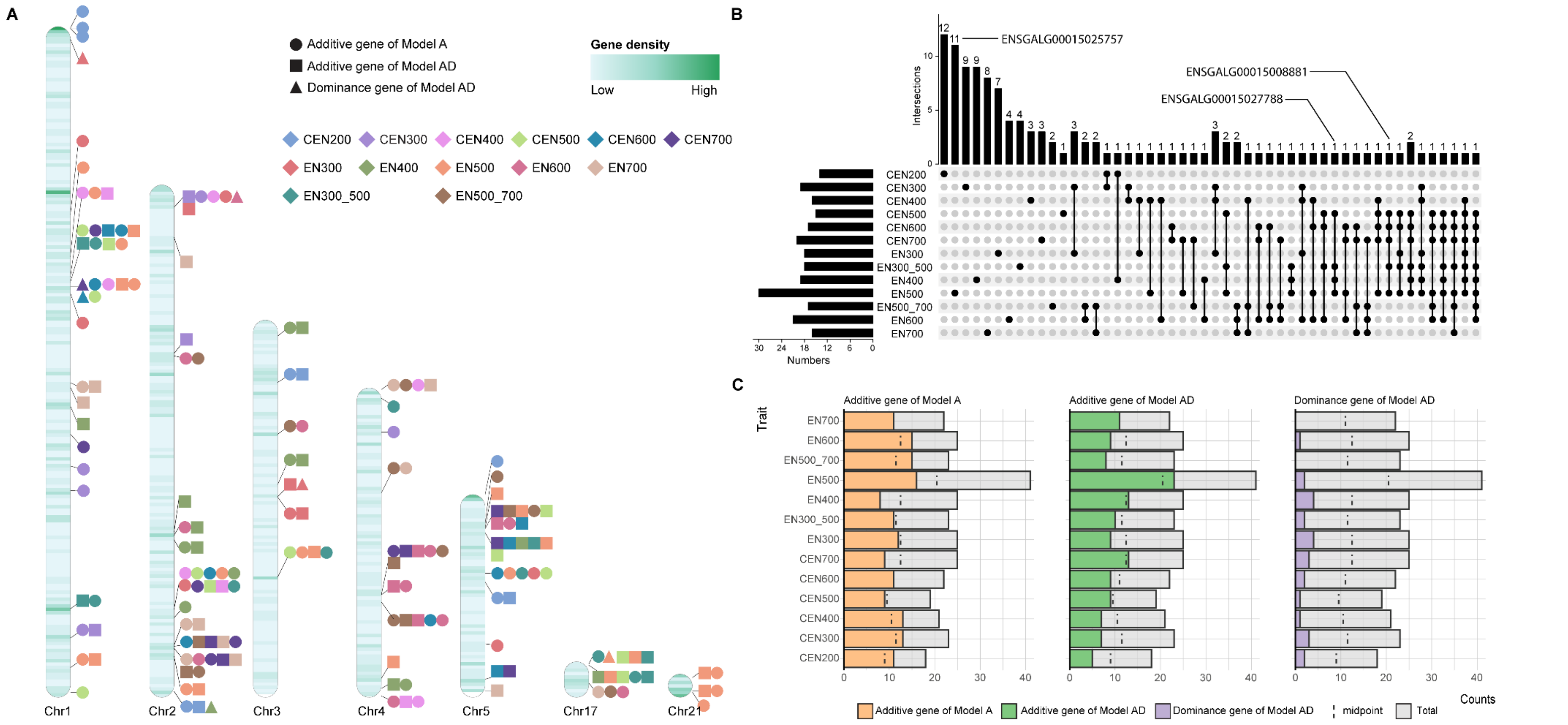

Fig. 5. Genes identified by TWAS analysis. **(A)** Statistically significant gene-trait associations identified by S-predixcan. Each association is arranged according to the SNP location on each chromosome and the points are color-coded by traits. Circle represented additive gene from additive model, box represented additive gene from additive-dominance model, and triangle represented dominance gene from additive-dominance model. Gene density was expressed as the averaged number of genes in a 1mb window. **(B)** Distribution of significant genes across traits. **(C)**

Distribution of significant genes across traits, filtered by models.

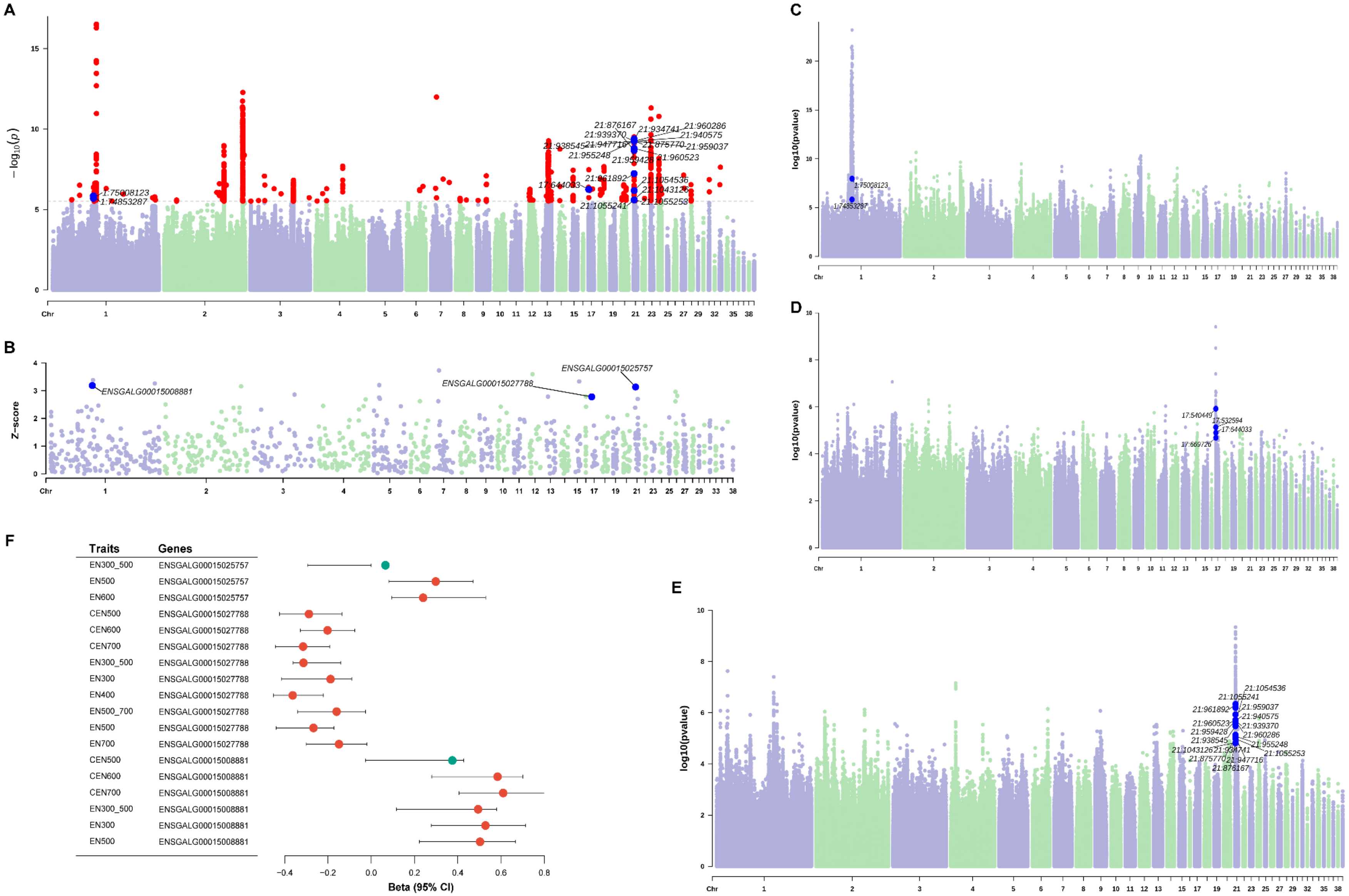
A
−log10(p)
1:75008123
1:74853287
21:876167
21:934741
21:960286
21:939370
21:940575
21:938545
21:947716
21:875770
21:959037
21:955248
21:959428
21:960523
17:644033
21:961892
21:1054536
21:1043126
21:1055241
21:1055253
Chr
B
Z-score
ENSGALG00015008881
ENSGALG00015027788
ENSGALG00015025757
C
log10(pvalue)
1:75008123
1:74853287
D
log10(pvalue)
17:540449
17:532594
17:644033
17:669726
E
log10(pvalue)
21:1054536
21:1055241
21:961892
21:959037
21:940575
21:960523
21:939370
21:959428
21:960286
21:938545
21:955248
21:1043126
21:934741
21:1055253
21:875770
21:947716
21:876167
F
Traits
Genes
EN300_500 ENSGALG00015025757
EN500 ENSGALG00015025757
EN600 ENSGALG00015025757
CEN500 ENSGALG00015027788
CEN600 ENSGALG00015027788
CEN700 ENSGALG00015027788
EN300_500 ENSGALG00015027788
EN300 ENSGALG00015027788
EN400 ENSGALG00015027788
EN500_700 ENSGALG00015027788
EN500 ENSGALG00015027788
EN700 ENSGALG00015027788
CEN500 ENSGALG00015008881
CEN600 ENSGALG00015008881
CEN700 ENSGALG00015008881
EN300_500 ENSGALG00015008881
EN300 ENSGALG00015008881
EN500 ENSGALG00015008881
Beta (95% CI)

Fig. 6. Multi-omics data analysis for egg production traits. **(A)** GWAS for trait EN500. Each dot represent one SNP, and associated SNPs were colored in red with threshold FDR 0.01. **(B)** TWAS for trait EN500. Each dot represent one gene. **(C)** eQTL mapping for gene *ENSGALG00015008881*. Each dot represent one SNP. **(D)** eQTL mapping for gene *ENSGALG00015025757*. Each dot represent one SNP. **(E)** eQTL mapping for gene *ENSGALG00015027788*. Each dot represent one SNP. **(F)** MR analysis for the three candidate genes. Green dots represent the insignificant associations, red dots represent the significant associations.

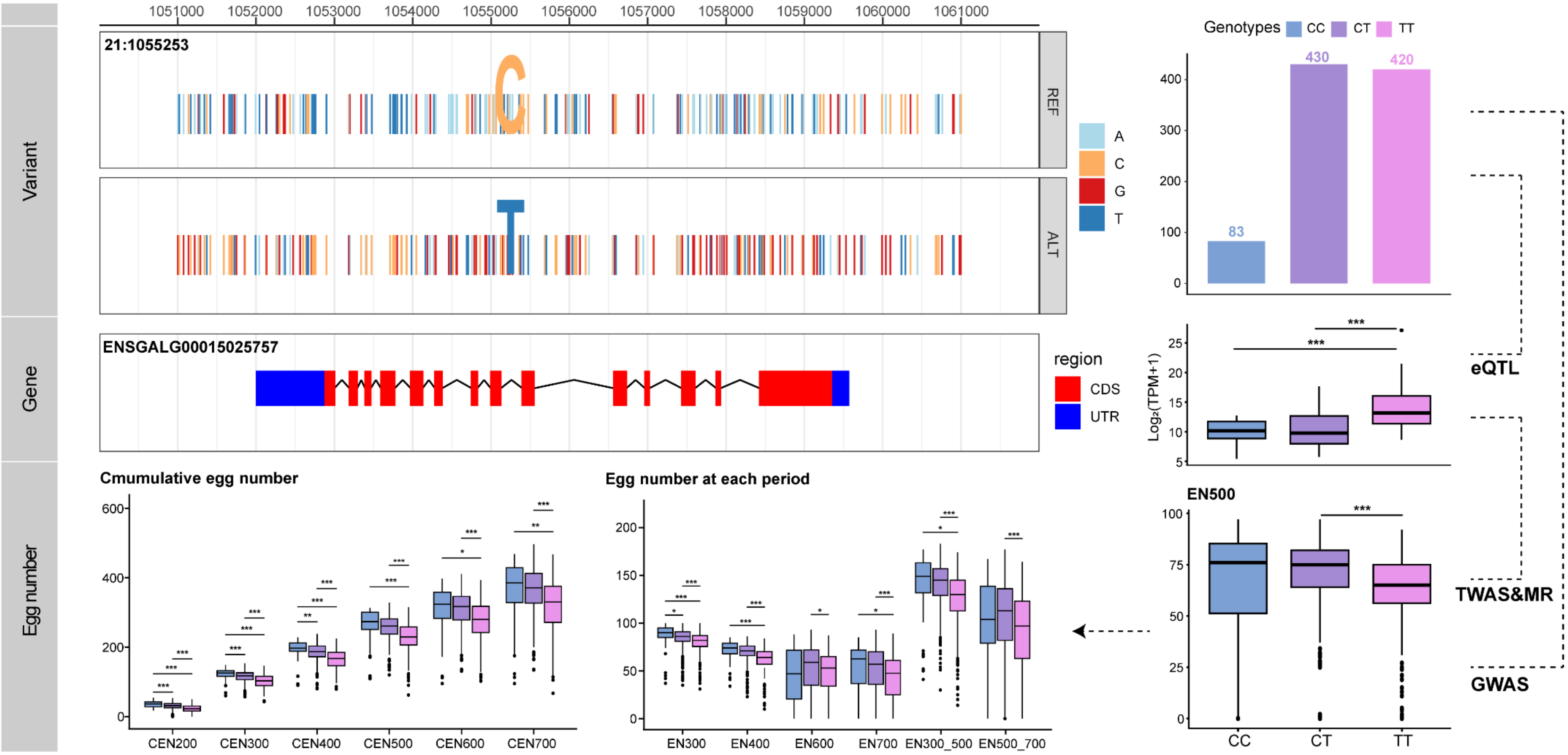

Fig. 7. The associations between candidate genetic variant 21:1055253 and gene expression (*ENSFALF00015025757*) and egg number.

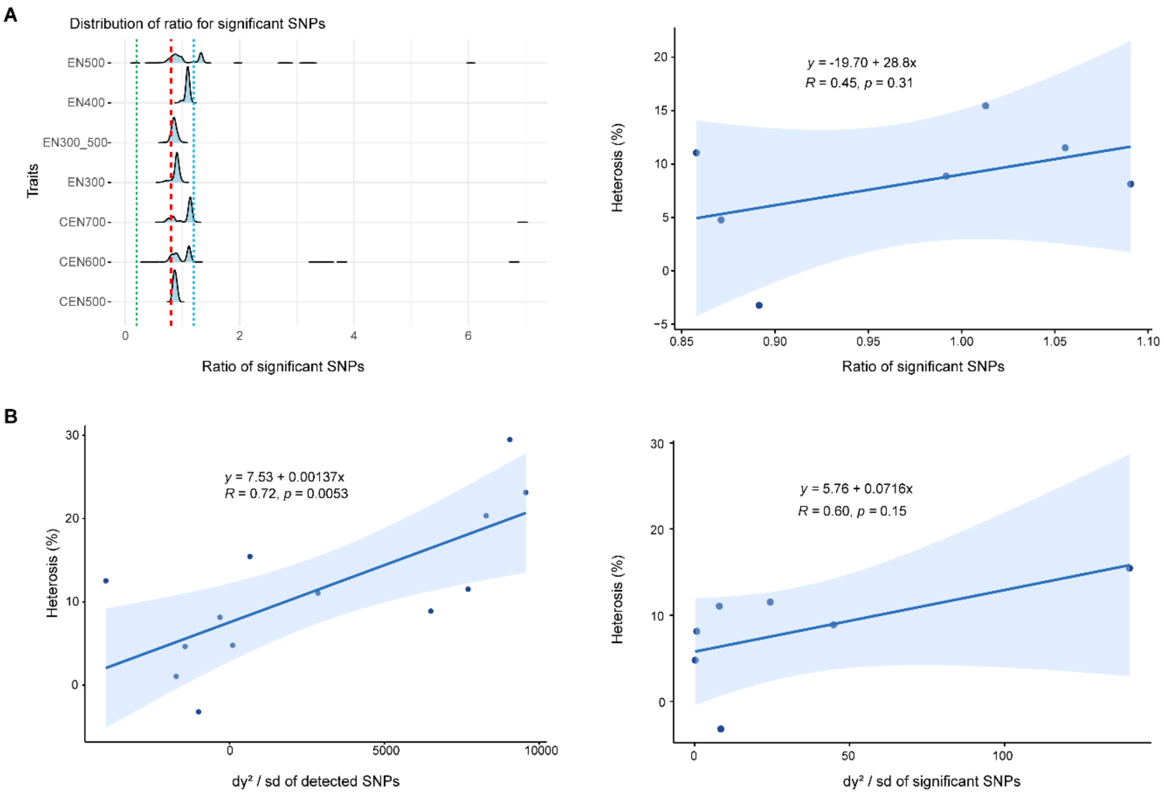

Fig. 8. Implications for heterosis of egg production traits. **(A)** Distribution of ratios for significant SNPs (green dash line: r = 0.2, red dash line: r = 0.8, blue dash line: r = 1.2) and correlation between ratio and heterosis. **(B)** Correlations between the sum of dy^2/sd of all SNPs (left panel) or only the significant SNPs (right panel) and heterosis.

Supplementary Material Figure

Click here to access/download
Supplementary Material
Additional_file_AN_20241010.docx

Supplementary Material Table

Click here to access/download
Supplementary Material
Additonal file Tables_AN_20241010.xlsx

Personal Cover Click here to access/download;Personal Cover;Cover letter.docx

Dear Editor,

We would like to submit a manuscript entitled "Identifying candidate genetic variants for egg number by analyzing over 1000 fully sequenced layers" by Aixin Ni, Henk Bovenhuis, Mario P.L. Calus, Yunlei Li, Jingwei Yuan, Yanyan Sun and Jilan Chen. We would like to have the manuscript considered for publication in *GigaScience* as a research article.

Egg production is one of the most important breeding goal traits for laying hens. We conducted a genome-wide association study using an additive-dominance model by analyzing 1,004 fully sequenced layers which were from a population of hybrid experiment. By integrating whole-genome sequencing and transcriptomic data, we conducted eQTL mapping and transcriptome-wide association studies, explored the genetic basis of egg number, and found three candidate genes. These results highlight the importance of dominance effects and the integration of multi-omics data in unraveling the genetic mechanisms governing egg production traits. The results here should be interesting for the animal geneticists, especially given the use of genomes from 1,004 fully sequenced layers.

We confirm that manuscript has not been published elsewhere, and it not under consideration by other journals. All authors have approved the manuscript and agree with its submission to *GigaScience.*

Thank you and with my best regards. We look forward to your response.

Sincerely yours,

Jilan Chen, PhD

Institute of Animal Science,

Chinese Academy of Agricultural Sciences

Beijing 100193, China

Tel: +86-10-62816005

E-mail: chen.jilan@163.com

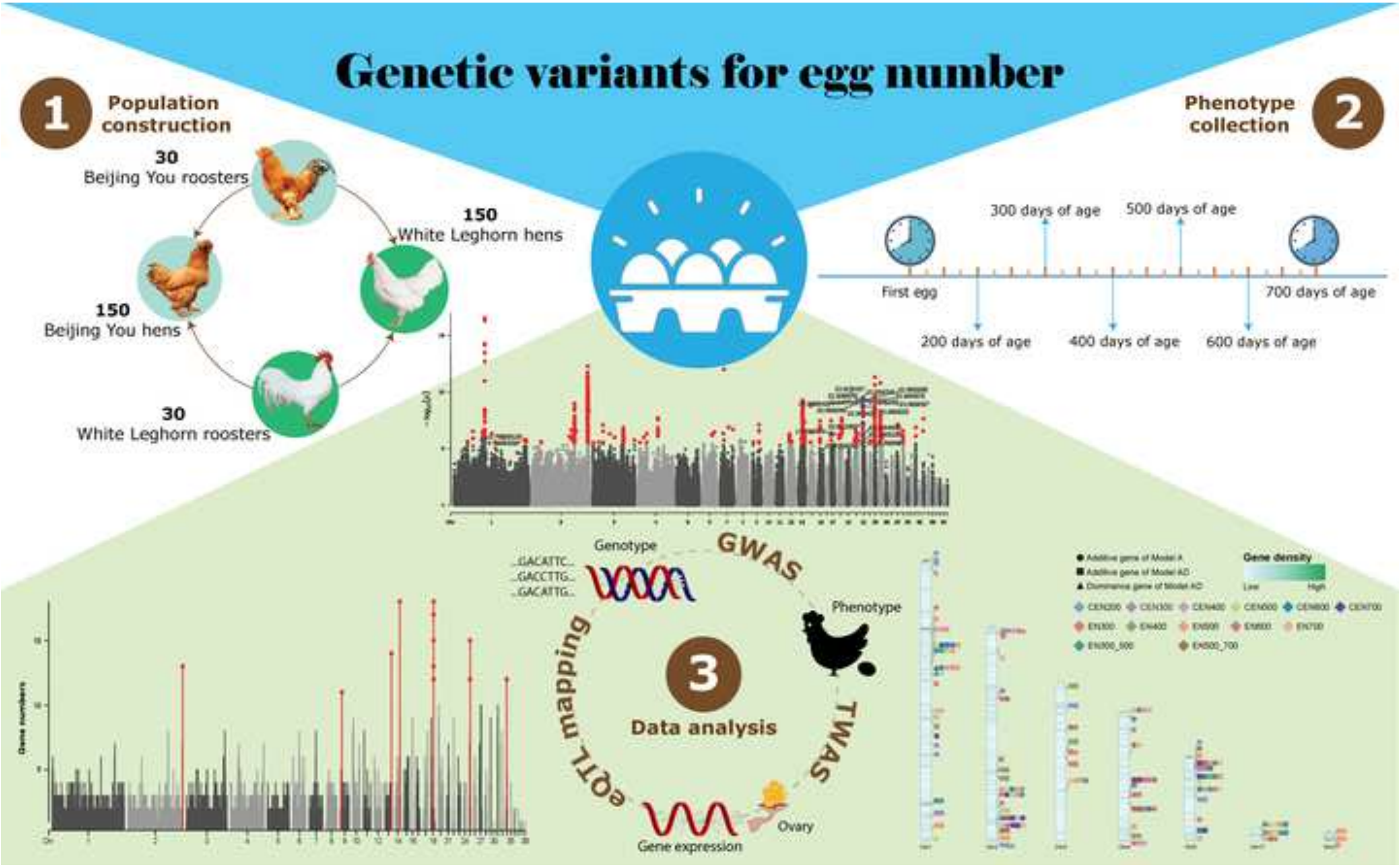
Genetic variants for egg number
1 Population construction
30 Beijing You roosters
150 White Leghorn hens
150 Beijing You hens
30 White Leghorn roosters
Phenotype collection 2
300 days of age
500 days of age
First egg
700 days of age
200 days of age
400 days of age
600 days of age
Genotype
GWAS
Phenotype
TWAS
Ovary
Gene expression
eQTL mapping
3
Data analysis
...GACATTC...
...GACCTTG...
...GACATTG...
Gene density
Low
High
