# Supplementary material for: Identifying candidate genetic variants for egg number by analyzing over 1,000 fully sequenced layers
Source: Gigascience. 2025 Jun 17;14:giaf064. doi: 10.1093/gigascience/giaf064 (PMC12203006; doi:10.1093/gigascience/giaf064)

GigaScience

Identifying candidate genetic variants for egg number by analyzing over 1000 fully sequenced layers

--Manuscript Draft--

**Manuscript Number:**	GIGA-D-24-00467R1
**Full Title:**	Identifying candidate genetic variants for egg number by analyzing over 1000 fully sequenced layers
**Article Type:**	Research
**Funding Information:**	National Natural Science Foundation of China (32172721) — Prof. Yanyan Sun<br>Agriculture Research System of China (CARS-40) — Prof. Jilan Chen<br>Agricultural Science and Technology Innovation Program (ASTIP-2021-IAS-16) — Prof. Jilan Chen
**Abstract:**	Background: Egg production over a long laying cycle until 700 days of age is fancied for modern layer chickens breeding. It is influenced by the onset of laying, stability during the peak period, and persistence at late laying stages. Conventional single-single nucleotide polymorphisms (SNP) association analyses have identified additive loci, but few studies have explored dominance effects or integrated multi-omics data to investigate the genetic basis of egg production traits from the onset to 700 days of age.A full diallel cross of 1,004 chickens was·subjected to whole-genome sequencing. Transcriptome data from the ovary was available for a subset of 120 chickens. A genome-wide association study (GWAS) was conducted using an additive-dominance model for cumulative egg number and egg number at different stages. Expression quantitative trait loci (eQTL) mapping was applied to investigate associations between SNPs and gene expression. A transcriptome-wide association study (TWAS) was conducted to explore the associations between gene expression and egg production traits to identify candidate genes.<br>Results: The additive-dominance model identified 5,892 significant SNPs, comprising 805 additive SNPs and 360 dominance SNPs shared between two or more traits. By integrating loci identified through GWAS with eQTL-mapping, the expression level of 27 genes were found to be associated with significant SNPs. Further integration with TWAS results revealed four novel candidate genes. For the loci with significant SNP effects, we found a positive but insignificant correlation between the ratios of dominance to additive effects and observed heterosis. Observed heterosis was positively correlated with heterosis predicted based on dominance effects and allele frequencies of all SNPs.<br>Conclusions: We identified candidate genetic variants for egg production traits by analyzing 1,004 fully sequenced layers. Detection benefited from incorporating dominance into the GWAS model. Traits with higher heterosis tended to be more affected by genes with dominant mode of action. Moreover, multi-omics data allowed to contribute to deciphering genetic mechanisms underlying egg production by establishing connections between genetic variants, gene expression, and egg number.<br>Key words: whole-genome sequencing, genetic variants, egg number, additive-dominance model, multi-omics, TWAS, heterosis
**Corresponding Author:**	Jilan Chen<br>Chinese Academy of Agricultural Sciences Institute of Animal Science<br>Beijing, CHINA
**Corresponding Author Secondary Information:**	
**Corresponding Author's Institution:**	Chinese Academy of Agricultural Sciences Institute of Animal Science
**Corresponding Author's Secondary Institution:**	
**First Author:**	Aixin Ni

Powered by Editorial Manager® and ProduXion Manager® from Aries Systems Corporation

**First Author Secondary Information:**	
**Order of Authors:**	Aixin Ni
	Henk Bovenhuis
	Mario P.L. Calus
	Yunlei Li
	Jingwei Yuan
	Yanyan Sun
	Jilan Chen
**Order of Authors Secondary Information:**	
**Response to Reviewers:**	Reviewer 1:<br>The study utilized genome and transcriptome data from a full diallel cross of 1,000 hens to conduct GWAS, eQTL mapping, and TWAS analyses. These analyses identified SNP loci and candidate genes that influence egg-laying traits. Incorporating dominant effects into the GWAS model was found to be beneficial. This research is significant for elucidating the molecular genetic mechanisms underlying egg-laying traits and for advancing genetic improvement efforts. However, there are several points in the Methods section that could be further improved:<br><br>1.Whole Genome Sequencing:<br>Why was the SNP filtering criterion for MAF set to less than 0.5%? This threshold seems too low.<br>Re: We thank the reviewer for the comments. To avoid the missing of rare, yet potentially important alleles, we considered the presence of up to 10 alternate alleles as valid, corresponding to a 0.5% MAF. This is now clarified in L163-165. Additionally, in the GWAS analysis, we applied further criterion: SNPs were eliminated if a genotype was observed in both WW and YY lines with five or fewer animals (L181-184).<br><br>2.Genome-Wide Association Study:<br>Typically, principal components are included in the GWAS model to correct for population stratification. Is the genetic group sufficient to correct for population stratification?<br>Re: Thanks for the comment. In our GWAS model, we included genetic group as fixed effect and used a relationship matrix to account for population stratification due to family relationships. The genetic groups coincide with the separation of individuals based on the first principal components. We added the PCA results in supplementary Fig. S8 and included relevant content in the main manuscript in L176-180.<br>What are the results of the GWAS using only the WY and YW populations?<br>Re: When performing GWAS on the WY and YW populations, we lose power due to decreasing the size of the data. We would like to keep the GWAS analysis with four genetic groups.<br>Additionally, the G matrix for the u vector is usually constructed using all SNPs except the tested SNP. How was the G matrix constructed in this study?<br>Re: The G matrix in our study was built for individuals with phenotypes for each trait and using all SNPs (apart from SNPs if a genotype was observed in both WW and YY lines with five or fewer animals for each trait). So we did not remove the tested SNP. We modified the relevant content in L181-184, L202-203 and L205-207.<br><br>3.Transcriptome Sequencing:<br>The data for the remaining 53 animals is not clearly described. Is this whole genome sequencing data?<br>Re: We have transcriptome sequencing data for ovaries of 120 animals, of which 67 also have whole-genome sequencing data. The remaining 53 animals were genotyped by mapping the transcriptome sequencing data to the reference genome, followed by imputation using the whole-genome sequencing data of the other 933 animas as the reference panel. We added more details about this process, including a comparison between the two data sources in L252-256, and L266-284. When doing this comparison, we further fine-tuned this process, by filtering SNPs from transcriptome sequencing (see L276-280).

Powered by Editorial Manager® and ProduXion Manager® from Aries Systems Corporation

4.eQTL Mapping:
Why were sequencing batch and PEERs not considered in the statistical model?
Re: Thanks for pointing out the PEER effects. We modified the eQTL mapping model by incorporating PEERs factors. Additionally, PEERs factors were also included in the construction of the TWAS expression prediction model. Please refer to L289-292, L295-297, and L303 for more details. We updated the results for eQTL-mapping and TWAS accordingly. Please refer to the updated Figs. 4-7 and the relevant content in L365-445, and L545-556.

5.Transcriptome-Wide Association Study and Mendelian Randomization Analysis:
Gene expression data was corrected for age in the study, and predicted expression levels were used in the association for egg numbers at different periods. Was it assumed that gene expression is constant throughout the entire egg-laying period in TWAS? Why were ovary samples collected for RNA sequencing at different ages? Can analyzing gene expression levels at different stages provide any useful information?
Re: The RNA samples were originally collected for a different experiment aimed at identifying differentially expressed genes at different stages.
In our TWAS analysis, we assumed that the same genotype would lead to consistent gene expression, thereby predicting the genetic contribution to gene expression. We also accounted for potential age-related variations in gene expression by correcting for age effects in our expression data.
Collect RNA samples at different ages would allow us to detect both age-dependent and age-independent genes associated with egg production traits. For instance, ENSGALG00015002757 influenced egg production throughout the entire laying period, ENSGALG00015027755 played a role primarily in early-stage egg production, ENSGALG00015009997 was associated with later-stage egg production. We added the relevant content in L388-396.

Reviewer 2:
Ni et al. sequenced 1004 chickens and delves into the genetic variants linked to egg production by integrating GWAS, TWAS, and eQTL analyses within a four diallel crossed populations. This study represents a significant contribution to our understanding of the genetic underpinnings of this economically vital trait. Nonetheless, the current manuscript has certain limitations that require attention to further enhance its scientific value.

Major comments:
1. Coherence of candidate SNPs, genes, and phenotypes: While the discussion on candidate genes is extensive, there is a notable lack of coherence in linking these candidate SNPs, genes, and phenotypes. Therefore, this section should be reorganized and expanded to enhance clarity and scientific rigor.
Re: Thanks for the comment. We updated the results following the inclusion of PEER factors in the eQTL mapping analysis and worked to improve the coherence, with relevant content added in L545-556. However, due to the lack of other explanatory information such as the annotation for regulatory elements, we are unable to confirm whether the SNPs are located within regulatory regions or have specific functions that impact gene expression (as now mentioned in the added part in the Discussion).

2. cis-eQTL additivity: This paper, with the DOI number of 10.1186/s13059-023-03060-2, suggests that the majority of cis-eQTLs exhibit additive effects. It is highly recommended to conduct further analysis to verify whether a comparable pattern can be observed in the current study, and to augment the discussion section with a more comprehensive analysis of the obtained results.
Re: With our dataset, we conducted the analysis, and observed an even distribution of cis- and trans-eQTLs across the genome for all types of gene action. We added relevant content and potential explanations in the discussion part in L529-542.

3. One key conclusion is that incorporating dominance effects into the GWAS model enhances detection. The authors should discuss whether this approach could be generalized to other traits or even to other species.

Powered by Editorial Manager® and ProduXion Manager® from Aries Systems Corporation

Re: Thanks for the comments. We now elaborate more on this in L494-501. We compared the results incorporating dominance into GWAS model, and found inconsistent results. We cannot generalized this to other traits or species.

4. Exclusion of sex chromosome variants: The exclusion of sex chromosome variants from the dataset should be justified.
Re: Since we only have one allele for sex chromosomes (in chickens the female is the heterogametic sex), and, the genotype matrix (and thus the effect) of additive and dominance SNP effects cannot be distinguished in Model AD. We decided to exclude the sex chromosome from our analyses. We added relevant content in the Material and Method part in L221-224.

Minor Comments:
1.Please add the line number in your next manuscript version.
Re: Done as requested.

2.MM-Transcriptome sequencing section. In the 120 samples, genomic data of 67 samples were directly obtained from whole genome sequencing, while the genomic data of the other 53 samples were derived from transcriptome sequencing. Please address the correlation and potential bias between these two methods.
Re: Thanks for the suggestions. We added the relevant content regarding the difference in genotype calling between whole-genome sequencing data and transcriptome data. After comparison, we remove SNPs with high percentage of missing genotypes, which resulted in an increase in concordance. Please refer to L252-256, and L266-284 for more details.

3.Result-Genome-wide association study and candidate variants section. "The number of significant additive and dominance SNPs detected by Model AD was 3,294 and 2,598, while no significant SNPs were detected by Model A". Please explain why so many additive SNPs were detected in the AD model but not a single one was detected in the A model? Shouldn't the A model be much more specific in detecting additive SNPs? Which result should we believe?
Re: In the current study, Fig. 3A shows the highly consistent additive SNP effects across the two models, indicating that additive SNP effects in Model A remained largely unchanged in Model AD. This suggests that both models have similar power to detect additive SNP effects, while the significant SNPs exhibited more extreme effects in Model AD. We considered the possibility that opposing additive and dominance SNP effects in Model A can potential cancel out, as pointed out in Fig. 3B. Our findings suggest that Model AD may be more suitable to detect trait-related variants in a context specific manner. We added some explanations in L351-353 and L482-501 in the revised manuscript.

4.Fig 2D, It appears that the two sections of fig2D complement each other and can be combined into one, forming an "additive + dominance" plot. Please replot the fig2D or explain what is the difference between these two sections and what the "total" mean is?
Re: Thanks for the suggestions. We combined the two sections into one.

5."Among the coding consequences, the most abundant variants are synonymous variants with the similar proportion ~0.70 in significant and all SNPs, which were non-neutral (47)." Please explain the "non-neutral" in here.
Re: Done as requested. Although synonymous variants are typically considered neutral, some studies suggest that these variants may have consequences on the transcriptome process. We added explanations in L341-345.

6."The additive and dominance SNP effects from Model AD are strongly related to each other (Fig. 3A). These patterns were similar for other chromosomes and traits. For all significant SNPs, most of the additive SNP effects are negative, while most of the dominance effects are positive(Fig. 3C)." Please explain why most effects of additive and dominance are oppsite but strongly related to each other.
Re: Thanks for the suggestions. We expected a higher frequency of homozygotes for the reference allele in WW since we used the reference genome of WW. Additionally, WW is a breed known for high egg production, and we coded the genotype based on

Powered by Editorial Manager® and ProduXion Manager® from Aries Systems Corporation

	the alternative allele, these may have resulted in negative additive SNP effects. Additionally, given that the two pure lines are genetically distinct and considering the substantial heterosis observed in the crossbreds in our previous study, the dominance SNP effects for many SNPs were positive. This explains the opposite sign for the two SNP effects. We added some discussion on this in L511-518 in the revised manuscript. Furthermore, the significant SNPs we detected are beneficial in both homozygous and heterozygous forms, this could explain why we have a strong relationship between additive and dominance SNP effects, which we now added as additional figure Fig. S22 and we have rephrased the content in the manuscript in L518-528.<br><br>7.Table 3. The performance of egg production in the two crossed populations resembles that of their paternal lines, with YW resembling YY and WY resembling WW. This phenomenon appears to be more indicative of imprinting rather than typical heterosis. Please explain why the consideration of paternal imprinting was overlooked.<br>Re: We added relevant content about the difference of reciprocal crosses, and mentioned some possible reasons that explain this observed difference, including paternal imprinting. Please check L589-595 for more details.<br><br>Lines 31-32: Please specify the number of animals with transcriptome data.<br>Re: Done as requested. Please see L31 in the revised manuscript.<br><br>Lines 33-34: Expand the content related to eQTL mapping, as it is an important component of the study.<br>Re: Done as requested. Please see L33-35 in the revised manuscript.<br><br>Lines 37-38: Are "805" and "360" unique counts?<br>Re: Yes, these represent the unique counts. We changed the word “with” to “comprising” to clarify that they are unique counts. Please see L38-39 in the revised manuscript.<br><br>Lines 289-293, 295-297: The dominance effects contribute substantially to the total variance for egg number at different stages but not the cumulative egg number. Please elaborate on this discrepancy in the discussion section.<br>Re: Thanks for the comment. We agree with your point, however, we did not have sufficient information to fully support our interpretation. One possible explanation is that dominance effects may be age-dependent, with their direction varying over time, which could result in the absence of variance for cumulative egg number at certain stages. Given this limitation, we did not expand on this discussion further in the manuscript.<br><br>Line 363: The Manhattan plots for other traits would provide a more comprehensive view.<br>Re: We added the Manhattan plots for other traits as additional figures in Figs. S19-S21.<br><br>Fig. 6: The candidate variants do not appear to be the lead variants in GWAS or eQTL analyses. Please address this.<br>Re: For the leading variants, no significant expressed genes were identified as being related, suggesting that they may not be expression quantitative trait loci but could influence egg number through other mechanisms. We added some discussion on this in L430-433 in the revised manuscript.<br><br>Table 2: Include gene symbols when it is available. Additionally, ensure consistent font sizes across the table, particularly in the last two columns.<br>Re: Done as requested.
**Additional Information:**	
**Question**	**Response**
Are you submitting this manuscript to a special series or article collection?	No
**Experimental design and statistics**	Yes

Full details of the experimental design and statistical methods used should be given in the Methods section, as detailed in our Minimum Standards Reporting Checklist. Information essential to interpreting the data presented should be made available in the figure legends.<br><br>Have you included all the information requested in your manuscript?	
**Resources**<br><br>A description of all resources used, including antibodies, cell lines, animals and software tools, with enough information to allow them to be uniquely identified, should be included in the Methods section. Authors are strongly encouraged to cite Research Resource Identifiers (RRIDs) for antibodies, model organisms and tools, where possible.<br><br>Have you included the information requested as detailed in our Minimum Standards Reporting Checklist?	Yes
**Availability of data and materials**<br><br>All datasets and code on which the conclusions of the paper rely must be either included in your submission or deposited in publicly available repositories (where available and ethically appropriate), referencing such data using a unique identifier in the references and in the "Availability of Data and Materials" section of your manuscript.<br><br>Have you have met the above requirement as detailed in our Minimum Standards Reporting Checklist?	Yes
GigaScience has policies and guidelines in place for the use of generative AI-writing tools such as ChatGPT. If you have used such writing tools to assist with	No

writing the manuscript this must be declared and cited in the text. Authors should not list AI-writing tools and other AI-assisted technologies as an author or co-author and should acknowledge that they are fully responsible for text generated or refined by AI-writing tools.<p><br><br>A summary of use (particularly in the introduction or among methods) needs to be included at the end of the paper, and the outputs should also be included as a supplementary file hosted in GigaDB or other open repositories. Please read our guidelines for more information. <p><br><br>By submitting to GigaScience, you are aware of the journal's AI-writing tools policy, and if you have declared use of such tools below, you have acknowledged this where appropriate in your manuscript and have made a summary of use and outputs available. <p><br><b>AI-assisted writing tools have been used in the preparation of this manuscript?	

Manuscript
Click here to access/download;Manuscript;R1_EP_AN_20250515_clean_1.d

Identifying candidate genetic variants for egg number by analyzing over 1000 fully sequenced layers

Aixin Ni[1,2], Henk Bovenhuis[2], Mario P.L. Calus[2], Yunlei Li[1], Jingwei Yuan[1], Yanyan Sun[1,*], Jilan Chen[1,*]

[1]State Key Laboratory of Animal Biotech Breeding, Key Laboratory of Animal (Poultry) Genetics Breeding and Reproduction of Ministry of Agriculture and Rural Affairs, Institute of Animal Science, Chinese Academy of Agricultural Sciences, 100193 Beijing, China

[2]Animal Breeding and Genomics, Wageningen University and Research, P.O. Box 338, 6700 AH Wageningen, the Netherlands

*Correspondence address: Yanyan Sun, Institute of Animal Science, Chinese Academy of Agricultural Sciences, 100193 Beijing, China. E-mail: sunyanyan02@caas.cn; Jilan Chen, Institute of Animal Science, Chinese Academy of Agricultural Sciences, 100193 Beijing, China. E-mail: chen.jilan@163.com

E-mail addresses:

Aixin Ni: aixin.ni@wur.nl

Henk Bovenhuis: henk.bovenhuis@wur.nl

Mario P.L. Calus: mario.calus@wur.nl

Yunlei Li: liyunlei@caas.cn

Jingwei Yuan: yuanjingwei@caas.cn

Yanyan Sun: sunyanyan02@caas.cn

Jilan Chen: chen.jilan@163.com

ORCID iDs: Aixin Ni [0009-0002-7938-1231]; Henk Bovenhuis [0000-0002-9074-5334]; Mario P L Calus [0000-0002-3213-704X]; Yunlei Li [0000-0001-8117-6232]; Jingwei Yuan [0000-0001-7993-0742]; Yanyan Sun [0000-0001-6425-7786]; Jilan Chen [0000-0002-9400-009X]

Abstract

Background: Egg production over a long laying cycle until 700 days of age is fancied for modern layer chickens breeding. It is influenced by the onset of laying, stability during the peak period, and persistence at late laying stages. Conventional single-single nucleotide polymorphisms (SNP) association analyses have identified additive loci, but few studies have explored dominance effects or integrated multi-omics data to investigate the genetic basis of egg production traits from the onset to 700 days of age. A full diallel cross of 1,004 chickens was·subjected to whole-genome sequencing. Transcriptome data from the ovary was available for a subset of 120 chickens. A genome-wide association study (GWAS) was conducted using an additive-dominance model for cumulative egg number and egg number at different stages. Expression quantitative trait loci (eQTL) mapping was applied to investigate associations between SNPs and gene expression. A transcriptome-wide association study (TWAS) was conducted to explore the associations between gene expression and egg production traits to identify candidate genes.

Results: The additive-dominance model identified 5,892 significant SNPs, comprising 805 additive SNPs and 360 dominance SNPs shared between two or more traits. By

integrating loci identified through GWAS with eQTL-mapping, the expression level of 27 genes were found to be associated with significant SNPs. Further integration with TWAS results revealed four novel candidate genes. For the loci with significant SNP effects, we found a positive but insignificant correlation between the ratios of dominance to additive effects and observed heterosis. Observed heterosis was positively correlated with heterosis predicted based on dominance effects and allele frequencies of all SNPs.

Conclusions: We identified candidate genetic variants for egg production traits by analyzing 1,004 fully sequenced layers. Detection benefited from incorporating dominance into the GWAS model. Traits with higher heterosis tended to be more affected by genes with dominant mode of action. Moreover, multi-omics data allowed to contribute to deciphering genetic mechanisms underlying egg production by establishing connections between genetic variants, gene expression, and egg number.

Key words: whole-genome sequencing, genetic variants, egg number, additive-dominance model, multi-omics, TWAS, heterosis

Background

The global egg production (in tons) has been doubled since 1990 and eggs play crucial roles in providing high-quality and low-cost animal protein for the growing population [1]. Egg production is not only a reflection of laying efficiency and economic efficiency but also one of the most important breeding goal traits for laying hens. Combining genomic information and knowledge of the genetic architecture of traits are of increasing importance in selective breeding. Nowadays, to reduce brooding costs and to lower environmental impact, there is a trend to extend the laying cycle from the traditional 500 to 700 days of age [2]. This emphasizes the need for studies to look for

loci underlying egg production traits throughout the laying period, especially during the extended period, to provide insights for selective breeding.

Quantitative trait loci (QTL) mapping and genome-wide association study (GWAS) have revealed genomic variants statistically associated with egg production traits. About 115 QTL on 27 chromosomes were reported to be associated with egg number in chickens [3]. While GWAS establishes the connection between genotype and phenotype, the underlying biological mechanisms remain unclear. Furthermore, most of the identified variants are located in non-coding regions and several variants are in high linkage disequilibrium (LD), making it difficult to determine the causal variants. These issues can be alleviated by using multi-omics strategies such as expression quantitative trait loci (eQTL) mapping, transcriptome-wide association study (TWAS) and Mendelian randomization (MR) analysis to unravel the underlying genetic architecture of complex traits. eQTL-mapping identifies genomic regions associated with the expression levels of genes based on single nucleotide polymorphisms (SNP) genotypes and gene expression data. TWAS establishes a connection between gene expression and phenotypes by predicting gene expression levels in genotyped animals. It then leverages the summary-level GWAS results and expression data to identify the expression of gene and phenotypes associations. MR provides evidence for putative causal relations between gene expression and phenotypes [4]. The joint analysis of genomic and transcriptomic data has contributed to deciphering the biological functions of candidate genes for various traits in cattle [5], pigs [6, 7], and chickens [8].

Another limitation of many GWAS studies is that non-additive effects are often ignored. Dominance is believed to be common in mammals [9] and has been studied in the context of genetic parameter estimation, genomic selection, and genomic prediction in several farm animals for traits such as carcass weight of cattle [10], body weight of

quails [11], reproductive performance of dairy cattle [12], and growth of tilapia [13]. In chickens, Amuzu-Aweh found that dominance variance accounted for up to 37% of the genetic variance, and up to 6% of the phenotypic variance in egg number depending upon the line [14], highlighting the substantial role of non-additive genetic effects. Furthermore, loci with dominance effects have been identified in several species for different traits, contributing to phenotypic variance. In cattle, one key genetic variant of sperm motility demonstrated a much higher significance of the non-additive effects compared to additive effects (P = 1.0E-31 for dominance and P = 1.1E-08 for additive) [15]. Dominance loci could explain 12% to 13% of phenotypic variance in sheep's resistance to *Haemonchus. contortus* and 0.69% to 0.84% of phenotypic variance in broilers' egg number [16, 17]. These examples underscore the importance and potential of considering dominance effects to gain a more comprehensive understanding of the genetic architecture underlying complex traits.

Examining the role of SNPs, particularly non-additive effects, might provide valuable insights into heterosis, which is thought to be driven by non-additive genetic effects [18]. Quantitative genetic theory suggests that heterosis, expressed as the difference between the crossbreds and the mid-parent value, is proportional to the sum of the dominance effects multiplied by the squared difference in allele frequency between the parental lines [18]. Moreover, the integration of genomic and transcriptomic data has led to the identification of heterosis-related genetic variants in several plants. For instance, *RH8* was identified as a heterosis-related candidate gene for yield in rice [19], structural variants in *ZAR1* and *ZmACO2* were found to increase heterosis for yield in maize [20], and a CACTA-like transposable element upstream of *BnaA9.CYP78A9* was shown to contribute to the heterosis of cell number in oilseed rape [21]. In the current study, we aimed to identify additive and non-additive candidate genetic variants for egg

number, and explore the genetics underlying these complex traits using multi-omics data, and discuss implications for heterosis. To achieve our objective, we sequenced the genome of 1,004 animals, which enabled including all genome-wide segregating variants in the analysis, and would increase the power to detect variants associated with egg production. We employed a model incorporating both additive and dominance SNP effects and combined this with transcriptome data of ovary tissue to map eQTLs. These analyses were followed by a TWAS to prioritize candidate genes for egg production traits. The flowchart of analyses to identify candidate genetic variants for egg production traits is shown in Fig. 1. Estimated dominance SNP effects were used to investigate its relationship with observed heterosis.

Methods

Resource population and phenotypic data

Animal resources used in our study were previously described in detail [22]. Briefly, four genetic groups were used, pure line Beijing-You chickens (YY) and White Leghorns (WW); and their reciprocal crosses with either Beijing-You (YW) or White Leghorns (WY) as the sire line. The YW and YY animals were created using the same 30 Beijing-You sires, and the WY and WW were created using the same 30 White Leghorns sires. The chickens were kept in individual cages in the same hen house during the experiment. Cumulative egg number and egg number at different stages from 200, till 700 days of age were recorded, CEN200 (cumulative egg number till 200 days of age), CEN300, CEN400, CEN500, CEN600, CEN700, EN300 (egg number from 200 to 300 days of age), EN400, EN500, EN600, EN700, EN300_500, EN500_700, were computed from individual egg-laying recordings. In the current study, we defined the time period before 300 days of age as the early stage, 300 to 500 days of age as the

middle stage, and 500 to 700 days of age as the late stage. Observed heterosis was estimated for different traits based on the predicted mean phenotypes for each genetic group, using the "predict" statement in ASReml 4.2 following the model described previously [22].

Whole genome sequencing

Genomic DNA was extracted using the phenol-chloroform method. The genomes of 1,004 chickens were sequenced at ~15.98× coverage (Supplementary Fig. S1), containing 210 WW, 240 WY, 268 YY, and 286 YW. After sequencing, FastQC (RRID:SCR_014583) was used to evaluate the quality of sequencing [23].

Trimmomatic (RRID:SCR_011848) was used to remove adapters and the low quality reads with the following parameters: LEADING:3 TRAILING:3 SLIDINGWINDOW:4:15 and MINLEN:15 [24].

Clean reads were aligned using BWA-MEM (v0.7.17, RRID:SCR_022192) [25]. Samblaster (v0.1.26, RRID:SCR_000468) was used to mark duplicates [26], and Samtools (v1.14, RRID:SCR_002105) to sort and index the BAM files [27]. Freebayes (v1.3.1, RRID:SCR_010761) was used with the chicken reference genome (GRCg7W) [28] for variant calling with: --use-best-n-alleles 4 --min-base-quality 10 --min-alternate-fraction 0.2 --haplotypelength 0 --ploidy 2 --min-alternate-count 2 [29]. The vcffilter module from vcflib (v0.00.2019.07.10, RRID:SCR_001231) was used to discard variants with low phred quality score (<=20) [30]. Tabix, a module from htslib (v1.9), was used to index the VCF files [30]. Alignment quality control statistics were computed with QualiMap (v.2.2.2-dev, RRID:SCR_001209) [31].

A total of 16,828,475 variants were called. After removing indels with Plink (v1.9, RRID:SCR_001757) [32], 14,119,765 SNPs were retained. The data were further

filtered with Plink using the following criteria: genotyping call-rate for SNPs < 95% and for individuals < 95%, minor allele frequency < 0.5%, and only for pure lines individuals a test for Hardy Weinberg equilibrium $P < 1.0E-04$. The cut-off of 0.5% for minor allele frequency corresponds to a requirement that for each locus the minor allele should be observed at least ten times in the data. After those filters, 12,495,895 SNPs and 986 animals were retained.

Due to missing genotypes from the parents, it is not possible to compare the parent-offspring relationship. Instead, we compared pedigree and genomic relationships among the 986 animals. We removed conflicting animals manually using the following steps, which were performed within WW, YY, and combined crossbred genetic groups (WY & YW): calculated pedigree and genomic relationships, and sorted the animals within the genetic groups based on descending absolute difference between pedigree and genomic relationships. In the top 100 of those relationships, we counted the occurrences for each animal. Starting from the top, we removed from every of those 100 relationships the animal that was involved in the largest number of "conflicts". This process improved the credibility of the genomic data (Supplementary Figs. S2-S7). The principal component analysis (PCA) revealed that the grouping based on the first two principal components, that accounted for 63.47% (PC1) and 5.59% (PC2) of the total variance for the animals retained after quality control, coincides with the four genetic groups (Supplementary Fig. S8). After quality control, 933 animals were retained for subsequent analysis. Beagle (v.4.1, RRID:SCR_001789) was used to impute missing genotypes [33]. To avoid confounding between genetic group and genotype in the statistical analysis, SNPs were eliminated if one genotype class was observed in both WW and YY lines with five or fewer animals.

Variance components estimation

Additive and dominance variances were estimated with restricted maximum likelihood method. For each trait, two models were fitted in Wombat [34]: one with only additive effects (Model A), the other with additive and dominance effects (Model AD). Model A was:

$$\mathbf{y} = \mathbf{X_1 b_1} + \mathbf{u} + \mathbf{e}$$

where $\mathbf{y}$ was the vector with phenotypic values, $\mathbf{b_1}$ was a vector of the fixed effects including genetic group (WW, YY, YW and WY) and rack effects, $\mathbf{X_1}$ was the corresponding design matrix, $\mathbf{u}$ was the vector of the random animal effects with $N(\mathbf{0}, \mathbf{G}\sigma_a^2)$, where $\mathbf{G}$ was the genomic relationship matrix and σ_a^2 was the additive genetic variance, $\mathbf{e}$ was the vector of random residual effects with $N(\mathbf{0}, \mathbf{I}\sigma_e^2)$, where $\mathbf{I}$ was the identity matrix and σ_e^2 was the residual variance.

Model A was extended with a dominance deviation as Model AD:

$$\mathbf{y} = \mathbf{X_1 b_1} + \mathbf{u} + \mathbf{v} + \mathbf{e}$$

where $\mathbf{v}$ is a vector of random dominance deviations with $N(\mathbf{0}, \mathbf{D}\sigma_d^2)$, where $\mathbf{D}$ was the dominance relationship matrix and σ_d^2 was the dominance variance.

The genomic relationship matrix was computed with program Calc_grm according to the first version of VanRaden [35], $\mathbf{G} = \frac{\mathbf{ZZ'}}{2\sum_{p_i} p_i(1-p_i)'}$, $\mathbf{Z}$ is the matrix of SNP genotypes (coded as 0, 1, 2) for individuals with phenotypes for each trait, and p_i is the frequency of the counted allele at SNP i. The dominance relationship matrix was computed with program Calc_grm according to Vitezica et al [36], $\mathbf{D} = \frac{\mathbf{MM'}}{\sum_i (2p_i(1-p_i))^2}$, where $\mathbf{M}$ is a matrix of heterozygote coefficients (codes as 0, 1, 0) for individuals with phenotypes for each trait. When individual j is homozygous for locus i, $M_{ij} = 0 - 2p_i(1-p_i)$,

and when it is heterozygous, $M_{ij} = 1 - 2p_i(1 - p_i)$.

Genome-wide association study

A single SNP GWAS was performed to estimate additive and dominance effects per SNP. For each SNP, the following model was fitted for Model A:

$$\mathbf{y} = \mathbf{X_1 b_1} + \mathbf{j}\alpha + \mathbf{u} + \mathbf{e}$$

where $\mathbf{j}$ was a vector with allele counts (coded as 0, 1, and 2 when homozygote for the reference allele, heterozygote, and homozygote for the alternative allele, respectively); α is the additive effect.

Model AD was extended with an additive and a dominance SNP effects:

$$\mathbf{y} = \mathbf{X_1 b_1} + \mathbf{j}\alpha + \mathbf{k}\beta + \mathbf{u} + \mathbf{v} + \mathbf{e}$$

where $\mathbf{k}$ was a vector with heterozygosity status (coded as 0, 1, and 0 when homozygote for the reference allele, heterozygote, and homozygote for the alternative allele, respectively); β is the dominance effect. The genomic relationship matrix was computed using the same method as described above. SNPs on the sex chromosomes were excluded from the dataset, as Model AD does not allow for differentiation between additive and dominance SNP effects in the genotype matrix when only one allele is observed at a locus, which is the case for the ZW females.

Solutions and t-statistics of the SNP effects were obtained from the output of Wombat, and corresponding *P*-values were computed. The genome-wide significance threshold for the SNP effects was based on a false discovery rate (FDR). FDR was calculated using the R-package "qvalue" (RRID:SCR_001073) and FDR < 0.01 was considered significant. Manhattan and Q-Q plots were derived from the GWAS results using the R-package "CMplot" (RRID:SCR_024514) [37]. The variant effect predictor (VEP,

RRID:SCR_007931) software [38] was used to predict the maximal consequence of the significant SNPs. For comparison, we also calculated the maximal consequences of all SNPs. The SNP ratio was calculated based on the T-statistics from the Model AD, $r = \left|\frac{t_{Dom}}{t_{Add}}\right|$ [9]. T-statistics (t_{Dom} and t_{Add}) is the ratio of the estimated SNP effect and its standard error. Based on the SNP ratios, SNPs were considered additive ($r < 0.2$), partial-dominance ($0.2 < r < 0.8$), complete-dominance ($0.8 < r < 1.2$), or over-dominance ($r > 1.2$) [9]. In addition to the SNP ratios, we calculated the sum of the dominance effects (d) multiplied by the squared difference in allele frequency (y) between the parental lines (dy^2) for all SNPs and significant SNPs to investigate the relation with observed heterosis. To enable comparison across different traits, we standardized the dominance SNP effects based on the phenotypic standard deviation of the trait.

Transcriptome sequencing

From each of the four genetic groups, six chickens were randomly selected at 150, 250, 320, 500, and 700 days of age to collect ovaries for RNA sequencing, yielding 120 samples in total. Total RNA was isolated from the tissue of each hen using TRIzol® Reagent (Invitrogen, Carlsbad, CA, USA) according to the manufacturer’s guidelines. RNA-seq was performed using Novaseq 6000 (Illumina, San Diego, USA) to generate 150 bp paired-end reads. Quality control, mapping, and transcriptome assembly were done following the steps described previously [39], getting the transcripts per kilobase per million mapped reads (TPM) for each gene when mapped to the chicken reference genome (GRCg7W).

Of the 120 samples with transcriptome sequencing data, genotypic data for 67 of the samples was obtained from the whole genome sequencing. Genotypic data for the

remaining 53 animals was obtained based on transcriptome sequencing data and missing genotypes were imputed using the whole genome sequencing data as reference panel. For RNA sequencing, STAR (v.2.7.11a, RRID:SCR_004463) was used to map the high-quality reads to the chicken reference genome (GRCg7W) with average mapping rate 93.76% (Supplementary Fig. S9) [40]. Picard (v. 2.7.1, RRID:SCR_006525) was used to sort the BAM files, mark duplicates and reorder BAM files [41]. Samtools (v. 1.14) was used to index the BAM files [27]. GATK (v. 4.2.6.1, RRID:SCR_001876) was used to split the overlapping intron reads and detect variants [42]. The CombineGVCFs function was then used to jointly genotype all these samples into one GVCF per tissue. "GenotypeGVCFs" was used to transfer GVCF to VCF file, and SNPs were extracted using SelectVariants and filtered with "QD < 2.0 || MQ < 40.0 || FS > 60.0 || SOR > 3.0 || MQRankSum < -12.5 || ReadPosRankSum < -8.0". Beagle (v. 4.1) was used to impute the missing genotype using the whole genome sequencing data as the reference panel [33]. To evaluate the imputation accuracy, we used the 67 animals which had genotypes based on transcriptome sequencing data and whole genome sequencing. Using genotypes from the transcriptome sequencing, we imputed missing genotypes, either using the whole-genome sequence data across the three genetic groups (WW, YY and YW/WY), or using only data from the genetic group to which the animal belonged. We calculated the concordance using the "pdiff" function in Plink, and found a very similar distribution of concordance for SNPs and individuals in both cases (imputation based on all groups or based on their own genetic group - Supplementary Fig. S10). Based on these results, we decided to do the imputation for the 53 animals using whole-genome sequence data of all three genetic groups. Subsequently, when using the 67 animals, we evaluated the impact of removing SNPs with low quality from the transcriptome sequencing on the concordance between the

two data sources. For the 67 animals, around 30% of the SNPs showed a high concordance (>90% matching rate between whole-genome sequencing and RNA data source). After removing SNPs from the transcriptome sequencing data with a call rate < 90%, considerably increased the percentage of SNPs (around 65.32%) that showed a high concordance the two data sources (>90%, Supplementary Fig. S11). Eventually, we remove SNPs with a call rate < 90%, and SNPs on the sex chromosomes, 1,967,661 SNPs were kept for subsequent analysis.

eQTL mapping

MatrixEQTL (RRID:SCR_025513) was used to carry out the eQTL analysis for the ovary tissue using the following model [43],

$$\mathbf{y} = \mathbf{X_2 b_2} + \mathbf{j}\alpha + \mathbf{e}$$

where $\mathbf{y}$ was the TPM from the ovary tissue per gene, $\mathbf{b_2}$ was a vector of the fixed effects including genetic group, age, and probabilistic estimation of expression residuals (PEER) effects calculated from R-package “peer” (RRID:SCR_009326) [44], where both genetic group, age and PEER were modeled as a linear regression, $\mathbf{X_2}$ was the corresponding design matrix, $\mathbf{j}$ wa-s a vector with allele counts (coded as 0, 1, and 2 when homozygote for the reference allele, heterozygote, and homozygote for the alternative allele, respectively); and α is the additive SNP effect. We used 10 PEER factors, as the diagnostic plot of the factor relevance showed a very similar pattern for the variance beyond the 10th factor (Supplementary Fig. S12). The *cis*-eQTL mapping window was defined from 1 megabase (Mb) upstream/downstream of the transcription start site; all other SNP-expression combinations were defined as *trans* associated. For both *cis*- and *trans*-eQTL, we applied the *P*-values threshold that corresponds to FDR < 0.01.

Transcriptome-wide association study and Mendelian randomization analysis

We corrected gene expression data for age, genetic group, and PEERs factors, and the residuals were used as the response variable in TWAS analysis. With S-PrediXcan [45], we first estimated the weights and covariance matrices of the SNPs within each gene to build a gene expression prediction model. Second, we estimated the associations between predicted gene expression levels and the traits using the GWAS summary statistics and gene expression prediction models. The identified genes were visualized using Rldeogram [46]. Subsequently, a MR analysis was done using the software MR-JTI [47] to assess causal inference between gene expression and egg production. This analysis combined LD scores, eQTL-mapping results, and GWAS summary statistics to obtain candidate genes. The input SNPs were pruned such that only relatively independent variants (LD < 0.2) that are associated with the expression of TWAS identified genes were used. LD scores for each independent variant were obtained with GCTA [48]. Bonferroni adjustment was applied to correct for multiple testing.

Results

Variance components for cumulative egg number and egg number at different stages

Estimated phenotypic variances were equivalent between models A and AD (Table 1). Phenotypic variance increased with age for cumulative egg number, and increased from EN400 to EN600 for egg number at different stages. For cumulative egg number, the additive variance explained a similar proportion of the phenotypic variance at the early and late laying stage for the two models, 56% and 57% for CEN200, 17% and 19% for

CEN300, 13% to 14% for CEN600 and CEN700. In Model AD, the dominance variance accounted for a small proportion of the total phenotypic variance at the early stages (3% for CEN200 and 6% for CEN300), a substantial proportion at the middle stages (15% for CEN400 and 23% for CEN500), and a negligible proportion at late stages, below 0.5%. For egg number at different stages, the additive variance explained a similar proportion of phenotypic variance for both models only at the early stage of laying cycle (16% and 19% for EN300). In Model AD, dominance contributed 9% to 45% of the phenotypic variance for cumulative egg number and egg number at different stages for the whole laying period.

Genome-wide association study and candidate variants

The number of significant additive and dominance SNPs detected by Model AD was 3,294 and 2,598 respectively, while no significant SNPs were detected by Model A (Supplementary Table S1). The significant SNPs were mainly located on chromosomes 1, 2, 3, 6 and 13 (Fig. 2A). Among them, 805 additive SNPs and 360 dominance SNPs were shared between multiple traits (Figs. 2B and 2C). For each trait, more than 50% of the significant SNPs were detected as additive SNPs, except for CEN700 (Fig. 2D). For the significant SNPs most of the annotated variants were intron variants, 61.65%, and for all SNPs this was 55.69% (Figs. 2E and 2F). Among the coding variants, synonymous variants are the most abundant variants, accounting for ~70% of both significant and all SNPs. While traditionally considered neutral, emerging evidence highlights the potential function of synonymous variants in mRNA stability and splicing [49]. The 3' untranslated region (3'UTR) variants were over 3-fold enriched among the significant SNPs compared to all SNPs (Supplementary Table S2), which was demonstrated to play a crucial role in post-transcriptional and translational processes [50, 51].

To further explore the advantages of incorporating dominance into GWAS model to identify the trait-related variants, we examined estimated SNP effects from the different models. The detected additive SNP effects located at chromosome 2 of CEN700 from A and AD models were clearly correlated to each other (Fig. 3A), and a subset of SNPs with extreme effects in Model AD were regarded as significant ones (Fig. 3B). The pattern was similar for other chromosomes and traits. For significant SNPs, most of the estimated additive SNP effects are negative, while most of the estimated dominance effects are positive (Fig. 3C). The additive and dominance SNP effects are in opposite directions for approximately 60% of all SNPs. (Fig. 3D), and this proportion is even higher for significant SNPs (Fig. 3E). Moreover, 251, 20, and 958 SNPs were significant for additive SNP and dominance SNP effects at the same time in traits CEN600, CEN700, and EN500 (Fig. 3F). If we trimmed the significant SNPs based on LD with $r^2 \leq 0.2$ via the Plink command --indep-pairwise 50 5 0.2, in total 132 independent genomic regions related to egg production traits were identified.

eQTL mapping results

After removing genes with TPM values equal to 0 in all samples, expression data from 28,126 genes were kept for eQTL mapping analysis. The eQTL analysis assessed associations between 1,967,661 SNPs and expression of 28,126 genes, of which 63,161,730 were *cis*-SNP-expression combinations, and 55,279,271,556 were *trans*-SNP-expression combinations (Fig. 4A). At the threshold $P < 1.12E-04$ (FDR < 0.01), we identified 704,987 significant *cis*-SNP-expression combinations, corresponding to 440,374 *cis*-acting SNPs, and 11,129 *cis*-eQTL-associated genes. At the threshold $P < 3.68E-07$ (FDR<0.01), we identified 2,035,416 significant *trans*-SNP-expression combinations, corresponding to 516,993 *trans*-acting SNPs, and 19,106 *trans*-eQTL-associated genes.

An eQTL can influence the expression of multiple genes, which is denoted as pleiotropy of eQTL [52]. Descriptive statistics revealed that 37.20% (163,815 out of 440,374) of the *cis*-eQTL and 46.55% (240,662 out of 516,993) of the *trans*-eQTL were associated with the expression of two or more genes, and 37 *cis*-eQTL and 63,608 *trans*-eQTL were associated with the expression of more than ten genes (Fig. 4B). It appeared that the *cis*-eQTL that displayed pleiotropy were distributed in specific regions on chromosomes 4, 19 and 29, which could be qualified as eQTL hotspots (Fig. 4C).

TWAS identify 298 unique genes for egg production traits

We performed TWAS analysis using S-PrediXcan, revealing 742 statistically significant gene expression-egg number associations, comprising 298 genes, whose imputed expression is associated with cumulative egg number and egg number at different stages (Supplementary Table S3). Using the RIdeogram for visualization, we found several genes affecting multiple egg production traits on chromosomes 2, 4, 6, 15 and 21 (Fig.5A) and chromosomes 1, 3, 5, 17 and 27 (Supplementary Fig. S13). Gene expression significantly associated with multiple egg production traits, especially at the middle stage of the laying period (Fig. 5B). Across different ages, we found that *ENSGALG00015002757* influenced egg production throughout the whole laying period, affecting traits such as EN300, EN400, CEN400, CEN500, CEN600, CEN700, and EN300_500 (Fig. 5B and Supplementary Table S3). In contrast, *ENSGALG00015027755* played a role primarily in early stage of laying period (CEN300, CEN400, EN300, EN400, Fig. 5B and Supplementary Table S3), while *ENSGALG00015009997* was associated with late stage of laying period (CEN600, CEN700, EN600, EN700, and EN500_700, Fig. 5B and Supplementary Table S3). Across different models, 416 significant expression of gene-egg number combinations were found in Model A, 280 significant expression of gene-egg number combinations

for additive effects and 46 significant expression of gene-egg number combinations for dominance effects in Model AD (Fig. 5C, and Supplementary Table S3). We further performed a MR analysis in order to test for causality using MR-JTI and to prioritize the genes identified by TWAS. We identified 125 candidate genes (Supplementary Table S4).

Multi-omics data analysis for egg production traits

Significant SNPs detected in the GWAS results (Table 2, Fig. 6A, and Supplementary Figs. S14-S16A) were associated with 27 genes in *cis*-SNP-gene associations identified in the eQTL analysis (Table 2, Fig. 6B, Supplementary Figs. S14-S16B, and Table S5), among which four genes were also detected by TWAS, being *ENSGALG00015011893*, *ENSGALG00015011943*, *ENSGALG00015026475,* and *ENSGALG00015025721* (Table 2, Fig. 6C, and Supplementary Figs. S14-S16C). Further causal inference with MR analysis showed that the four genes were potential causal genes for egg production (Table 2, and Supplementary Table S4). *ENSGALG00015011893* is located on chromosome 2, and the expression level was significantly associated with SNPs 2:110550769, 2:110551030, 2:110740536 and 2:110740655 (*P*-values ranged from 3.18E-08 to 1.91E-05, Table 2 and Fig. 6B), and was associated with CEN700 with Z-score -2.72 (corresponding *P* = 6.46E-03, Table 2, Fig. 6C, and Supplementary Table S3). The expression level of *ENSGALG00015011943* was associated with SNP 2:110551030 of trait CEN500 (*P* = 9.86E-06, Table 2, and Supplementary Fig. S14B), and was associated with CEN500 with Z-score -2.99 (corresponding *P* = 2.76E-03, Table 2, Supplementary Fig. S14C, and Supplementary Table S3). Similarly, the expression level of *ENSGALG00015026475* was associated with five SNPs locating on chromosome 15 (*P*-values ranged from 1.09E-07 to 1.15E-07, Table 2, and Supplementary Fig. S15B), and was associated with EN500 with Z-score -2.80

(corresponding *P* = 5.04E-03, Table 2, Supplementary Fig. S15C, and Supplementary Table S3). The expression level of *ENSGALG00015025721* was associated with six SNPs locating on chromosome 21 (*P*-values ranged from 4.32E-07 to 2.54E-06, Table 2, and Supplementary Fig. S16B), and was associated with EN500 with Z-score 2.91 (corresponding *P* = 3.63E-03, Table 2, Supplementary Fig. S16C, and Supplementary Table S3).

In addition to candidate genes, we also pinpointed several candidate SNPs, though they are not the leading SNPs in GWAS results, suggesting the leading SNPs may not function as expression quantitative trait locus, but instead influence egg number through other mechanisms. For SNP 2:110740655, reference allele C and alternative allele A, cumulative egg number and egg number at different stages of genotype CA was higher than genotype CC, and the expression of *ENSGALG00015011893* for genotype AA was the highest (Fig. 7). For SNP 2:110551030, the egg number of genotype GG at different stages was smaller than the genotype GA (Supplementary Fig. S17A), and the expression of *ENSGALG00015011943* for genotype GG was lower than the genotype GA (Supplementary Fig. S17B). For SNP 15:450945, the egg number of genotype GG at different stages was smaller than the genotype GA (Supplementary Fig. S17C), and the expression of *ENSGALG00015026475* for genotype GG was lower than the genotype GA (Supplementary Fig. S17D). For SNP 21:940575, the egg number of genotype AA at different stages was smaller than the genotype GA (Supplementary Fig. S17E), and the expression of *ENSGALG00015025721* for genotype AA was the highest (Supplementary Fig. S17F).

Implications for heterosis

Observed heterosis from the predicted mean phenotypes of each genetic group increased across ages for cumulative egg number from 1.04% to 11.51%, except for

CEN200. For egg number at different stages, heterosis increased from -3.22% to 29.48% (Table 3). Leveraging Model AD enables the calculation of SNP ratios to assess the relative importance of dominance compared to additive SNP effects. The ratios for most of the significant SNPs were higher than 0.8 (Fig. 8A red dash line), and smaller than 1.2 (Fig. 8A blue dash line), suggesting complete-dominance. Across traits, we observed a positive correlation coefficient of 0.45 (P = 0.31) between SNP ratios and heterosis (Fig. 8A). Similarly, a positive and significant correlation of 0.72 was observed between the sum of dy^2 across all SNPs and heterosis, with P = 0.0053 (Fig. 8B). Finally, a positive but insignificant correlation of 0.60 was observed for significant SNPs between the sum of dy^2 and the heterosis, with P = 0.15 (Fig. 8B).

Discussion

In the current study, by incorporating dominance SNP effects into the GWAS model and using whole-genome sequencing data for a complete double-crossed hybrid population of 1,004 chickens, we successfully identified genetic variants related to egg production traits. Egg production remains the most important trait for laying hens, despite the recent expansion of breeding goals to include health and welfare related traits [53]. In total, the eQTL-mapping analysis identified 704,987 *cis*-SNP-expression combinations and 2,035,416 *trans*-SNP-expression combinations, and TWAS analysis identified 298 genes. By carrying out the multi-omics data analysis, four novel candidate genes for egg production traits were identified. Moreover, observed heterosis positively related to the heterosis predicted based on the estimated dominance SNP effects and allele frequencies, and average ratios of dominance to additive effects.

The traditional GWAS analysis in livestock normally focus on estimating additive effects, while non-additive effects, such as dominance, are frequently overlooked.

When non-additive effects actually exist but are not modelled, they may end up partly in the residual effect and partly in the additive effect, leading to bias in the estimation of additive effects [54]. Additionally, incorporating dominance SNP effects is justified by the fact that a substantial portion of genetic variance, especially in mixed populations of purebred and crossbred animals, is likely explained by non-additive effects [55]. In the current study, with only additive SNP effects in Model A, no significant SNPs were found, while by adding dominance SNP effects in Model AD, several signals appeared across traits (Supplementary Figs. S18-S21). This finding agrees with previous research that emphasizes the role of including dominance effects in the model for enhancing the detection of associations [15, 56], and in particular when using crossbred data [9]. To further explore the advantages of incorporating dominance into GWAS model, we analyzed SNP effects in both models. Additive SNP effects were strongly correlated between the models, indicating that additive SNP effects remained largely unchanged in both models, while significant SNPs exhibited more extreme effects in Model AD. In Model A, we suspected that opposing additive and dominance SNP effects can potentially cancel each other out, as allele substitution effects as estimated in Model A are defined as $a + (q - p)d$, where a is the additive effect, d the dominance effect, and q and p are the allele frequencies [18]. This was supported by Fig. 3B, which showed that significant SNPs on chromosome 2 of CEN700 exhibited negative additive effects and positive dominance effects, appearing to offset each other. Model AD distinguished between additive and dominance SNP effects, leading to the identification of more variants in our population, which consists of purebred and crossbred animals. However, this finding cannot be generalized and will depend upon if traits are affected by dominance. For example, a study in Large White pigs reported different results across traits, with substantial dominance SNP effects observed for age at 100 kg, while no

significant dominance SNP effects were detected for backfat thickness at 100 kg [57]. Our findings highlight the importance of including dominance effects in GWAS to improve the accuracy and power to detect egg production-related variants, especially in studies involving crossbred animals.

In the current study we identified a large number of trait-related variants, possibly due to the use of whole-genome sequencing data. Most GWAS studies in chickens have relied on genotype data obtained from SNP chips, which include only a fraction of all the variants segregating in the whole chicken genome. In contrast, whole-genome sequencing data encompass nearly all genomic variants, which can enhance the effectiveness of GWAS in identifying causal mutations for quantitative traits [58]. Taken together, incorporating dominance into the GWAS model proved beneficial for identifying genetic variants for egg production traits of layers in our full diallel cross with purebred and crossbred animals with whole-genome sequencing data.

We used the reference genome of the WW line (White Leghorn), a breed known for high egg production, and the number of alleles for the alternative allele were counted (coded as 0, 1 or 2). Given this coding, and the superiority of WW over YY, we expected a higher frequency of the reference allele in WW and a higher frequency of the alternative allele in YY and therefore this coding may lead to more negative than positive additive SNP effects. Additionally, in our previous study [22], we found substantial heterosis for crossbreds which could result from the positive dominance effects. The negative correlation observed for additive and dominance SNP effects in Model AD may change or even disappear if a different allele coding would have been applied. In other words, the general relationship between the two SNP effects is that higher additive SNP effects correspond to higher absolute dominance SNP effects. This can be explained by the phenomenon in which the heterozygote for the significant SNPs

exhibits a phenotype which is similar to the phenotype of the best homozygote (Supplementary Fig. S22). This indicates that the presence of a specific allele, whether in a heterozygous or homozygous state, results in similar phenotype (i.e. complete-dominance), leading to Fig. 3C where absolute values of estimated additive and dominance have equal size. These findings underscore the importance of carefully considering the coding of alleles when interpreting estimated additive effects.

Incorporating dominance effects into the GWAS model also provides insights into the relative importance SNP effects through the ratio of dominance to additive SNP effects. By integrating the SNP ratio and estimated significant *cis*-eQTLs and *trans*-eQTLs, we showed genes associated to SNPs filtered by gene action (additive, partial-dominance, complete-dominance, and over-dominance, Supplementary Figs. S23 and S24). We observed comparable proportions of *cis*-eQTLs and *trans*-eQTLs for additive, partial-dominance, complete-dominance, and over-dominance gene actions, even after pruning SNPs based on LD. This indicated that *cis*-acting and *trans*-acting contribute equally to SNPs of additive, partial-dominance, complete-dominance, and over-dominance gene action. In contrast, Cui et al. found that genes associated with SNPs of additive gene action are mainly *cis*-acting, and genes associated with SNPs of dominant gene action are mainly *trans*-acting [9]. The higher SNP density in the current study, along with differences in the methods used to estimate additive and dominance SNP effects, may explain the observed discrepancies.

In the current study, integrating GWAS, eQTL-mapping analysis, and TWAS, we ultimately discovered four novel genes with potential causal roles in influencing egg production. *ENSGALG00015011893* is a novel gene potentially mapped to *LOC421125* in NCBI dataset, and annotated as *TMEM68-like* (transmembrane protein 68-like). *TMEM68* has been identified as an enzyme involved in lipid metabolism [59], which

may be crucial for the formation of yolk. *ENSGALG00015011943*, annotated as *TGS1* (trimethylguanosine synthase 1), was also reported as a differential expressed gene in pre-recruitment and pre-ovulatory follicles [60], indicating a potential role in regulating the egg producing process. Notably, SNP 2:110551030 was associated with the expression of both genes for cumulative egg number till different ages, illustrating its pleiotropic effects. Despite the absence of direct regulatory annotation in our reference genome, the significant eQTL association between this SNP and both genes supports the hypothesis that the variant may have a regulatory function or serve as proxy for causal variants within regulatory elements. This integrated analysis of genomic and transcriptome data helps to identify trait-related genetic variants and genes, showing that multi-omics data can contribute to deciphering genetic mechanisms underlying egg production by establishing connections between genetic variants, gene expression, and egg number.

In addition to identifying candidate genetic variants related to egg production traits, we also explored implications regarding heterosis utilizing estimated dominance SNP effects, given that chicken is one of the most well-known animals where hybrid vigor, or heterosis, is leveraged in commercial populations. Following the methods described previously [9, 22], we calculated observed heterosis from predicted means for genetic groups of egg production traits, and assessed the extent of dominance effects per SNP through SNP ratios. Ratios of significant SNP identified by GWAS generally ranged from 0.8 to 1.2, with the mean value 1.00 (Supplementary Table S6), suggesting a complete-dominance gene action [9]. Based on these results, complete-dominance is expected to be the predominant pattern in egg production heterosis. We did not find other studies in animals attempted to investigated this, but this observation aligns with previous reports in plants [61, 62]. It should be noted that in our study the observed

correlations between SNP ratio and heterosis could be affected by the fact that the traits and thus also the significant SNPs across traits are highly related, or by the limited number of traits considered. Nevertheless, we did observe a positive correlation between SNP ratio and heterosis, consistent with the theory and the expectation, stating that with increasing extent of dominance compared to additive effects, a larger amount of heterosis is expected. Moreover, we observed a lower correlation between heterosis and the SNP ratios for all compared to only the significantly detected SNPs (Supplementary Fig. S25).

In addition to the SNP ratios, we considered the quantitative genetics theory that the amount of heterosis depends on dy^2, i.e. the product of the dominance effects multiplied by the squared difference in allele frequency [18]. We observed a positive and significant correlation between the sum of dy^2 and heterosis for all SNPs, but an insignificant correlation for significant SNPs. Given the similar correlation of 0.72 for all SNPs and 0.60 for significant SNPs, we argue that the limited number of significant SNPs still had an important impact on phenotypes. In line with this, Amuzu-Aweh et al. reported an accuracy of ~0.5 for predicting heterosis using the genome-wide squared difference in allele frequency between parental pure lines [63]. Apart from the implications to heterosis, we also observed differences between reciprocal crosses for egg production traits, and also in other egg-laying traits [22]. However, we could not unambiguously determine a cause of these reciprocal differences due to the confounding effects between sex-linked genes and genetic group in our dataset. Other plausible explanations for differences between the reciprocal crosses include parent-of-origin effects or different breed origin of the mitochondrial DNA [64, 65].

Conclusion

In the current study, we identified 5,982 SNPs and three candidate genes for egg production traits by analyzing 1,004 fully sequenced layers. These results suggest that incorporating dominance into the GWAS model not only help to detect the variants for egg production traits of layers in a mixed population with purebred and crossbred animals, but also demonstrates that traits with higher heterosis tended to be more affected by genes with dominant mode of action. Moreover, multi-omics data allows to contribute to deciphering genetic mechanisms underlying egg production by establishing connections between genetic variants, gene expression, and egg number.

Additional Files

Supplementary Fig. S1. Mapping quality of 1,004 whole-genome sequenced animals

Supplementary Fig. S2. Scatter plot for pedigree and genome coefficients for crossbreds

Supplementary Fig. S3. Scatter plot for pedigree and genome coefficients for crossbreds after removing conflict animals

Supplementary Fig. S4. Scatter plot for pedigree and genome coefficients for WW

Supplementary Fig. S5. Scatter plot for pedigree and genome coefficients for WW after removing conflict animals

Supplementary Fig. S6. Scatter plot for pedigree and genome coefficients for YY

Supplementary Fig. S7. Scatter plot for pedigree and genome coefficients for YY after removing conflict animals

Supplementary Fig. S8. PCA plot for the four genetic groups

Supplementary Fig. S9. Mapping quality of transcriptome data

Supplementary Fig. S10. Distribution of concordance for individual and SNPs for different imputation methods.

Supplementary Fig. S11. Distribution of concordance for SNPs when applying missing genotype smaller than 10%.

Supplementary Fig. S12. Diagnostic plot of the factor relevance (automatic relevance determination parameters).

Supplementary Fig. S13. Genes identified by TWAS analysis

Supplementary Fig. S14. Multi-omics data analysis of genetic determinants underlying cumulative egg number till 500 days of age for additive SNP effects.

Supplementary Fig. S15. Multi-omics data analysis of genetic determinants underlying egg number between 400 and 500 days of age for additive SNP effects.

Supplementary Fig. S16. Multi-omics data analysis of genetic determinants underlying egg number between 400 and 500 days of age for dominance SNP effects.

Supplementary Fig. S17. The correlations between candidate variants and phenotype, and expression of candidate genes

Supplementary Fig. S18. QQplots across traits

Supplementary Fig. S19. Manhattan plots of additive SNP effects for cumulative egg number and egg number at different stages in Model A.

Supplementary Fig. S20. Manhattan plots of additive SNP effects for cumulative egg number and egg number at different stages in Model AD.

Supplementary Fig. S21. Manhattan plots of dominance SNP effects for cumulative

egg number and egg number at different stages in Model AD.

Supplementary Fig. S22. Proportion of significant SNPs where heterozygotes exhibit a phenotype similar to the phenotype of the best homozygote.

Supplementary Fig. S23. *Cis*- and *trans*-acting eQTLs and their associated genes.

Supplementary Fig. S24. *Cis*- and *trans*-acting eQTLs and their associated genes after pruning with linkage equilibrium.

Supplementary Fig. S25. Correlation between SNP ratios of all SNPs and heterosis

Supplementary Table S1. Significant SNPs identified by GWAS

Supplementary Table S2. Annotation of significant and all SNPs

Supplementary Table S3. Significant genes identified by TWAS

Supplementary Table S4. MR analyze for candidate genes identified by TWAS

Supplementary Table S5. Expression level of 27 genes associated with significant SNPs identified by GWAS

Supplementary Table S6. Ratio values of significant SNPs

Data Availability

The genomic and transcriptomic sequence data generated in this study are available under the BioProject accessions: PRJCA032894 in the National Genomics Data Center (NGDC) database and PRJEB82328 and PRJEB88001 in the European Nucleotide Archive (ENA) database. All additional supporting data are available in the GigaScience repository, GigaDB [66]. The software code is available in SoftwareHeritage [67].

Availability of source code and requirements

Project name: Identifying candidate genetic variants for egg number by analyzing over 1000 fully sequenced layer project

Project home page: https://github.com/aixin951/EP_WGS_1004_layers

Operating system(s): Platform independent

Programming language: R

Other requirements: R 4.2.3 or higher

License: MIT

Software Heritage PID: swh:1:snp:abd33c288a589ee4a1aecd5f12238479c6c98117

List of abbreviations

SNP: single nucleotide polymorphisms; GWAS: genome-wide association study; TWAS: transcriptome-wide association study; eQTL: expression quantitative trait loci; QTL: quantitative trait loci; MR: Mendelian randomization; dy^2: the sum of the dominance effects multiplied by the squared difference in allele frequency between the parental lines; YY: Beijing-You chickens; WW: White Leghorn chickens; WY: offspring of a cross between White Leghorn as the sire line and Beijing-You as the dam line; YW: offspring of a cross between Beijing-You as the sire line and White Leghorn as the dam line; CEN200: cumulative egg number till 200 days of age; CEN300: cumulative egg number till 300 days of age; CEN400: cumulative egg number till 400 days of age; CEN500: cumulative egg number till 500 days of age; CEN600: cumulative egg number till 600 days of age; CEN700: cumulative egg number till 700 days of age; EN300: egg number from 200 to 300 days of age; EN400: egg number

from 300 to 400 days of age; EN500: egg number from 400 to 500 days of age; EN600: egg number from 500 to 600 days of age; EN700: egg number from 600 to 700 days of age; EN300_500: egg number from 300 to 500 days of age; EN500_700: egg number from 500 to 700 days of age; Model A: additive model; Model AD: additive-dominance model; FDR: false discovery rate; VEP: variant effect predictor; TPM: transcripts per kilobase per million mapped reads; Mb: megabase; LD: linkage disequilibrium; 3'UTR: 3' untranslated region; *TMEM68*: transmembrane protein 68; *TGS1*: trimethylguanosine synthase 1; *DNAH10*: dynein axonemal heavy chain 10; *CEP104*: centrosomal protein 104.

Declarations

Ethical approval

The study was approved by the Animal Care and Use Committee of the Institute of Animal Science, Chinese Academy of Agricultural Sciences (No. IAS2021-48), where the experiments were conducted. All experiments were performed in accordance with the relevant guidelines and regulations set by Ministry of Agriculture and Rural Affairs of the People's Republic of China.

Consent for publication

Not applicable

Competing interests

The author(s) declare that they have no competing interests.

Funding

This work was supported by the National Natural Science Foundation of China

(32172721), Chinese Agricultural Research System (CARS-40), and the Agricultural Science and Technology Innovation Program (ASTIP-2021-IAS-16).

Authors' contributions

J.C. and Y.S. conceived the initial study design. H.B., M.P.L.C., Y.S., and J.C., discussed, specified the final study design and supervised the work, A.N., Y.L., and J.Y. collected the phenotype data. A.N. performed the bioinformatic analysis, and wrote the first version of the manuscript. Y.L., and J.Y. contributed to statistical analyses. A.N., H.B., M.P.L.C., Y.L., Y.S. and J.C. provided valuable insights throughout the writing process. All authors read and approved the final manuscript.

Acknowledgements

The authors extend their gratitude to Chao Chen (Institute of Animal Science, Chinese Academy of Agricultural Sciences, Beijing, China) for his assistance with raising animals. The authors also acknowledge Martijn Derks and Carolina Barros (Animal Breeding and Genomics Group, Wageningen University and Research, the Netherlands) for their help in SNP calling and gene annotations.

Reference

1. Statista: Global egg production from 1990 to 2022. https://www.statista.com/statistics/263972/egg-production-worldwide-since-1990/ (2024). Accessed February 2024.
2. Bain MM, Nys Y and Dunn IC. Increasing persistency in lay and stabilising egg quality in longer laying cycles. What are the challenges? *Br Poult Sci*. 2016;57(3):330-8. doi:10.1080/00071668.2016.1161727.
3. ChickenQTLdb. https://www.animalgenome.org/cgi-bin/QTLdb/GG/index. Accessed May 2025.
4. Uffelmann E, Huang QQ, Munung NS, de Vries J, Okada Y, Martin AR, et al. Genome-wide association studies. *Nature Reviews Methods Primers*. 2021;1(1):59. doi:10.1038/s43586-021-00056-9.
5. Cai W, Zhang Y, Chang T, Wang Z, Zhu B, Chen Y, et al. The eQTL colocalization and transcriptome-wide association study identify potentially

causal genes responsible for economic traits in Simmental beef cattle. *Journal of Animal Science and Biotechnology*. 2023;14(1):78. doi:10.1186/s40104-023-00876-7.

6. Liu X, Zhang J, Xiong X, Chen C, Xing Y, Duan Y, et al. An Integrative Analysis of Transcriptome and GWAS Data to Identify Potential Candidate Genes Influencing Meat Quality Traits in Pigs. *Frontiers in Genetics*. 2021;12:748070. doi:10.3389/fgene.2021.748070.
7. Teng J, Gao Y, Yin H, Bai Z, Liu S, Zeng H, et al. A compendium of genetic regulatory effects across pig tissues. *Nat Genet*. 2024;56(1):112-23. doi:10.1038/s41588-023-01585-7.
8. Guan D, Bai Z, Zhu X, Zhong C, Hou Y, Lan F, et al. The ChickenGTEx pilot analysis: a reference of regulatory variants across 28 chicken tissues. *bioRxiv*. 2023;06. doi:10.1101/2023.06.27.546670.
9. Cui L, Yang B, Xiao S, Gao J, Baud A, Graham D, et al. Dominance is common in mammals and is associated with trans-acting gene expression and alternative splicing. *Genome Biol*. 2023;24(1):215. doi:10.1186/s13059-023-03060-2.
10. Liu Y, Xu L, Wang Z, Xu L, Chen Y, Zhang L, et al. Genomic Prediction and Association Analysis with Models Including Dominance Effects for Important Traits in Chinese Simmental Beef Cattle. *Animals (Basel)*. 2019;9(12):1055. doi:10.3390/ani9121055.
11. Ebrahimi K, Dashab GR, Faraji-Arough H and Rokouei M. Estimation of additive and non-additive genetic variances of body weight in crossbreed populations of the Japanese quail. *Poult Sci*. 2019;98(1):46-55. doi:10.3382/ps/pey357.
12. Alves K, Brito LF, Baes CF, Sargolzaei M, Robinson JAB and Schenkel FS. Estimation of additive and non-additive genetic effects for fertility and reproduction traits in North American Holstein cattle using genomic information. *J Anim Breed Genet*. 2020;137(3):316-30. doi:10.1111/jbg.12466.
13. Joshi R, Meuwissen THE, Woolliams JA and Gjoen HM. Genomic dissection of maternal, additive and non-additive genetic effects for growth and carcass traits in Nile tilapia. *Genet Sel Evol*. 2020;52(1):1. doi:10.1186/s12711-019-0522-2.
14. Amuzu-Aweh EN. *Genomics of heterosis and egg production in White Leghorns*. Wageningen University and Research, 2020. doi:10.18174/508397.
15. Nagai R, Kinukawa M, Watanabe T, Ogino A, Kurogi K, Adachi K, et al. Genome-wide detection of non-additive quantitative trait loci for semen production traits in beef and dairy bulls. *Animal*. 2022;16(3):100472. doi:10.1016/j.animal.2022.100472.
16. Estrada-Reyes ZM, Rae DO and Mateescu RG. Genome-wide scan reveals important additive and non-additive genetic effects associated with resistance to Haemonchus contortus in Florida Native sheep. *Int J Parasitol*. 2021;51(7):535-43. doi:10.1016/j.ijpara.2020.11.003.
17. Tarsani E, Kranis A, Maniatis G, Avendano S, Hager-Theodorides AL and Kominakis A. Deciphering the mode of action and position of genetic variants impacting on egg number in broiler breeders. *BMC Genomics*. 2020;21(1):512. doi:10.1186/s12864-020-06915-1.
18. Falconer DS. Introduction to quantitative genetics. Pearson Education India; 1996.
19. Li D, Huang Z, Song S, Xin Y, Mao D, Lv Q, et al. Integrated analysis of

phenome, genome, and transcriptome of hybrid rice uncovered multiple heterosis-related loci for yield increase. *Proc Natl Acad Sci U S A*. 2016;113(41):E6026-E35. doi:10.1073/pnas.1610115113.

20. Wang B, Hou M, Shi J, Ku L, Song W, Li C, et al. De novo genome assembly and analyses of 12 founder inbred lines provide insights into maize heterosis. *Nat Genet*. 2023;55(2):312-23. doi:10.1038/s41588-022-01283-w.

21. Ye J, Liang H, Zhao X, Li N, Song D, Zhan J, et al. A systematic dissection in oilseed rape provides insights into the genetic architecture and molecular mechanism of yield heterosis. *Plant Biotechnol J*. 2023;21(7):1479-95. doi:10.1111/pbi.14054.

22. Ni A, Calus MPL, Bovenhuis H, Yuan J, Wang Y, Sun Y, et al. Genetic parameters, reciprocal cross differences, and age-related heterosis of egg-laying performance in chickens. *Genet Sel Evol*. 2023;55(1):87. doi:10.1186/s12711-023-00862-7.

23. Andrews S. FastQC: a quality control tool for high throughput sequence data. *Cambridge, United Kingdom*, 2010.

24. Bolger AM, Lohse M and Usadel B. Trimmomatic: a flexible trimmer for Illumina sequence data. *Bioinformatics*. 2014;30(15):2114-20. doi:10.1093/bioinformatics/btu170.

25. Li H and Durbin R. Fast and accurate short read alignment with Burrows–Wheeler transform. *bioinformatics*. 2009;25(14):1754-60. doi:10.1093/bioinformatics/btp324.

26. Faust GG and Hall IM. SAMBLASTER: fast duplicate marking and structural variant read extraction. *Bioinformatics*. 2014;30(17):2503-5. doi:10.1093/bioinformatics/btu314.

27. Li H, Handsaker B, Wysoker A, Fennell T, Ruan J, Homer N, et al. The sequence alignment/map format and SAMtools. *bioinformatics*. 2009;25(16):2078-9. doi:10.1093/bioinformatics/btp352.

28. Rhie A, McCarthy SA, Fedrigo O, Damas J, Formenti G, Koren S, et al. Towards complete and error-free genome assemblies of all vertebrate species. *Nature*. 2021;592(7856):737-46. doi:10.1038/s41586-021-03451-0.

29. Garrison E and Marth G. Haplotype-based variant detection from short-read sequencing. *arXiv preprint arXiv:12073907*. 2012. doi:10.48550/arXiv.1207.3907.

30. Bonfield JK, Marshall J, Danecek P, Li H, Ohan V, Whitwham A, et al. HTSlib: C library for reading/writing high-throughput sequencing data. *Gigascience*. 2021;10(2):giab007. doi:10.1093/gigascience/giab007.

31. Okonechnikov K, Conesa A and García-Alcalde F. Qualimap 2: advanced multi-sample quality control for high-throughput sequencing data. *Bioinformatics*. 2016;32(2):292-4. doi:10.1093/bioinformatics/btv566.

32. Purcell S, Neale B, Todd-Brown K, Thomas L, Ferreira MA, Bender D, et al. PLINK: a tool set for whole-genome association and population-based linkage analyses. *Am J Hum Genet*. 2007;81(3):559-75. doi:10.1086/519795.

33. Browning SR and Browning BL. Rapid and accurate haplotype phasing and missing-data inference for whole-genome association studies by use of localized haplotype clustering. *Am J Hum Genet*. 2007;81(5):1084-97. doi:10.1086/521987.

34. Meyer K. WOMBAT—A tool for mixed model analyses in quantitative genetics by restricted maximum likelihood (REML). *J Zhejiang Univ Sci B*. 2007;8(11):815-21. doi:10.1631/jzus.2007.B0815.

35. VanRaden PM. Efficient methods to compute genomic predictions. *J Dairy Sci*. 2008;91(11):4414-23. doi:10.3168/jds.2007-0980.
36. Vitezica ZG, Varona L and Legarra A. On the additive and dominant variance and covariance of individuals within the genomic selection scope. *Genetics*. 2013;195(4):1223-30. doi:10.1534/genetics.113.155176.
37. Yin L, Zhang H, Tang Z, Xu J, Yin D, Zhang Z, et al. rMVP: a memory-efficient, visualization-enhanced, and parallel-accelerated tool for genome-wide association study. *Genomics, Proteomics and Bioinformatics*. 2021;19(4):619-28. doi:10.1016/j.gpb.2020.10.007.
38. McLaren W, Gil L, Hunt SE, Riat HS, Ritchie GR, Thormann A, et al. The ensembl variant effect predictor. *Genome Biol*. 2016;17(1):1-14. doi:10.1186/s13059-016-0974-4.
39. Yuan J, Zhao J, Sun Y, Wang Y, Li Y, Ni A, et al. The mRNA-lncRNA landscape of multiple tissues uncovers key regulators and molecular pathways that underlie heterosis for feed intake and efficiency in laying chickens. *Genet Sel Evol*. 2023;55(1):69. doi:10.1186/s12711-023-00834-x.
40. Dobin A, Davis CA, Schlesinger F, Drenkow J, Zaleski C, Jha S, et al. STAR: ultrafast universal RNA-seq aligner. *Bioinformatics*. 2013;29(1):15-21. doi:10.1093/bioinformatics/bts635.
41. Institute B. Picard: a set of Java command line tools for manipulating high-throughput sequencing data (HTS) data and formats.: *GitHub Repository*, 2020. http://broadinstitute.github.io/picard
42. Van der Auwera GA and O'Connor BD. Genomics in the cloud: using Docker, GATK, and WDL in Terra. O'Reilly Media; 2020.
43. Shabalin AA. Matrix eQTL: ultra fast eQTL analysis via large matrix operations. *Bioinformatics*. 2012;28(10):1353-8. doi:10.1093/bioinformatics/bts163.
44. Stegle O, Parts L, Piipari M, Winn J and Durbin R. Using probabilistic estimation of expression residuals (PEER) to obtain increased power and interpretability of gene expression analyses. *Nat Protoc*. 2012;7(3):500-7. doi:10.1038/nprot.2011.457.
45. Barbeira AN, Dickinson SP, Bonazzola R, Zheng J, Wheeler HE, Torres JM, et al. Exploring the phenotypic consequences of tissue specific gene expression variation inferred from GWAS summary statistics. *Nature Communications*. 2018;9(1):1825. doi:10.1038/s41467-018-03621-1.
46. Hao Z, Lv D, Ge Y, Shi J, Weijers D, Yu G, et al. RIdeogram: drawing SVG graphics to visualize and map genome-wide data on the idiograms. *PeerJ Computer Science*. 2020;6:e251. doi:10.7717/peerj-cs.251.
47. Zhou D, Jiang Y, Zhong X, Cox NJ, Liu C and Gamazon ER. A unified framework for joint-tissue transcriptome-wide association and Mendelian randomization analysis. *Nat Genet*. 2020;52(11):1239-46. doi:10.1038/s41588-020-0706-2.
48. Yang J, Lee SH, Goddard ME and Visscher PM. GCTA: a tool for genome-wide complex trait analysis. *Am J Hum Genet*. 2011;88(1):76-82. doi:10.1016/j.ajhg.2010.11.011.
49. Shen X, Song S, Li C and Zhang J. Synonymous mutations in representative yeast genes are mostly strongly non-neutral. *Nature*. 2022;606(7915):725-31. doi:10.1038/s41586-022-04823-w.
50. Griesemer D, Xue JR, Reilly SK, Ulirsch JC, Kukreja K, Davis JR, et al. Genome-wide functional screen of 3'UTR variants uncovers causal variants for human disease and evolution. *Cell*. 2021;184(20):5247-60.

doi:10.1016/j.cell.2021.08.025.

51. Mayr C and Bartel DP. Widespread Shortening of 3′UTRs by Alternative Cleavage and Polyadenylation Activates Oncogenes in Cancer Cells. *Cell*. 2009;138(4):673-84. doi:10.1016/j.cell.2009.06.016.

52. Tian J, Keller MP, Broman AT, Kendziorski C, Yandell BS, Attie AD, et al. The Dissection of Expression Quantitative Trait Locus Hotspots. *Genetics*. 2016;202(4):1563-74. doi:10.1534/genetics.115.183624.

53. Wolc A. Poultry breeding programs in XXI century and beyond. *65-LECIE KOMITETU NAUK ZOOTECHNICZNYCH I AKWAKULTURY PAN*. 2022:83.

54. Duenk P, Calus MPL, Wientjes YCJ and Bijma P. Benefits of Dominance over Additive Models for the Estimation of Average Effects in the Presence of Dominance. *G3 (Bethesda)*. 2017;7(10):3405-14. doi:10.1534/g3.117.300113.

55. Bouvet JM, Makouanzi G, Cros D and Vigneron P. Modeling additive and non-additive effects in a hybrid population using genome-wide genotyping: prediction accuracy implications. *Heredity (Edinb)*. 2016;116(2):146-57. doi:10.1038/hdy.2015.78.

56. Bolormaa S, Pryce JE, Zhang Y, Reverter A, Barendse W, Hayes BJ, et al. Non-additive genetic variation in growth, carcass and fertility traits of beef cattle. *Genet Sel Evol*. 2015;47(1):26. doi:10.1186/s12711-015-0114-8.

57. Yang W, Wu J, Yu J, Zheng X, Kang H, Wang Z, et al. A genome-wide association study reveals additive and dominance effects on growth and fatness traits in large white pigs. *Anim Genet*. 2021;52(5):749-53. doi:10.1111/age.13131.

58. Heidaritabar M, Bink M, Dervishi E, Charagu P, Huisman A and Plastow GS. Genome-wide association studies for additive and dominance effects for body composition traits in commercial crossbred Pietrain pigs. *J Anim Breed Genet*. 2023;140(4):413-30. doi:10.1111/jbg.12768.

59. Wang Y, Zeng F, Zhao Z, He L, He X, Pang H, et al. Transmembrane Protein 68 Functions as an MGAT and DGAT Enzyme for Triacylglycerol Biosynthesis. *International Journal of Molecular Sciences*. 2023;24(3):2012. doi:10.3390/ijms24032012.

60. Kramer AE, Ellwood KM, Guarino N, Li C-J and Dutta A. Transcriptomic data reveals MYC as an upstream regulator in laying hen follicular recruitment. *Poult Sci*. 2025;104(1):104547. doi:10.1016/j.psj.2024.104547.

61. Liu H, Wang Q, Chen M, Ding Y, Yang X, Liu J, et al. Genome-wide identification and analysis of heterotic loci in three maize hybrids. *Plant Biotechnol J*. 2020;18(1):185-94. doi:10.1111/pbi.13186.

62. Yang M, Wang X, Ren D, Huang H, Xu M, He G, et al. Genomic architecture of biomass heterosis in Arabidopsis. *Proc Natl Acad Sci U S A*. 2017;114(30):8101-6. doi:10.1073/pnas.1705423114.

63. Amuzu-Aweh EN, Bijma P, Kinghorn BP, Vereijken A, Visscher J, van Arendonk JA, et al. Prediction of heterosis using genome-wide SNP-marker data: application to egg production traits in white Leghorn crosses. *Heredity (Edinb)*. 2013;111(6):530-8. doi:10.1038/hdy.2013.77.

64. Tuiskula-Haavisto M, de Koning DJ, Honkatukia M, Schulman NF, Maki-Tanila A and Vilkki J. Quantitative trait loci with parent-of-origin effects in chicken. *Genet Res*. 2004;84(1):57-66. doi:10.1017/s0016672304006950.

65. Nadaf J, Pitel F, Gilbert H, Duclos MJ, Vignoles F, Beaumont C, et al. QTL for several metabolic traits map to loci controlling growth and body composition in an F2 intercross between high- and low-growth chicken lines. *Physiol Genomics*.

2009;38(3):241-9. doi:10.1152/physiolgenomics.90384.2008.

66. Ni A, Bovenhuis H, Calus MPL, Li Y, Yuan J, Sun Y, et al. Supporting data for "Identifying candidate genetic variants for egg number by analyzing over 1000 fully sequenced layers". *GigaScience Database*. 2025. doi:10.5524/102689.

67. Ni A, Bovenhuis H, Calus MPL, Li Y, Yuan J, Sun Y, et al. Identifying candidate genetic variants for egg number by analyzing over 1000 fully sequenced layers [Computer software]. *Software Heritage*, 2025. https://archive.softwareheritage.org/browse/snapshot/abd33c288a589ee4a1aecd5f12238479c6c98117

Table 1. Estimates of variances for cumulative egg number and egg number at different stages

Trait	Model A		Model AD			
	σ_a^2/σ_p^2 (%)	σ_p^2	σ_a^2/σ_p^2 (%)	σ_d^2/σ_p^2 (%)	σ_G^2/σ_p^2 (%)	σ_p^2
CEN200	57	66	56	3	59	66
CEN300	19	180	17	6	23	180
CEN400	16	426	12	15	27	431
CEN500	11	1158	7	23	29	1188
CEN600	13	2589	13	1e-2	13	2589
CEN700	14	4400	14	3e-1	14	4401
EN300	19	104	16	9	25	104
EN400	25	93	16	31	48	96
EN500	12	315	2	45	47	331
EN600	17	547	13	14	26	552
EN700	18	554	9	23	32	560
EN300_500	13	595	8	27	35	615
EN500_700	21	1732	15	19	34	1749

Model A: model with only additive genetic effects, Model AD: model with additive and dominance effects; CENX: cumulative egg number till X days of age, ENX: egg number in 100 days interval till X days of age; EN300_500: egg number between 300 and 500 days of age; EN500_700: egg number between 500 and 700 days of age.

Standard errors for phenotypic variances ranged from 4 to 274 for Model A, and from 4 to 272 for Model AD. Standard errors for additive variances ranged from 6 to 360 for Model A, and from 6 to 373 for Model AD. Standard errors for dominance variances ranged from 5 to 507 for Model AD.

Table 2. Genes associated with egg production traits in additive-dominance model for additive SNP effects

Ensembl ID	SNP	SNP_type	Trait	GWAS_pvalue	eQTL_pvalue	Z-score	MR_beta	MR_95%_CI
ENSGALG00015011893 (TMEM68-like)	2:110550769	Additive SNP effects	CEN700	1.37E-07	1.91E-05	-2.72	-0.50	(-0.51, -0.11)
	2:110551030	Additive SNP effects	CEN700	1.44E-08	1.91E-05			
	2:110740536	Additive SNP effects	CEN700	1.47E-07	3.18E-08			
	2:110740655	Additive SNP effects	CEN700	6.50E-08	3.18E-08			
ENSGALG00015011943 (TGS1)	2:110551030	Additive SNP effects	CEN500	2.48E-09	9.86E-06	-2.99	-0.47	(-0.46, 0.05)
ENSGALG00015026475 (DNAH10)	15:4509433	Additive SNP effects	EN500	1.11E-07	1.31E-06	-2.80	-0.38	(-0.58, -0.16)
	15:4509450	Additive SNP effects	EN500	1.14E-07	1.31E-06			
	15:4509495	Additive SNP effects	EN500	1.09E-07	1.31E-06			
	15:4510674	Additive SNP effects	EN500	1.15E-07	1.31E-06			
	15:4512681	Additive SNP effects	EN500	1.13E-07	1.94E-05			
ENSGALG00015025721 (CEP104)	21:876167	Dominance SNP effects	EN500	2.54E-06	3.46E-10	2.91	0.38	(0.01, 0.68)
	21:939370	Dominance SNP effects	EN500	7.47E-07	2.04E-11			
	21:940575	Dominance SNP effects	EN500	7.47E-07	2.04E-11			
	21:959428	Dominance SNP effects	EN500	4.32E-07	2.04E-11			
	21:960286	Dominance SNP effects	EN500	7.47E-07	2.04E-11			
	21:960523	Dominance SNP effects	EN500	1.68E-06	3.62E-11			

Table 3. Predicted egg production traits for each genetic group and estimates of heterosis for the crossbreds

Trait	WW	YY	WY	YW	Heterosis (%)
CEN200	38.06	14.67	28.604	14.87	12.52
CEN300	126.10	93.37	110.72	93.37	1.04
CEN400	198.80	143.47	178.49	143.5	4.63
CEN500	269.60	194.08	247.46	194.02	4.77
CEN600	320.30	229.35	304.43	229.21	8.87
CEN700	378.20	251.50	363.34	251	11.51
EN300	88.81	77.54	82.656	77.39	-3.22
EN400	71.25	50.59	66.927	50.54	8.13
EN500	68.68	47.72	68.4657	47.69	15.44
EN600	48.77	33.54	56.161	33.08	20.33
EN700	56.36	21.01	57.0768	20.59	29.48
EN300_500	143.50	99.93	136.089	99.92	11.05
EN500_700	104.80	54.36	113.232	53.28	23.13

CENX: cumulative egg number till X days of age, ENX: egg number in 100 days interval till X days of age; EN300_500: egg number between 300 and 500 days of age; EN500_700: egg number between 500 and 700 days of age.

Heterosis (%): the average percentage of performance of crossbreds being better than the average performance of the two parental lines.

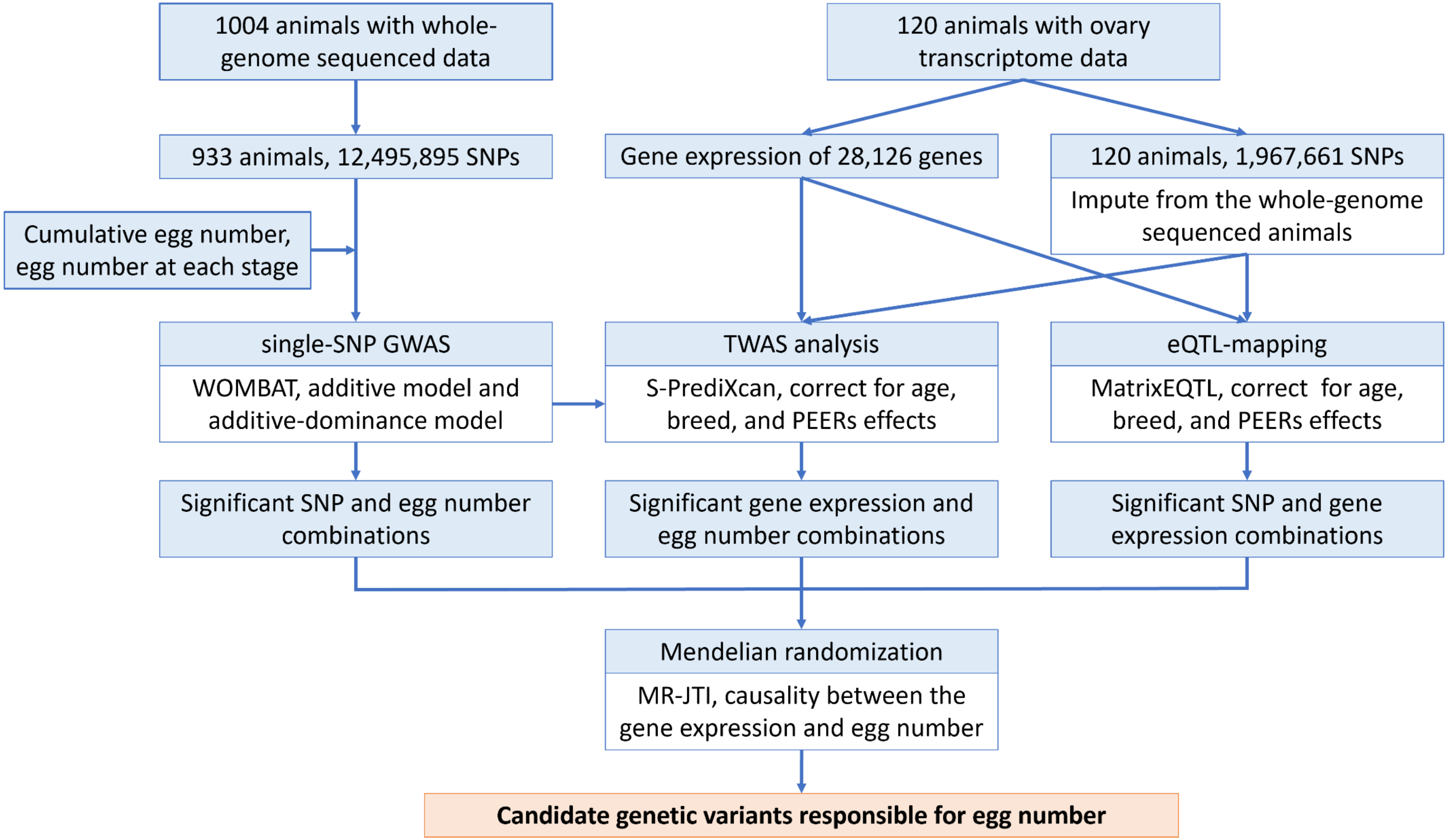
1004 animals with whole-genome sequenced data
120 animals with ovary transcriptome data
933 animals, 12,495,895 SNPs
Gene expression of 28,126 genes
120 animals, 1,967,661 SNPs
Impute from the whole-genome sequenced animals
Cumulative egg number, egg number at each stage
single-SNP GWAS
WOMBAT, additive model and additive-dominance model
TWAS analysis
S-PrediXcan, correct for age, breed, and PEERs effects
eQTL-mapping
MatrixEQTL, correct for age, breed, and PEERs effects
Significant SNP and egg number combinations
Significant gene expression and egg number combinations
Significant SNP and gene expression combinations
Mendelian randomization
MR-JTI, causality between the gene expression and egg number
Candidate genetic variants responsible for egg number

Fig. 1. Framework for identification of SNPs and genes for egg production traits using multi-omics data

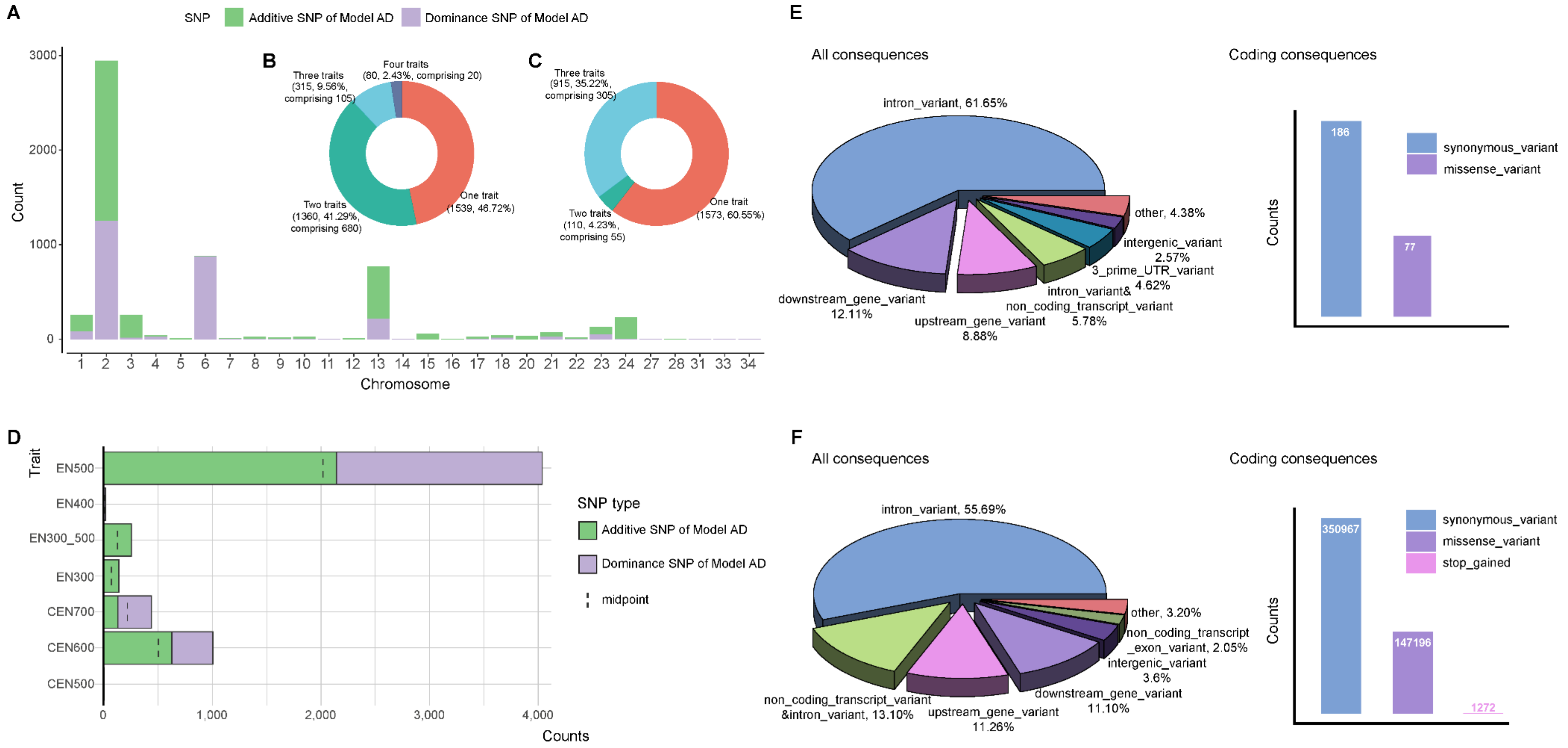

Fig. 2. Characteristics of significant SNPs. **(A)** Distribution of significant SNPs across chromosomes. **(B)** Number and percentage of significant additive SNPs in Model AD for different trait combinations. **(C)** Number and percentage of significant dominance SNPs in Model AD for different trait combinations. **(D)** Distribution of significant SNPs across traits, filtered by SNP effects. **(E)** Variant Effect Predictor (VEP) annotations of

significant SNPs. **(F)** VEP annotations of all SNPs.

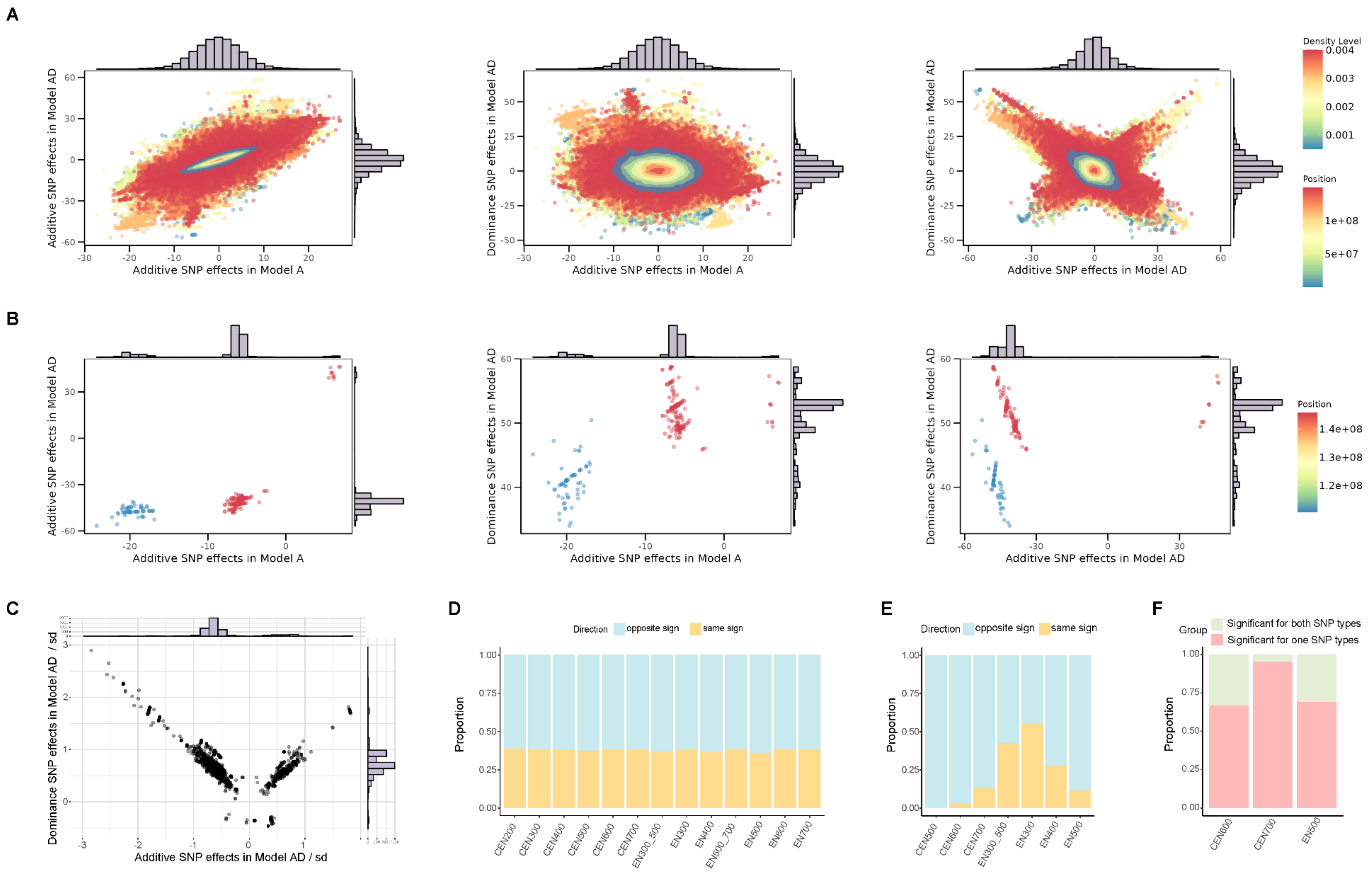

A
Additive SNP effects in Model AD
Additive SNP effects in Model A
Dominance SNP effects in Model AD
Additive SNP effects in Model A
Dominance SNP effects in Model AD
Additive SNP effects in Model AD
Density Level
0.004
0.003
0.002
0.001
Position
1e+08
5e+07
B
Additive SNP effects in Model AD
Additive SNP effects in Model A
Dominance SNP effects in Model AD
Additive SNP effects in Model A
Dominance SNP effects in Model AD
Additive SNP effects in Model AD
Position
1.4e+08
1.3e+08
1.2e+08
C
Dominance SNP effects in Model AD / sd
Additive SNP effects in Model AD / sd
D
Direction
opposite sign
same sign
Proportion
CEN200
CEN300
CEN400
CEN500
CEN600
CEN700
EN300_500
EN300
EN400
EN500_700
EN500
EN600
EN700
E
Direction
opposite sign
same sign
Proportion
CEN500
CEN600
CEN700
EN300_500
EN300
EN400
EN500
F
Group
Significant for both SNP types
Significant for one SNP types
Proportion
CEN600
CEN700
EN500

Fig. 3. Estimated additive and dominance SNP effects from an additive model and an additive-dominance model. **(A)** SNP effects for all SNPs on chromosome 2 for CEN700. **(B)** SNP effects for significant SNPs on chromosome 2 for CEN700. **(C)** Distribution of SNP effects standardized using the phenotypic standard deviation for significant SNPs across traits. **(D)** Direction of estimated additive and dominance SNP effects for all SNPs in additive-dominance model. **(E)** Direction of estimated additive and dominance SNP effects for significant SNPs in additive-dominance model. **(F)** SNPs significant for both additive and dominance as a proportion of total significant SNPs.

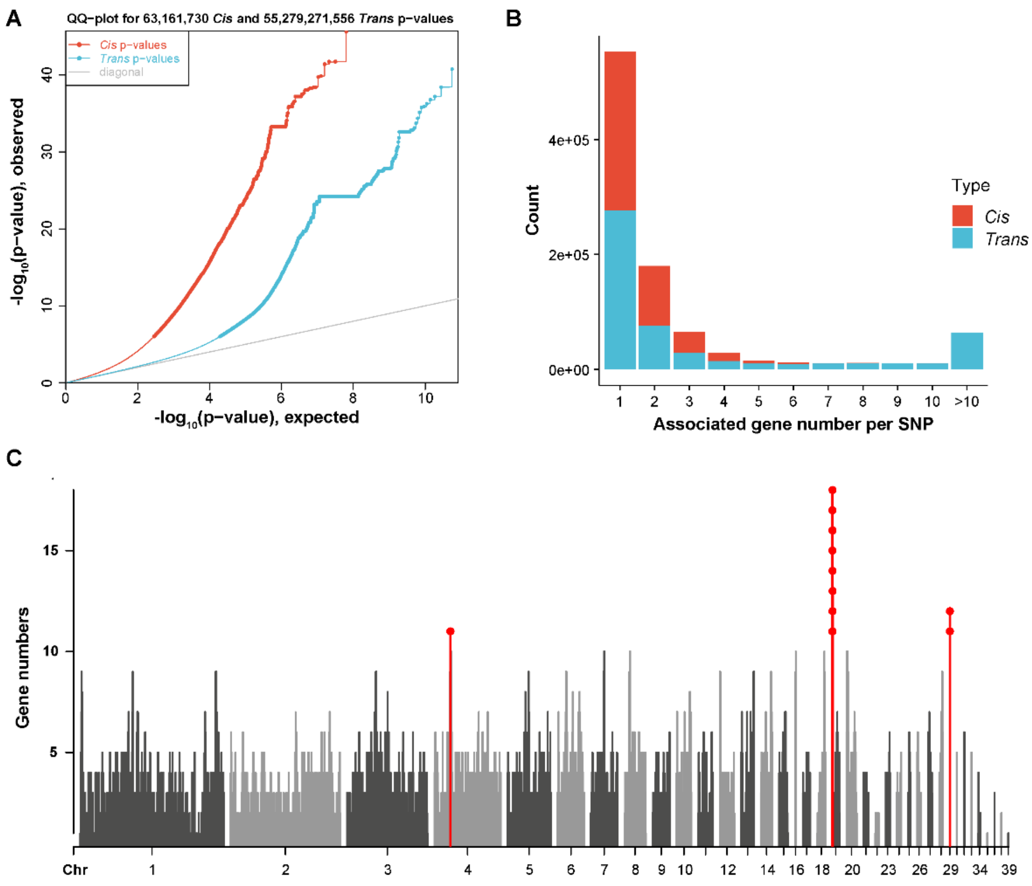

Fig. 4. eQTL profiles based on transcriptome sequencing data of ovary tissue collected at 150, 250, 320, 500, and 700 days of age (n=120). **(A)** QQ-plot of -log$_{10}$(*p*-value) of eQTL analysis. **(B)** Analysis of eQTL pleiotropy. The X-axis of the histogram represents the number of different gene expressions a SNP was associated with and the Y-axis represents the eQTL count **(C)** Distribution of eQTL hotspot for *cis*-eQTLs. The X-axis represents the location on the chromosomes, the Y-axis indicates the number of genes associated with each eQTL. If the number of associated genes is greater than 10, it is shown in red.

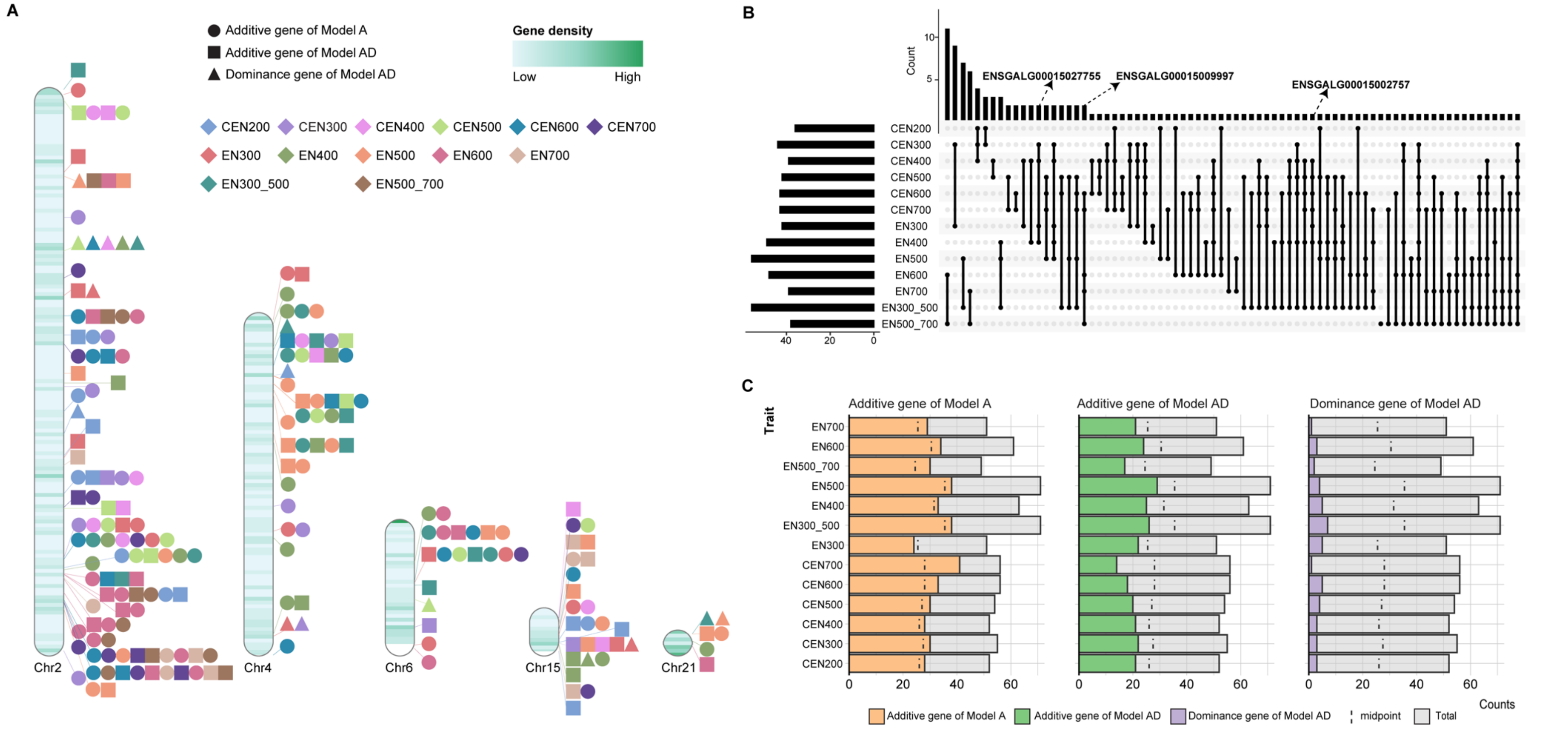

Fig. 5. Genes affecting egg number traits identified by TWAS analysis. **(A)** Statistically significant gene-trait associations identified by S-predixcan. Each association is arranged according to the SNP location on each chromosome and the points are color-coded by traits. Circle represented additive effect from an additive model, box represented additive effect from an additive-dominance model, and triangle represented dominance effect from an additive-dominance model. Gene density was expressed as the averaged number of genes in a 1 Mb window. **(B)** Distribution of

significant genes identified in the TWAS analysis across egg number traits. **(C)** Distribution of significant genes across traits, filtered by models.

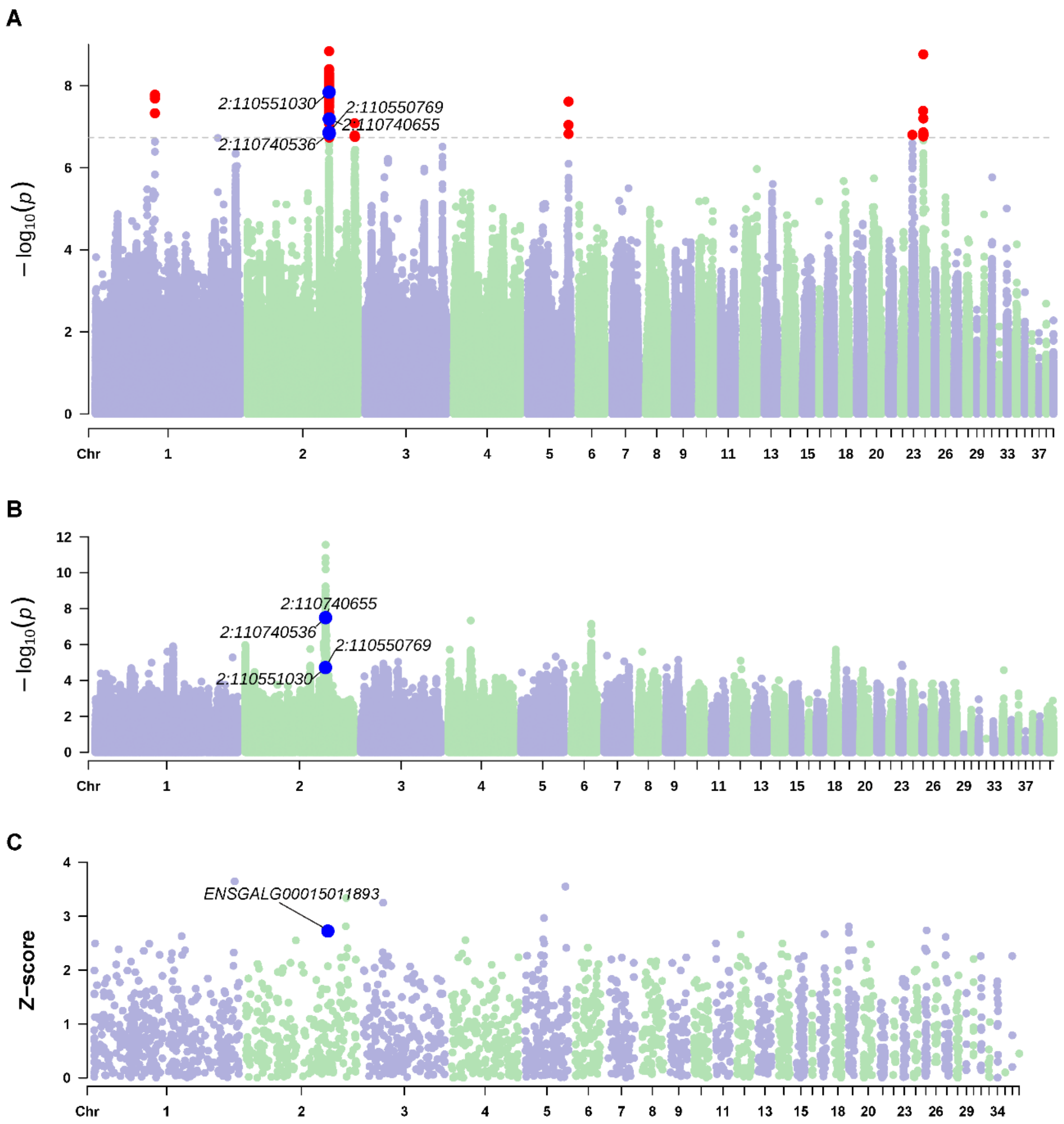

Fig. 6. Multi-omics data analysis of genetic determinants underlying cumulative egg number till 700 days of age. **(A)** GWAS results for trait CEN700. Each dot represent one SNP, and significantly associated SNPs are colored in red with threshold FDR 0.01. Blue dots point out candidate SNPs. **(B)** eQTL mapping for gene *ENSGALG00015011893*. Each dot represent one SNP. Blue dots point out candidate SNPs. **(C)** TWAS results for trait CEN700. Each dot represent one gene. Blue dot point out candidate gene.

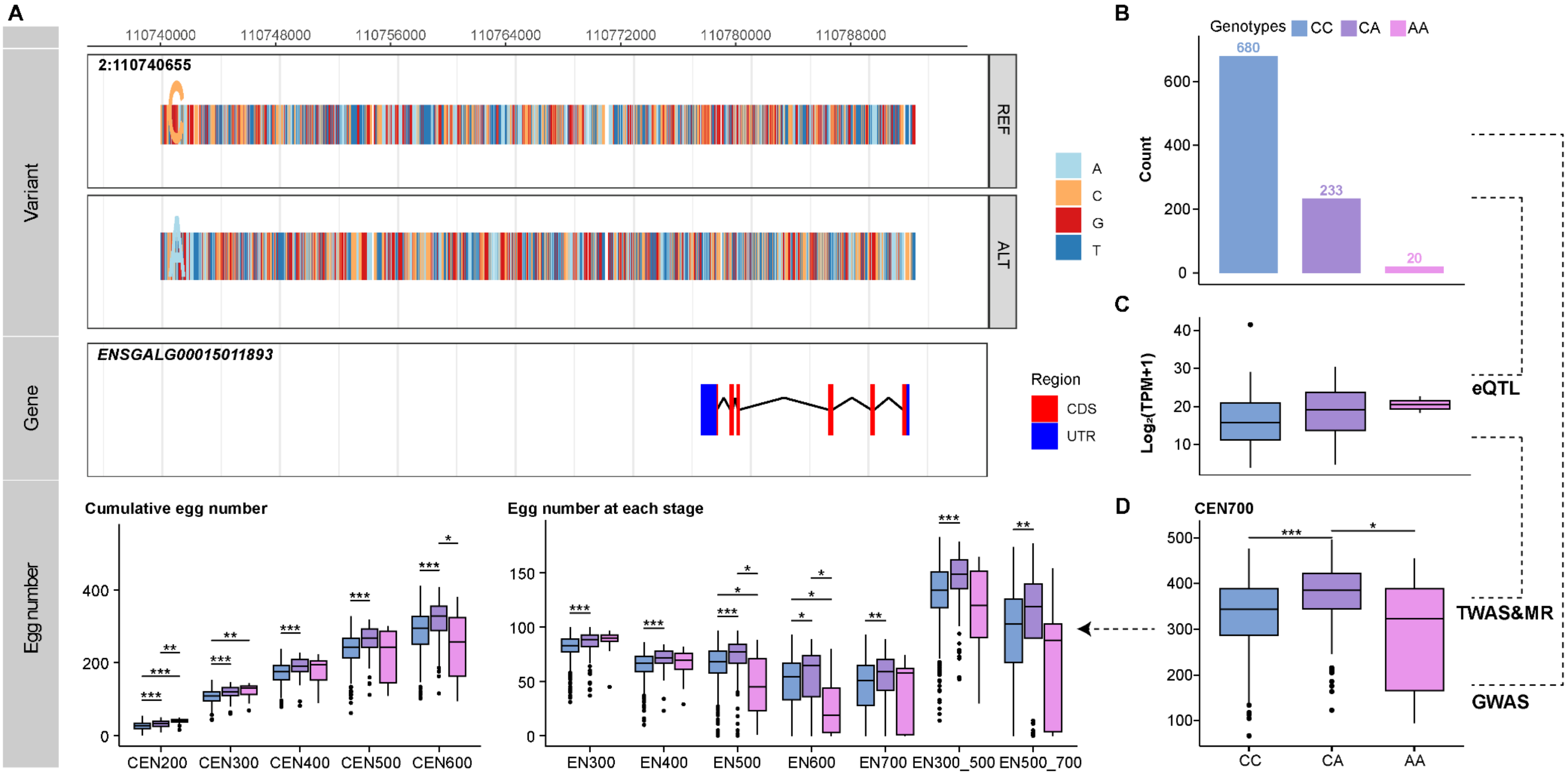

Fig. 7. The associations between candidate genetic variant 2:110740655 and gene expression (*ENSGALG00015011893*) and egg number. **(A)** Schematic representation of the relationship among genetic variant, gene expression, and egg number. The top panel depicts the genomic region from 110.74 Mb to 110.79 Mb, with the highlighted letter indicating SNP 2:110740655, the middle panel illustrates the transcript structure for gene *ENSGALG0015011893*, the bottom panel shows phenotypic changes in egg number across genotypes for SNP 2:110740655 (*: $P < 0.05$, **: $P <$

0.01, ***: $P < 0.001$). **(B)** Counts of different genotypes for SNP 2:110740655. **(C)** Expression level of gene *ENSGALG0015011893* across genotypes for SNP 2:110740655. **(D)** CEN700 across genotypes for SNP 2:110740655 (*: $P < 0.05$, ***: $P < 0.001$).

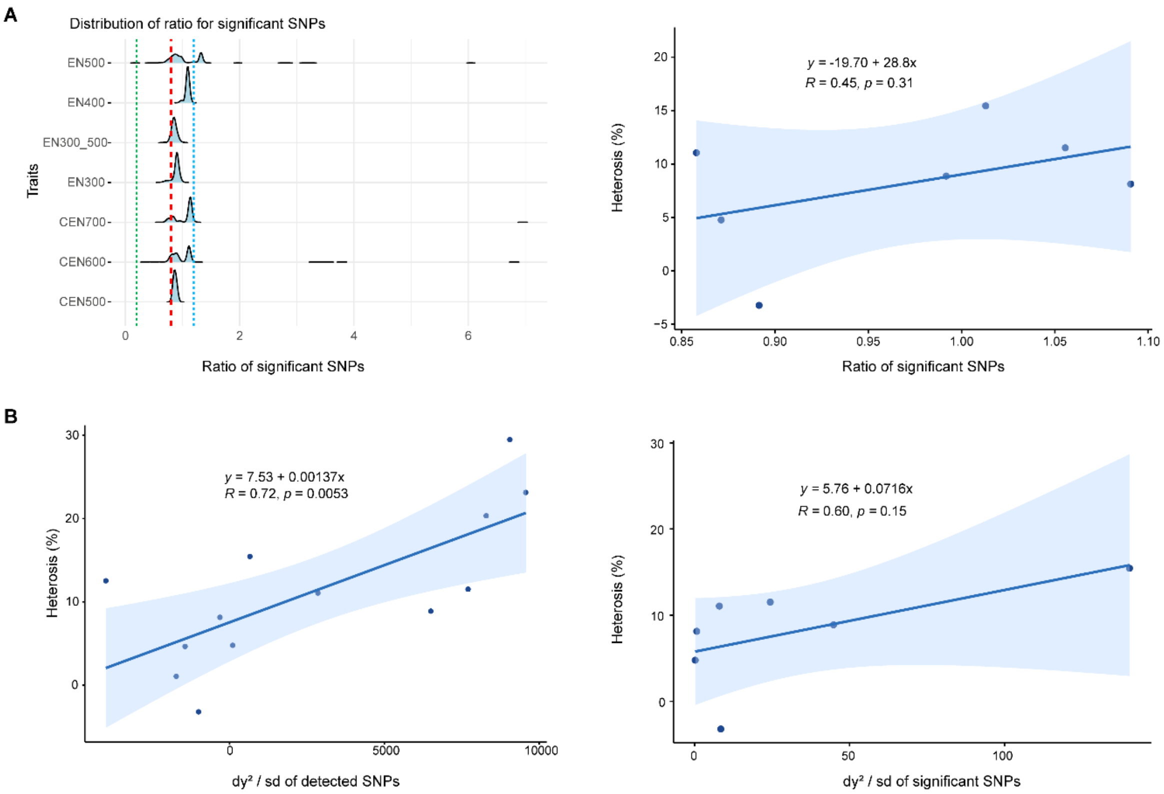

Fig. 8. Implications for heterosis of egg production traits. **(A)** Distribution of ratios for significant SNPs (green dash line: r = 0.2, red dash line: r = 0.8, blue dash line: r = 1.2) and correlation between ratio and heterosis, r is the SNP ratio, $\left|\frac{t_{Dom}}{t_{Add}}\right|$, calculated based on the T-statistics from the Model AD. **(B)** Correlations between the sum of dy^2/sd of all SNPs (left panel) or only the significant SNPs (right panel) and heterosis. Here, *d* represents dominance effects, *y* represents the squared difference in allele frequency, and *sd* represents the phenotypic standard deviation.

Supplementary Material Figures

Click here to access/download
Supplementary Material
Additional_file_AN_20250307.docx

Supplementary Material Tables

Click here to access/download
Supplementary Material
Additonal file Tables_AN_20250307.xlsx

Personal Cover Click here to access/download;Personal Cover;Cover letter_20250307.docx

Dear Editor,

Thanks for your and reviewers' valuable feedback on our submission (GIGA-D-24-00467), "Identifying candidate genetic variants for egg number by analyzing over 1000 fully sequenced layers". We have made modifications according to the reviewers' comments and have highlighted the revised sentences. Additionally, we have updated the public versions of the related code on GitHub.

We confirm that manuscript has not been published elsewhere, and it not under consideration by other journals. All authors have approved the manuscript and agree with its submission to *GigaScience.*

Thank you and with my best regards. We look forward to your response.

Sincerely yours,

Jilan Chen, PhD

Institute of Animal Science,

Chinese Academy of Agricultural Sciences

Beijing 100193, China

Tel: +86-10-62816005

E-mail: chen.jilan@163.com

Graphical Abstract   Click here to access/download;Graphical Abstract;Graphical abstract.tif

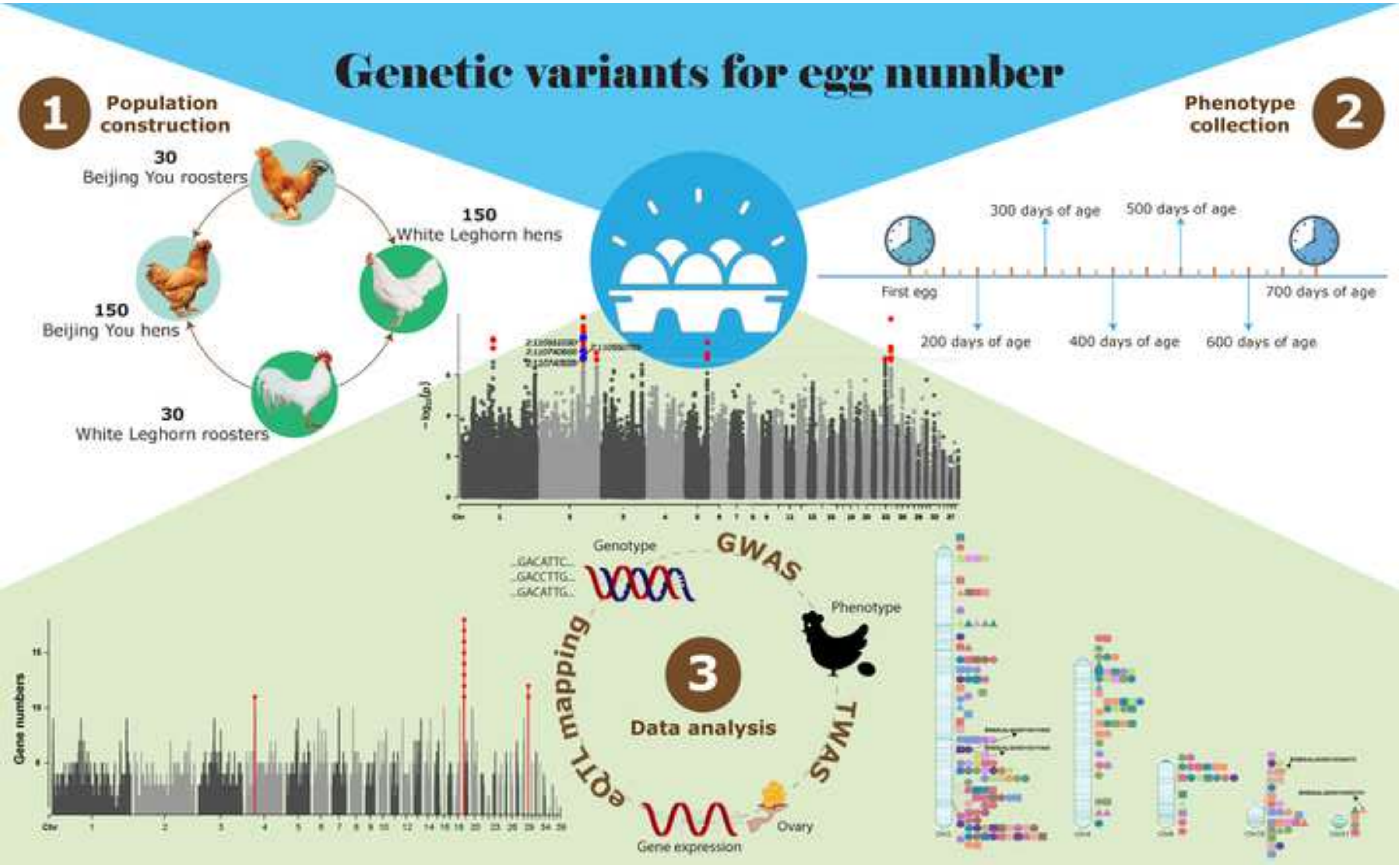

Supplement: giaf064_GIGA-D-24-00467_Revision_1 [file giaf064_giga-d-24-00467_revision_1.pdf]
