# Supplementary material for: Identifying candidate genetic variants for egg number by analyzing over 1,000 fully sequenced layers
Source: Gigascience. 2025 Jun 17;14:giaf064. doi: 10.1093/gigascience/giaf064 (PMC12203006; doi:10.1093/gigascience/giaf064)
Supplement: giaf064_Authors_Response_To_Reviewer_Comments_original_submission [file giaf064_authors_response_to_reviewer_comments_original_submission.pdf]

Author's Response To Reviewer Comments

Reviewer 1:
The study utilized genome and transcriptome data from a full diallel cross of 1,000 hens to conduct GWAS, eQTL mapping, and TWAS analyses. These analyses identified SNP loci and candidate genes that influence egg-laying traits. Incorporating dominant effects into the GWAS model was found to be beneficial. This research is significant for elucidating the molecular genetic mechanisms underlying egg-laying traits and for advancing genetic improvement efforts. However, there are several points in the Methods section that could be further improved:

1.Whole Genome Sequencing:
Why was the SNP filtering criterion for MAF set to less than 0.5%? This threshold seems too low.
Re: We thank the reviewer for the comments. To avoid the missing of rare, yet potentially important alleles, we considered the presence of up to 10 alternate alleles as valid, corresponding to a 0.5% MAF. This is now clarified in L163-165. Additionally, in the GWAS analysis, we applied further criterion: SNPs were eliminated if a genotype was observed in both WW and YY lines with five or fewer animals (L181-184).

2.Genome-Wide Association Study:
Typically, principal components are included in the GWAS model to correct for population stratification. Is the genetic group sufficient to correct for population stratification?
Re: Thanks for the comment. In our GWAS model, we included genetic group as fixed effect and used a relationship matrix to account for population stratification due to family relationships. The genetic groups coincide with the separation of individuals based on the first principal components. We added the PCA results in supplementary Fig. S8 and included relevant content in the main manuscript in L176-180.
What are the results of the GWAS using only the WY and YW populations?
Re: When performing GWAS on the WY and YW populations, we lose power due to decreasing the size of the data. We would like to keep the GWAS analysis with four genetic groups.
Additionally, the G matrix for the u vector is usually constructed using all SNPs except the tested SNP. How was the G matrix constructed in this study?
Re: The G matrix in our study was built for individuals with phenotypes for each trait and using all SNPs (apart from SNPs if a genotype was observed in both WW and YY lines with five or fewer animals for each trait). So we did not remove the tested SNP. We modified the relevant content in L181-184, L202-203 and L205-207.

3.Transcriptome Sequencing:
The data for the remaining 53 animals is not clearly described. Is this whole genome sequencing data?
Re: We have transcriptome sequencing data for ovaries of 120 animals, of which 67 also have whole-genome sequencing data. The remaining 53 animals were genotyped by mapping the transcriptome sequencing data to the reference genome, followed by imputation using the whole-genome sequencing data of the other 933 animas as the reference panel. We added more details about this process, including a comparison between the two data sources in L252-256, and L266-284. When doing this comparison, we further fine-tuned this process, by filtering SNPs from transcriptome sequencing (see L276-280).

4.eQTL Mapping:
Why were sequencing batch and PEERs not considered in the statistical model?
Re: Thanks for pointing out the PEER effects. We modified the eQTL mapping model by incorporating PEERs factors. Additionally, PEERs factors were also included in the construction of the TWAS expression prediction model. Please refer to L289-292, L295-297, and L303 for more details. We updated the results for eQTL-mapping and TWAS accordingly. Please refer to the updated Figs. 4-7 and the relevant content in L365-445, and L545-556.

5.Transcriptome-Wide Association Study and Mendelian Randomization Analysis:
Gene expression data was corrected for age in the study, and predicted expression levels were used in the association for egg numbers at different periods. Was it assumed that gene expression is constant

throughout the entire egg-laying period in TWAS? Why were ovary samples collected for RNA sequencing at different ages? Can analyzing gene expression levels at different stages provide any useful information?
Re: The RNA samples were originally collected for a different experiment aimed at identifying differentially expressed genes at different stages.
In our TWAS analysis, we assumed that the same genotype would lead to consistent gene expression, thereby predicting the genetic contribution to gene expression. We also accounted for potential age-related variations in gene expression by correcting for age effects in our expression data.
Collect RNA samples at different ages would allow us to detect both age-dependent and age-independent genes associated with egg production traits. For instance, ENSGALG00015002757 influenced egg production throughout the entire laying period, ENSGALG00015027755 played a role primarily in early-stage egg production, ENSGALG00015009997 was associated with later-stage egg production. We added the relevant content in L388-396.

Reviewer 2:
Ni et al. sequenced 1004 chickens and delves into the genetic variants linked to egg production by integrating GWAS, TWAS, and eQTL analyses within a four diallel crossed populations. This study represents a significant contribution to our understanding of the genetic underpinnings of this economically vital trait. Nonetheless, the current manuscript has certain limitations that require attention to further enhance its scientific value.

Major comments:
1. Coherence of candidate SNPs, genes, and phenotypes: While the discussion on candidate genes is extensive, there is a notable lack of coherence in linking these candidate SNPs, genes, and phenotypes. Therefore, this section should be reorganized and expanded to enhance clarity and scientific rigor.
Re: Thanks for the comment. We updated the results following the inclusion of PEER factors in the eQTL mapping analysis and worked to improve the coherence, with relevant content added in L545-556. However, due to the lack of other explanatory information such as the annotation for regulatory elements, we are unable to confirm whether the SNPs are located within regulatory regions or have specific functions that impact gene expression (as now mentioned in the added part in the Discussion).

2. cis-eQTL additivity: This paper, with the DOI number of 10.1186/s13059-023-03060-2, suggests that the majority of cis-eQTLs exhibit additive effects. It is highly recommended to conduct further analysis to verify whether a comparable pattern can be observed in the current study, and to augment the discussion section with a more comprehensive analysis of the obtained results.
Re: With our dataset, we conducted the analysis, and observed an even distribution of cis- and trans-eQTLs across the genome for all types of gene action. We added relevant content and potential explanations in the discussion part in L529-542.

3. One key conclusion is that incorporating dominance effects into the GWAS model enhances detection. The authors should discuss whether this approach could be generalized to other traits or even to other species.
Re: Thanks for the comments. We now elaborate more on this in L494-501. We compared the results incorporating dominance into GWAS model, and found inconsistent results. We cannot generalized this to other traits or species.

4. Exclusion of sex chromosome variants: The exclusion of sex chromosome variants from the dataset should be justified.
Re: Since we only have one allele for sex chromosomes (in chickens the female is the heterogametic sex), and, the genotype matrix (and thus the effect) of additive and dominance SNP effects cannot be distinguished in Model AD. We decided to exclude the sex chromosome from our analyses. We added relevant content in the Material and Method part in L221-224.

Minor Comments:
1.Please add the line number in your next manuscript version.
Re: Done as requested.

2.MM-Transcriptome sequencing section. In the 120 samples, genomic data of 67 samples were directly obtained from whole genome sequencing, while the genomic data of the other 53 samples were derived from transcriptome sequencing. Please address the correlation and potential bias between these two methods.
Re: Thanks for the suggestions. We added the relevant content regarding the difference in genotype calling between whole-genome sequencing data and transcriptome data. After comparison, we remove SNPs with high percentage of missing genotypes, which resulted in an increase in concordance. Please refer to L252-256, and L266-284 for more details.

3.Result-Genome-wide association study and candidate variants section. "The number of significant additive and dominance SNPs detected by Model AD was 3,294 and 2,598, while no significant SNPs were detected by Model A". Please explain why so many additive SNPs were detected in the AD model but not a single one was detected in the A model? Shouldn't the A model be much more specific in detecting additive SNPs? Which result should we believe?
Re: In the current study, Fig. 3A shows the highly consistent additive SNP effects across the two models, indicating that additive SNP effects in Model A remained largely unchanged in Model AD. This suggests that both models have similar power to detect additive SNP effects, while the significant SNPs exhibited more extreme effects in Model AD. We considered the possibility that opposing additive and dominance SNP effects in Model A can potential cancel out, as pointed out in Fig. 3B. Our findings suggest that Model AD may be more suitable to detect trait-related variants in a context specific manner. We added some explanations in L351-353 and L482-501 in the revised manuscript.

4.Fig 2D, It appears that the two sections of fig2D complement each other and can be combined into one, forming an "additive + dominance" plot. Please replot the fig2D or explain what is the difference between these two sections and what the "total" mean is?
Re: Thanks for the suggestions. We combined the two sections into one.

5."Among the coding consequences, the most abundant variants are synonymous variants with the similar proportion ~0.70 in significant and all SNPs, which were non-neutral (47)." Please explain the "non-neutral" in here.
Re: Done as requested. Although synonymous variants are typically considered neutral, some studies suggest that these variants may have consequences on the transcriptome process. We added explanations in L341-345.

6."The additive and dominance SNP effects from Model AD are strongly related to each other (Fig. 3A). These patterns were similar for other chromosomes and traits. For all significant SNPs, most of the additive SNP effects are negative, while most of the dominance effects are positive(Fig. 3C)." Please explain why most effects of additive and dominance are oppsite but strongly related to each other.
Re: Thanks for the suggestions. We expected a higher frequency of homozygotes for the reference allele in WW since we used the reference genome of WW. Additionally, WW is a breed known for high egg production, and we coded the genotype based on the alternative allele, these may have resulted in negative additive SNP effects. Additionally, given that the two pure lines are genetically distinct and considering the substantial heterosis observed in the crossbreds in our previous study, the dominance SNP effects for many SNPs were positive. This explains the opposite sign for the two SNP effects. We added some discussion on this in L511-518 in the revised manuscript.
Furthermore, the significant SNPs we detected are beneficial in both homozygous and heterozygous forms, this could explain why we have a strong relationship between additive and dominance SNP effects, which we now added as additional figure Fig. S22 and we have rephrased the content in the manuscript in L518-528.

7.Table 3. The performance of egg production in the two crossed populations resembles that of their paternal lines, with YW resembling YY and WY resembling WW. This phenomenon appears to be more indicative of imprinting rather than typical heterosis. Please explain why the consideration of paternal

imprinting was overlooked.
Re: We added relevant content about the difference of reciprocal crosses, and mentioned some possible reasons that explain this observed difference, including paternal imprinting. Please check L589-595 for more details.

Lines 31-32: Please specify the number of animals with transcriptome data.
Re: Done as requested. Please see L31 in the revised manuscript.

Lines 33-34: Expand the content related to eQTL mapping, as it is an important component of the study.
Re: Done as requested. Please see L33-35 in the revised manuscript.

Lines 37-38: Are "805" and "360" unique counts?
Re: Yes, these represent the unique counts. We changed the word "with" to "comprising" to clarify that they are unique counts. Please see L38-39 in the revised manuscript.

Lines 289-293, 295-297: The dominance effects contribute substantially to the total variance for egg number at different stages but not the cumulative egg number. Please elaborate on this discrepancy in the discussion section.
Re: Thanks for the comment. We agree with your point, however, we did not have sufficient information to fully support our interpretation. One possible explanation is that dominance effects may be age-dependent, with their direction varying over time, which could result in the absence of variance for cumulative egg number at certain stages. Given this limitation, we did not expand on this discussion further in the manuscript.

Line 363: The Manhattan plots for other traits would provide a more comprehensive view.
Re: We added the Manhattan plots for other traits as additional figures in Figs. S19-S21.

Fig. 6: The candidate variants do not appear to be the lead variants in GWAS or eQTL analyses. Please address this.
Re: For the leading variants, no significant expressed genes were identified as being related, suggesting that they may not be expression quantitative trait loci but could influence egg number through other mechanisms. We added some discussion on this in L430-433 in the revised manuscript.

Table 2: Include gene symbols when it is available. Additionally, ensure consistent font sizes across the table, particularly in the last two columns.
Re: Done as requested.

em Editorial Manager®

Cookie Preference Center

We use cookies which are necessary to make our site work. We may also use additional cookies to analyze, improve and personalize our content and your digital experience. For

more information, see our Cookie Policy.

You may choose not to allow some types of cookies. However, blocking some types may impact your experience of our site and the services we are able to offer. See the different category headings below to find out more or change your settings.

Allow all

Manage Consent Preferences

Strictly Necessary Cookies

Always active

These cookies are necessary for the website to function and cannot be switched off in our systems. They are usually only set in response to actions made by you which amount to a request for services, such as setting your privacy preferences, logging in or filling in forms. You can set your browser to block or alert you about these cookies, but some parts of the site will not then work. These cookies do not store any personally identifiable information.

Functional Cookies

These cookies enable the website to provide enhanced functionality and personalisation. They may be set by us or by third party providers whose services we have added to our pages. If you do not allow these cookies then some or all of these services may not function properly.

Performance Cookies

These cookies allow us to count visits and traffic sources so we can measure and improve the performance of our site. They help us to know which pages are the most and least popular and see how visitors move around the site.

Targeting Cookies

These cookies may be set through our site by our advertising partners. They may be used by those companies to build a profile of your interests and show you relevant adverts on other sites. If you do not allow these cookies, you will experience less targeted advertising.

Cookie List

Clear

Apply Cancel

Consent Leg.Interest

Confirm my choices

Powered by onetrust
