# Supplementary material for: Identifying candidate genetic variants for egg number by analyzing over 1,000 fully sequenced layers
Source: Gigascience. 2025 Jun 17;14:giaf064. doi: 10.1093/gigascience/giaf064 (PMC12203006; doi:10.1093/gigascience/giaf064)
Supplement: giaf064_Reviewer_1_Report_Original_submission — Hua Li -- 12/23/2024 [file giaf064_reviewer_1_report_original_submission.pdf]

Reviewer Report

Title: Identifying candidate genetic variants for egg number by analyzing over 1000 fully sequenced layers

Version: Original Submission **Date:** 12/23/2024

Reviewer name: Hua Li

Reviewer Comments to Author:

The study utilized genome and transcriptome data from a full diallel cross of 1,000 hens to conduct GWAS, eQTL mapping, and TWAS analyses. These analyses identified SNP loci and candidate genes that influence egg-laying traits. Incorporating dominant effects into the GWAS model was found to be beneficial. This research is significant for elucidating the molecular genetic mechanisms underlying egg-laying traits and for advancing genetic improvement efforts. However, there are several points in the Methods section that could be further improved:

1.Whole Genome Sequencing:

Why was the SNP filtering criterion for MAF set to less than 0.5%? This threshold seems too low.

2.Genome-Wide Association Study:

Typically, principal components are included in the GWAS model to correct for population stratification. Is the genetic group sufficient to correct for population stratification? What are the results of the GWAS using only the WY and YW populations?

Additionally, the G matrix for the u vector is usually constructed using all SNPs except the tested SNP. How was the G matrix constructed in this study?

3.Transcriptome Sequencing:

The data for the remaining 53 animals is not clearly described. Is this whole genome sequencing data?

4.eQTL Mapping:

Why were sequencing batch and PEERs not considered in the statistical model?

5.Transcriptome-Wide Association Study and Mendelian Randomization Analysis:

Gene expression data was corrected for age in the study, and predicted expression levels were used in the association for egg numbers at different periods. Was it assumed that gene expression is constant throughout the entire egg-laying period in TWAS? Why were ovary samples collected for RNA sequencing at different ages? Can analyzing gene expression levels at different stages provide any useful information?

Methods

Are the methods appropriate to the aims of the study, are they well described, and are necessary controls included? Choose an item.

Conclusions

Are the conclusions adequately supported by the data shown? Choose an item.

Reporting Standards

Does the manuscript adhere to the journal's guidelines on [minimum standards of reporting?](#) Choose an item.

Choose an item.

Statistics

Are you able to assess all statistics in the manuscript, including the appropriateness of statistical tests used? Choose an item.

Quality of Written English

Please indicate the quality of language in the manuscript: Choose an item.

Declaration of Competing Interests

Please complete a declaration of competing interests, considering the following questions:

- Have you in the past five years received reimbursements, fees, funding, or salary from an organisation that may in any way gain or lose financially from the publication of this manuscript, either now or in the future?
- Do you hold any stocks or shares in an organisation that may in any way gain or lose financially from the publication of this manuscript, either now or in the future?
- Do you hold or are you currently applying for any patents relating to the content of the manuscript?
- Have you received reimbursements, fees, funding, or salary from an organization that holds or has applied for patents relating to the content of the manuscript?
- Do you have any other financial competing interests?
- Do you have any non-financial competing interests in relation to this paper?

If you can answer no to all of the above, write 'I declare that I have no competing interests' below. If your reply is yes to any, please give details below.

'I declare that I have no competing interests

I agree to the open peer review policy of the journal. I understand that my name will be included on my report to the authors and, if the manuscript is accepted for publication, my named report including any attachments I upload will be posted on the website along with the authors' responses. I agree for my report to be made available under an Open Access Creative Commons CC-BY license (http://creativecommons.org/licenses/by/4.0/). I understand that any comments which I do not wish to be included in my named report can be included as confidential comments to the editors, which will not be published.

Choose an item.

To further support our reviewers, we have joined with Publons, where you can gain additional credit to further highlight your hard work (see: https://publons.com/journal/530/gigascience). On publication of this paper, your review will be automatically added to Publons, you can then choose whether or not to claim your Publons credit. I understand this statement.

Yes Choose an item.
