# Supplementary material for: Identifying candidate genetic variants for egg number by analyzing over 1,000 fully sequenced layers
Source: Gigascience. 2025 Jun 17;14:giaf064. doi: 10.1093/gigascience/giaf064 (PMC12203006; doi:10.1093/gigascience/giaf064)
Supplement: giaf064_Reviewer_2_Report_Original_submission — Zhenfang Wu -- 12/29/2024 [file giaf064_reviewer_2_report_original_submission.pdf]

Reviewer Report

Title: Identifying candidate genetic variants for egg number by analyzing over 1000 fully sequenced layers

Version: Original Submission **Date:** 12/29/2024

Reviewer name: Zhenfang Wu

Reviewer Comments to Author:

Ni et al. sequenced 1004 chickens and delves into the genetic variants linked to egg production by integrating GWAS, TWAS, and eQTL analyses within a four diallel crossed populations. This study represents a significant contribution to our understanding of the genetic underpinnings of this economically vital trait. Nonetheless, the current manuscript has certain limitations that require attention to further enhance its scientific value.

Major comments:

1. Coherence of candidate SNPs, genes, and phenotypes: While the discussion on candidate genes is extensive, there is a notable lack of coherence in linking these candidate SNPs, genes, and phenotypes. Therefore, this section should be reorganized and expanded to enhance clarity and scientific rigor.
2. cis-eQTL additivity: This paper, with the DOI number of 10.1186/s13059-023-03060-2, suggests that the majority of cis-eQTLs exhibit additive effects. It is highly recommended to conduct further analysis to verify whether a comparable pattern can be observed in the current study, and to augment the discussion section with a more comprehensive analysis of the obtained results.
3. One key conclusion is that incorporating dominance effects into the GWAS model enhances detection. The authors should discuss whether this approach could be generalized to other traits or even to other species.
4. Exclusion of sex chromosome variants: The exclusion of sex chromosome variants from the dataset should be justified.

Minor Comments:

1.Please add the line number in your next manuscript version.

2.MM-Transcriptome sequencing section. In the 120 samples, genomic data of 67 samples were directly obtained from whole genome sequencing, while the genomic data of the other 53 samples were derived from transcriptome sequencing. Please address the correlation and potential bias between these two methods.

3.Result-Genome-wide association study and candidate variants section. "The number of significant additive and dominance SNPs detected by Model AD was 3,294 and 2,598, while no significant SNPs were detected by Model A". Please explain why so many additive SNPs were detected in the AD model but not a single one was detected in the A model? Shouldn't the A model be much more specific in detecting additive SNPs? Which result should we believe?

4.Fig 2D, It appears that the two sections of fig2D complement each other and can be combined into one, forming an "additive + dominance" plot. Please replot the fig2D or explain what is the difference between these two sections and what the "total" mean is?

5."Among the coding consequences, the most abundant variants are synonymous variants with the similar proportion ~0.70 in significant and all SNPs, which were non-neutral (47)." Please explain the "non-neutral" in here.
6."The additive and dominance SNP effects from Model AD are strongly related to each other (Fig. 3A). These patterns were similar for other chromosomes and traits. For all significant SNPs, most of the additive SNP effects are negative, while most of the dominance effects are positive(Fig. 3C)." Please explain why most effects of additive and dominance are oppsite but strongly related to each other.
7.Table 3. The performance of egg production in the two crossed populations resembles that of their paternal lines, with YW resembling YY and WY resembling WW. This phenomenon appears to be more indicative of imprinting rather than typical heterosis. Please explain why the consideration of paternal imprinting was overlooked.
Lines 31-32: Please specify the number of animals with transcriptome data.
Lines 33-34: Expand the content related to eQTL mapping, as it is an important component of the study.
Lines 37-38: Are "805" and "360" unique counts?
Lines 289-293, 295-297: The dominance effects contribute substantially to the total variance for egg number at different stages but not the cumulative egg number. Please elaborate on this discrepancy in the discussion section.
Line 363: The Manhattan plots for other traits would provide a more comprehensive view.
Fig. 6: The candidate variants do not appear to be the lead variants in GWAS or eQTL analyses. Please address this.
Table 2: Include gene symbols when it is available. Additionally, ensure consistent font sizes across the table, particularly in the last two columns.

Methods

Are the methods appropriate to the aims of the study, are they well described, and are necessary controls included? Choose an item.

Conclusions

Are the conclusions adequately supported by the data shown? Choose an item.

Reporting Standards

Does the manuscript adhere to the journal's guidelines on minimum standards of reporting? Choose an item.

Choose an item.

Statistics

Are you able to assess all statistics in the manuscript, including the appropriateness of statistical tests used? Choose an item.

Quality of Written English

Please indicate the quality of language in the manuscript: Choose an item.

Declaration of Competing Interests

Please complete a declaration of competing interests, considering the following questions:

- Have you in the past five years received reimbursements, fees, funding, or salary from an organisation that may in any way gain or lose financially from the publication of this manuscript, either now or in the future?
- Do you hold any stocks or shares in an organisation that may in any way gain or lose financially from the publication of this manuscript, either now or in the future?
- Do you hold or are you currently applying for any patents relating to the content of the manuscript?
- Have you received reimbursements, fees, funding, or salary from an organization that holds or has applied for patents relating to the content of the manuscript?
- Do you have any other financial competing interests?
- Do you have any non-financial competing interests in relation to this paper?

If you can answer no to all of the above, write 'I declare that I have no competing interests' below. If your reply is yes to any, please give details below.

I declare that I have no competing interests

I agree to the open peer review policy of the journal. I understand that my name will be included on my report to the authors and, if the manuscript is accepted for publication, my named report including any attachments I upload will be posted on the website along with the authors' responses. I agree for my report to be made available under an Open Access Creative Commons CC-BY license (http://creativecommons.org/licenses/by/4.0/). I understand that any comments which I do not wish to be included in my named report can be included as confidential comments to the editors, which will not be published.

Choose an item.

To further support our reviewers, we have joined with Publons, where you can gain additional credit to further highlight your hard work (see: https://publons.com/journal/530/gigascience). On publication of this paper, your review will be automatically added to Publons, you can then choose whether or not to claim your Publons credit. I understand this statement.

Yes Choose an item.
